# Publisher Correction: Clarifying the effect of biodiversity on productivity in natural ecosystems with longitudinal data and methods for causal inference

**DOI:** 10.1038/s41467-023-39743-4

**Published:** 2023-07-12

**Authors:** Laura E. Dee, Paul J. Ferraro, Christopher N. Severen, Kaitlin A. Kimmel, Elizabeth T. Borer, Jarrett E. K. Byrnes, Adam Thomas Clark, Yann Hautier, Andrew Hector, Xavier Raynaud, Peter B. Reich, Alexandra J. Wright, Carlos A. Arnillas, Kendi F. Davies, Andrew MacDougall, Akira S. Mori, Melinda D. Smith, Peter B. Adler, Jonathan D. Bakker, Kate A. Brauman, Jane Cowles, Kimberly Komatsu, Johannes M. H. Knops, Rebecca L. McCulley, Joslin L. Moore, John W. Morgan, Timothy Ohlert, Sally A. Power, Lauren L. Sullivan, Carly Stevens, Michel Loreau

**Affiliations:** 1 Department of Ecology and Evolutionary Biology, University of Colorado, Boulder, CO USA; 2 Department of Environmental Health and Engineering, Bloomberg School of Public Health & Whiting School of Engineering, Johns Hopkins University, Baltimore, MD USA; 3 Carey Business School, Johns Hopkins University, Baltimore, MD USA; 4 Research Department, Federal Reserve Bank of Philadelphia, Philadelphia, PA USA; 5 Department of Ecology, Evolution, and Behavior, University of Minnesota, St. Paul, MN 55108 USA; 6 Department of Biology, University of Massachusetts Boston, 100 Morissey Blvd, Boston, MA 02125 USA; 7 Institute of Biology, University of Graz, Holteigasse 6, 8010 Graz, Austria; 8 Ecology and Biodiversity Group, Department of Biology, Utrecht University, Padualaan 8, 3584 CH Utrecht, The Netherlands; 9 Department of Plant Sciences, University of Oxford, Oxford, OX1 3RB UK; 10 Sorbonne Université, Université Paris Cité, UPEC, IRD, CNRS, INRA, Institute of Ecology and Environmental Sciences, iEES Paris, Paris, France; 11 Institute for Global Change Biology, and School for Environment and Sustainability, University of Michigan, Ann Arbor, MI USA; 12 Department of Forest Resources, University of Minnesota, St. Paul, MN 55108 USA; 13 Hawkesbury Institute for the Environment, Western Sydney University, Penrith, NSW 2751 Australia; 14 Department of Biological Sciences, California State University Los Angeles, Los Angeles, CA USA; 15 Department of Physical and Environmental Sciences, University of Toronto at Scarborough, Toronto, 1265 Military Trail, ON M1C 1A4 Canada; 16 Department of Integrative Biology, University of Guelph, Guelph, Ontario, N1G 2W1 Canada; 17 Research Center for Advanced Science and Technology, The University of Tokyo, 4-6-1 Komaba, Meguro, Tokyo, 153-8904 Japan; 18 Department of Biology, Colorado State University, Fort Collins, CO 80523 USA; 19 Graduate Degree Program in Ecology, Colorado State University, Fort Collins, CO 80523 USA; 20 Department of Wildland Resources and the Ecology Center, Utah State University, Logan, UT 84322 USA; 21 School of Environmental and Forest Sciences, University of Washington, Box 354115, Seattle, WA 98195-4115 USA; 22 Global Water Security Center, The University of Alabama, Box 870206, Tuscaloosa, AL 35487 US; 23 Smithsonian Environmental Research Center, Edgewater, MD 21037 USA; 24 Department of Health and Environmental Sciences, Xián Jiaotong-Liverpool University, Suzhou, China; 25 Department of Plant and Soil Sciences, University of Kentucky, Lexington, KY 40546-0312 USA; 26 School of Biological Sciences, Monash University, Clayton, VIC 3800 Australia; 27 Department of Ecology, Environment and Evolution, La Trobe University, Bundoora, VIC 3086 Australia; 28 Department of Plant Biology, Michigan State University, East Lansing, MI 48824 USA; 29 Kellogg Biological Station, Michigan State University, Hickory Corners, MI 49060 USA; 30 Lancaster Environment Centre, Lancaster University, Lancaster, LA1 4YQ UK; 31 Theoretical and Experimental Ecology Station, CNRS, 09200 Moulis, France

**Keywords:** Biodiversity, Community ecology, Grassland ecology, Ecological modelling

Correction to: *Nature Communications* 10.1038/s41467-023-37194-5, published online 05 May 2023

The original version of this Article contained errors in the Methods section ‘Target causal effect’, in which terms were omitted from the mathematical definitions of the causal effect and average causal effect. These sentences incorrectly read “The causal effect of a change in richness from *R*′ to *R*″ on productivity *P* in plot *i* is defined as [(*R*″) − (*R*′)], where *P*_*i*_(*R*″) is the potential productivity outcome when *R* = *R*″ and *P*(*R*′) is the potential productivity outcome when *R* = *R*′ (*R*′ ≠ *R*″).” and “The average causal effect of a change in biodiversity from *R*′ to *R*″ across all plots is [(*R*″) − *P*(*R*′)], where *E*[·] is the expectation operator.”. The correct version states “[*P*_*i*_(*R*′′) − *P*_*i*_(*R*′)]” in place of “[(R″) − (R′)]”, “*P*_*i*_ (*R*′)” in place of “*P* (*R*′)”, and “*E*[*P*_*i*_(*R*′′) − *P*_*i*_(*R*′)]” in place of “[(R″) − P(R′)]”. This has been corrected in both the PDF and HTML versions of the Article.

