# Supplementary material for: Clarifying the effect of biodiversity on productivity in natural ecosystems with longitudinal data and methods for causal inference
Source: Nat Commun. 2023 May 5;14:2607. doi: 10.1038/s41467-023-37194-5 (PMC10163230; doi:10.1038/s41467-023-37194-5)
Supplement: Supplementary file 1 — Supplementary Information [file 41467_2023_37194_MOESM1_ESM.pdf]

Supplementary Information (SI) for Dee et al. "Clarifying the effect of biodiversity on productivity in natural ecosystems with longitudinal data and methods for causal inference."

Data and Code Availability: The code for reproducing all analyses, figures, and tables in this study are available at https://github.com/LauraDee/NutNetCausalinf and released on Zenodo (DOI/10.5281/zenodo.7675340), which we refer to in this SI as our "project page." An RMarkdown tutorial on the methods can also be found on our Zenodo release and as a Supplemental Data File with this publication.

Table of Contents

Supplemental Methods 3

S1. Glossary of Terms 3

S2. Directed Acyclic Causal Graph (Figure 1B) 7

S2a. Comparison to path diagrams and structural equation models 7

S3. Supplementary Methods: Data Description 8

S3a. Measuring Biodiversity and Productivity 9

S3b. Data transformations 10

S4. Supplementary Methods: Main Design Estimator 13

S4a. Review of Main Design from Methods section 13

S4b. Estimation procedure and implementation 15

S4c. Brief comparison of our design and aims to other study designs and aims 15

S5. Supplementary Methods: Extensions of the Main Design Estimator 16

S5a. Species evenness 17

S5b. Functional form 18

S5c. Moderating effect of site-level species richness 19

S5d. Moderating effect of site-level productivity 20

S6. Supplementary Methods: Robustness Checks to Assess Potential Threats to Internal Validity 23

S6a. Reverse causality: productivity causes species richness 24

S6a.i. Blocked mechanism design *25*
S6a.ii. Instrumental variable design *27*
S6bi. Dynamic panel designs *31*
S6b.ii. Design sensitivity to unobserved, plot-level confounding variables *37*

S7. Comparison of Main Design to Common Designs in Ecology 41

S8. Supplementary Methods: Heterogeneous Effects of Rare, Non-rare, and Non-native Species on Productivity 45
S8a. Definitions and Measurement of groups in Figure 5 .... *47*
S8b. Statistical Analyses *49*
S8c. Comparing the effect of species richness per group on productivity *50*
S8ci. Sensitivity Analyses for species with unknown origins *51*
S8cii. Sensitivity analyses using relative frequency as a metric for rarity *52*
S8ciii. Sensitivity Analyses using different cut-offs for rare versus non-rare categories *54*
S8d. Variation in each species group *55*

Supplementary Discussion 58

S9. Nine Frequently Asked Questions (FAQ) about Dee et al. 58

S10. Supplementary References 76

Supplemental Methods

S1. Glossary of Terms

We provide brief definitions of terms, and we organize the terms logically rather than alphabetically. For more details and background reading, see e.g., (*1*–*7*).

Counterfactual (contrary to fact): In the definition of a causal effect, a plot is assumed to have a potential productivity outcome under each potential richness level; e.g., $P_i(R'')$ is the potential productivity outcome when $R = R''$ and $P(R_i')$ is the potential productivity outcome when $R = R'$ ($R' \neq R''$). But at any point in time, only one of those richness levels, and thus one of those productivity values, will be observed. The other values are counterfactual values – i.e., the productivity values that would have been observed had we instead observed the plot under the other possible richness levels.

Treatment: Causal variables, like *R*, are often labeled "treatment variables" whether they are manipulated by an experimenter or by nature. A change from one value to another is often labeled a "treatment." See (*6*–*8*).

Average Treatment Effect (ATE): The ATE of biodiversity on productivity in plot *i* in year *t* is defined as $\mathrm{E}[P_{it}(R'') - P_{it}(R')]$, where $\mathrm{E}[P_{it}(R'')]$ is the expected productivity in plot *i* in year *t* when richness has the value $R = R''$ and $\mathrm{E}[P_{it}(R')]$ is the expected productivity in plot *i* in year *t* when richness has the value $R = R'$ ($R' \neq R''$). The ATE is the average (or expected) causal effect of R on P for a randomly selected plot from the study population when biodiversity goes from R' to R''.

Directed Acyclic Causal Graph (DAG): A DAG is a visualization of qualitative causal assumptions on which one relies for making causal claims from observable data (*9*). See Section S2 for more information and the relationship between a DAG and a "path diagram."

Internal Validity: The extent to which a study design allows one to infer a causal relationship from a correlation by ruling out rival explanations. For instance, are the changes in the independent variable, *X*, causing a change in the dependent variable, *Y*, or can those changes in *Y* be attributed to other causes?

External Validity: The extent to which inferences can be generalized (e.g., across sites, time periods, contexts, or scales).

Construct validity: The extent to which an experimental treatment or statistical estimate matches the phenomenon it intends to measure (*10*, *11*) or the theory it intends to test.

Confounding Variables (or "confounders"): The term "confounding variable" describes variables that are systematically correlated with the causal variable (e.g., biodiversity) and the outcome variable (e.g., productivity), and thus can mask or mimic a causal effect. Confounding variables are a potential source of *bias* in a study design.

Bias and Hidden Bias: An estimator is a rule or procedure for calculating an estimate of a causal effect based on observed data. Bias is a property of an estimator: it captures the difference between the estimator's expected value and the true value of the causal effect being estimated (*12*). The phrase "hidden bias" (also called "unobserved heterogeneity") is often used to describe the potential sources of bias in a study design (e.g., an omitted third variable that affects both biodiversity and productivity). Hidden bias is thus a rival explanation for detecting or failing to detect a correlation between a purported causal variable and its outcome using observable data (*reviewed in* (*13*)). The goal of causal analysis is to choose data and a design so that an actual causal effect would be visibly different from the most plausible hidden biases. Note: Sampling variability ("noise" or "chance") is different from hidden bias. Sampling variability is a rival explanation for observed relationships between variables, but it is not a source of bias. Sampling variability declines with more data, whereas bias does not (*14*, *15*). Sampling variability is reflected in variable I in Figure 1B, whereas bias comes from variable U.

"*Selection on Observables" Assumption:* Informally, this assumption implies that confounding variables that could introduce bias are known and observable to the researcher, so that statistical bias can be eliminated (controlled, blocked) by conditioning strategies, such as regression, matching or stratification methods. To read more, see (*1*).

Fixed Effect: Our use of the term "fixed effect" is drawn from the econometrics literature, where it refers to the effect of a time-invariant attribute of the system (*12*); e.g., a plot-level fixed effect is an attribute of the plot that is assumed to not change over the study period, such as topography

or historical patterns of land use. This use of the term "fixed effect" differs from how the term is typically used in ecology, where the term often refers to the coefficient estimates of explanatory variables in mixed (multi-level) modeling (e.g., (*16*)). Further confusing matters, the "random effects" components of a mixed effects model, which describe categorical variables that are assumed to be drawn from a normal distribution with zero mean, are often used to accomplish the same goal as the "fixed effects" that we apply here (e.g. to remove spurious plot-level effects). However, unlike "random effects," econometric fixed effects are not constrained to be drawn from any predefined distribution. They are assumed to be fixed and estimable rather than assumed to have a distribution (i.e., they are not part of the error term, as random effects are assumed to be in multi-level modeling). Operationally, fixed effects in econometrics are simply regression parameters describing categorical or dummy variables per study unit (e.g., in experiments, a categorical fixed effect parameter per plot is often fit to control for differences among plots that are not associated with the experimental treatment). Although this fixed-effect estimation approach comes at the cost of reduced statistical power, it avoids the potential bias that can arise when controlling for time-invariant variables using random effects (to read more, see (*3*)). The use of random effects requires the assumption that the random effect is uncorrelated with all of the covariates in the model (*17*). In an observational data set with any sort of environmental gradient, that assumption is strong and not likely satisfied.

Mechanism: A mechanism is a variable that lies on the causal path between two other variables and mediates the causal effect of one of those variables on the other (*18*); shown as "M" in Figure 1 B (right panel). A mechanism can be viewed as an intermediate outcome of a causal variable; e.g., an increase in plot productivity causes a decrease in plot biodiversity by increasing the amount of shading in the plot – shading is the mechanism through which a change in productivity can cause a change in biodiversity.

Moderator: A moderator is a variable that lies off the causal path between two other variables but moderates the magnitude of a causal effect. A moderator is a source of heterogenous causal effects (*18*); for instance, the degree to which biodiversity affects productivity may depend on weather (e.g., precipitation or temperature) – weather can moderate the causal effect of biodiversity on productivity, but the change in biodiversity does not change weather conditions.

Heterogeneous Treatment: This concept goes by many names in the causal inference literature, including "multiple versions of the treatment", "treatment variation," "hidden versions", "heterogeneous treatments," and "hidden treatments" ("hidden treatments" being used differently from how ecologists have used the phrase in the past (*19*)). This issue can be viewed as a challenge with construct validity: if you say that richness goes from 4 species to 8 species in plot A, and I say that richness goes from 4 species to 8 species in plot B, we need to assess if we are talking about the same change in the treatment variable. In this case, a richness change from 4 to 8 species can involve many combinations in species identity, even in experiments manipulating a subset of all species in an ecosystem (e.g., are all four additional species native and not rare, or are 2 of those additional species rare and 2 non-native?). The underlying idea is that, for a unit of observation (e.g., a plot), there ought to be one potential outcome for each treatment value (e.g., one potential productivity value for each richness value at a particular moment in time). If there is not, we have multiple versions of the same treatment. In this way, species composition is not a confounding variable, but a heterogenous treatment in the effect of richness on productivity. Note that this concept of heterogeneous treatments is different from "heterogeneous treatment effects," which simply means that not every unit responds the same way to a change in the treatment variable (i.e., treatment effects are moderated by variables that differ across units in the study population).

Instrumental variable: This term is defined in Figure 1B as variable *Z*, a variable that affects the treatment variable (in our study, "species richness") but has no direct effect on the outcome variable (in our study, "aboveground biomass") except through its effect on the treatment. In a randomized experiment, the instrumental variable is the randomization procedure. To read more, see (*20*–*22*) and for examples in ecology see (*23*, *24*).

Panel data: Panel data are longitudinal data comprising repeated measures taken from a sample of cases (e.g., plots, sites, regions). These data also called cross-sectional time series (*25*) or longitudinal multilevel data. See section S3 for more detail.

Cross-sectional data: Data without repeated measures taken from a sample. Instead, one measure is taken from each unit of analysis (e.g., plot). For example, when using cross-sectional data, analyses of the effect of biodiversity on productivity have only one observation per plot.

Errors versus residuals: The error term in a regression model represents how the observations differ from the true population. It is an unobservable and part of the true data generating process. In contrast, residuals are an estimate of the unobservable error term, as the difference between the regression line (predicted value) and the observed data points from the sample population. Residuals cannot be used to assess potential bias in an estimation procedure, and thus this distinction is important in our discussions of statistical bias.

S2. Directed Acyclic Causal Graph (Figure 1B)

Figure 1B (right panel) is known as a directed acyclic causal graph (DAG) and is a visualization of qualitative causal assumptions (*5*, *9*, *26*–*28*). A DAG encodes knowledge and beliefs about how a system works. The graphical relations depicted in the DAG encode causal claims – not just representations of associations. A directed edge (e.g., R → P) depicts a claim about the results of many hypothetical experiments, whereby if every other variable represented in the graph is held fixed, R and P will covary if R if manipulated, but not if P is manipulated (note, time is implicit in the DAG, and a DAG assumes that one can isolate the effect of R on P but does not imply that P can never affect R; another DAG may represent the reverse direction, P → R).

One key benefit of a DAG is that it makes transparent the assumptions on which one relies for making causal claims from observable data. A DAG therefore allows the researcher and the reader to better judge the credibility of the causal claims from a specific research design. Another way to view this benefit is that a causal graph helps identify the sources of variation in a causal variable and in its outcome, thereby emphasizing potential sources of bias that must be addressed in a research design and pointing to designs that can address these sources of bias (*1*).

S2a. Comparison to path diagrams and structural equation models

A DAG is like a “path diagram,” which may be more familiar to ecologists and are often used in structural equation modeling (*29*, *30*). Although not all path diagrams are DAGs, a DAG can be interpreted as a non-parametric structural equation model (SEM) (*31*) with no cycles (no double-headed arrows). In other words, an SEM can be a DAG, but an SEM could also contain both cyclic and directed cycles (not a DAG).

In practice, however, SEMs, when used for causal claims, rely on conditioning on observable confounding characteristics to eliminate non-causal dependencies between two variables (‘the

selection on observables assumption' – see S1 Glossary). However, DAGs emphasize also making transparent assumptions about unobservable confounding variables. In contrast to the common practice of SEMs in ecology, our design can eliminate unobserved confounders (section S5). Nevertheless, SEMs, as typically implemented in ecology, have advantages over our design in cases where a researcher believes that all important confounders can be observed and controlled within the SEM: in those cases, SEMs can be more efficient (i.e., higher statistical power) and they expand the scope of analyses that can be performed with a single data set and estimation strategy.

S3. Supplementary Methods: Data Description

We analyze panel data from grasslands around the world in the Nutrient Network (*32*, *33*), which includes mesic grasslands and prairies, savanna, desert grasslands, montane meadows, old fields, and alpine tundra. We use data from 43 sites with unmanipulated plots with at least 5 years of data in the period 2007-2017 (see **Table S1**). Unmanipulated plots are control plots in the nutrient addition experiments of the Network, meaning they receive no additional nutrients. Unprocessed data versions were 'full-cover-09-April-2018.csv', and 'comb-by-plot-clim-soil-diversity-09-Apr-2018.csv' from the Nutrient Network. All R scripts to process data and create derived data is available at the project page (DOI/10.5281/zenodo.7675340).

Table S1. Information on unmanipulated control plots from the Nutrient Network. The table shows the number of plots with data by year and site. All plots in the analysis have at least 5 years of data between 2007-2017. The dataset includes sites from 11 countries and 5 continents (North America, Australia, Europe, South America, and Africa).

Site Name	2007	2008	2009	2010	2011	2012	2013	2014	2015	2016	2017
Bogong	-	-	3	3	3	3	3	3	3	3	3
Boulder South Campus	-	2	2	2	2	2	2	-	-	-	-
Bunchgrass (Andrews LTER)	3	3	2	3	3	3	3	3	3	3	3
Burrawan	-	3	3	3	3	3	3	3	3	-	-
Cedar Creek LTER	5	5	5	5	5	5	5	5	5	5	5
Cedar Point Biological Station	6	6	6	6	6	6	6	6	6	6	-
CEREEP - Ecotron IDF	-	-	-	-	-	3	3	3	3	3	3
Chichaqua Bottoms	-	-	6	6	6	6	6	6	6	6	-
Companhia das Lezirias	-	-	-	-	-	3	3	3	3	3	-

Cowichan	3	3	3	3	3	3	3	3	3	-	-
Doane College Spring Creek Prairie	-	-	-	-	-	2	2	2	2	2	2
Duke Forest	3	3	3	3	3	-	-	-	-	-	-
Elliott Chaparral	-	-	3	3	3	3	3	2	3	3	-
Fruebuel	-	3	3	3	3	3	3	-	3	-	-
Hall's Prairie	3	3	3	3	3	3	3	3	-	-	-
Hart Mountain	3	3	3	3	3	3	-	-	-	-	-
Heronsbrook (Silwood Park)	-	3	3	3	3	3	-	-	-	-	-
Hopland REC	3	3	3	3	3	3	3	3	3	3	3
Kinypanial	-	-	3	3	3	3	3	3	3	-	-
Koffler Scientific Reserve, Joker's Hill	-	-	-	9	9	9	9	9	9	9	9
Konza LTER	3	3	3	3	3	3	-	3	3	-	-
Lancaster	-	3	3	3	3	-	-	3	3	3	-
Lookout (Andrews LTER)	3	2	3	3	3	3	3	3	3	3	3
Mar Chiquita	-	-	-	-	3	3	3	3	3	3	-
Mclaughlin UCNRS	3	3	3	3	3	3	3	3	3	3	3
Mt. Caroline	-	4	4	4	4	4	4	4	4	4	-
Papenburg	1	1	1	1	1	1	1	-	-	-	-
Rookery (Silwood Park)	-	3	3	3	3	3	-	-	-	-	-
Sagehen Creek UCNRS	3	3	3	3	3	3	3	-	-	-	-
Saline Experimental Range	-	3	3	3	3	3	3	3	3	-	-
Savannah River	2	2	2	2	2	2	-	-	-	-	-
Sedgwick Reserve UCNRS	6	6	6	6	6	6	6	6	6	6	6
Serengeti	-	3	3	3	3	3	-	-	-	-	-
Sevilleta LTER	5	5	5	5	5	5	5	5	-	-	-
Sheep Experimental Station	4	4	4	4	4	4	-	-	-	4	-
Shortgrass Steppe LTER	3	3	3	3	3	3	3	3	3	3	-
Sierra Foothills REC	3	5	5	5	5	5	5	5	5	5	5
Smith Prairie	3	3	3	3	3	3	-	-	3	-	-
Spindletop	3	3	3	3	3	3	3	3	3	3	3
Temple	4	4	4	4	4	4	4	4	4	4	-
Trelease	-	-	3	3	3	3	3	-	-	-	-
Ukulinga	-	-	6	6	6	6	6	6	6	6	-
Val Mustair	-	3	3	3	3	3	3	3	3	3	-

S3a. Measuring Biodiversity and Productivity

To measure productivity, we use plant above-ground live mass (biomass) as in **Figure S1.** Biomass production supports many ecosystem processes and services and this measure of productivity has been widely used in addressing the relationship between diversity and productivity with observational data (e.g., (*34–36*)) and in many grassland experiments (*reviewed in* (*37–39*)). For herbaceous vegetation, above-ground live biomass provides a reasonable estimate of primary productivity (*40*).

Live aboveground biomass is measured in the Nutrient Network dataset with the following procedure. In each 5m x 5m unmanipulated control plot, a permanently marked, randomly located, 1m x 1m subplot is sampled annually at peak biomass for species composition. Visual cover estimates are made to the nearest 1% for every species contained within (or over-hanging) the subplot and used to calculate species diversity metrics (richness, evenness). Biomass samples are collected from two 1 m x 0.1 m strips (totaling 0.2 m^2) located adjacent to the 1m^2 cover subplot. All vegetation from plants rooted within these strips is clipped at ground level. The location of the biomass plots changes yearly to avoid repeat sampling previously clipped areas. Biomass is dried at 60°C to constant mass and weighed to the nearest 0.01g. Multiplying weights by five generates a gram per square meter value for productivity.

To make our study comparable with previous studies, we measure biodiversity as species richness, the number of species in a plot in each year (Figures S1 and S2). We also consider (Table S2) analyses that include species evenness, measured as the degree of similarity in abundance between species within a community (*41*), and analyses that measure diversity with Simpson's Diversity. We calculate Simpson diversity as the inverse Simpson index: $1/D$ where $D = \text{sum}(p_i^2)$ and p_i is the proportional index of each species i in a plot. The evenness variable is H/log(S) where H is the Shannon Index and S is the number of species in a plot. $H = - \text{sum}(p_i * \ln(p_i))$. We calculate each metric using all the vegan package in R (*42*).

In this dataset, we started with 1291 potential plot-year observations but had to drop 2 plot-year observations because of missing richness values and another 58 observations because of missing productivity values.

S3b. Data transformations

Prior to estimating the effect of diversity on productivity, we transform our productivity variable (live biomass) and our diversity variables (richness, evenness, Simpson's index) by taking the natural logarithm of each plot-level measure. This transformation has several

advantages, which are all related in a statistical sense. First, both productivity and richness are strictly positive variables that exhibit right-skewed distributions (see Figure S1). Transforming by the natural logarithm reduces the skew of these variables, improving statistical efficiency (i.e., improves the precision of our estimates). Second, in an ecological sense, it is reasonable to assume that going from 2 to 4 species will on average have a bigger effect on productivity than going from 18 to 20 species but may have a similar proportional effect on average as a change from 10 to 20 species would have. In other words, the natural logarithm transformation makes sense in situations when it is better to compare relative changes rather than absolute changes. In other words, instead of assuming that P increases as a constant function of *R*, we assume that *P*

Figure S1. Plot-level (a & b) and site-level (c & d) species richness and productivity (above-ground live mass) between 2007-2017. (a) shows the levels of richness and productivity in all plots and (b) shows the log of richness and productivity in all plots. For comparison, (c) shows the average levels of richness and productivity across the 43 sites (see Table S1) and (d) the log of richness and productivity across the same sites.

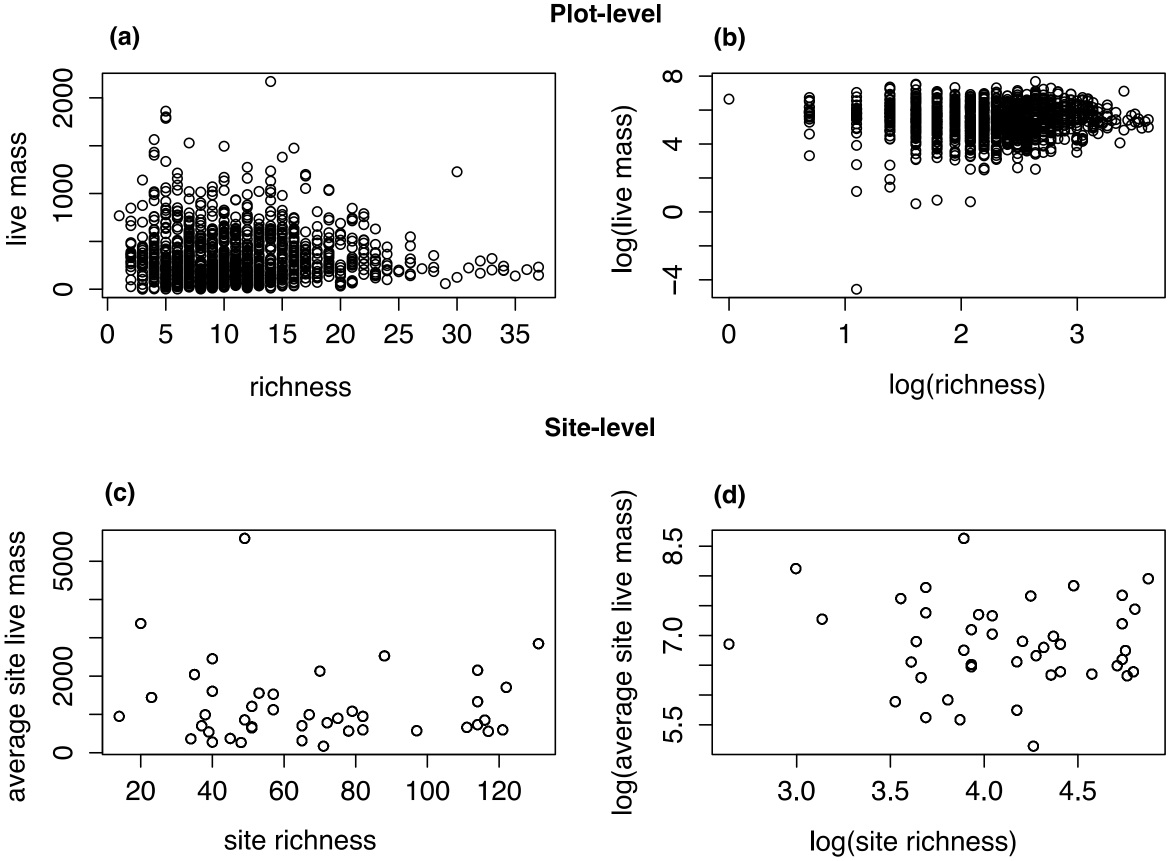

increases as a relative function to the current level of *P* as a function of *R*. Another way to say the same thing is that in a graph with richness on the horizontal axis and productivity on vertical axis, a straight line will not the best description of the relationship. Third, the coefficient on richness in our log-log specification has a well-define interpretation, which is a valuable trait; for most readers, a single coefficient is more accessible and easier to evaluate than a non-linear surface. In this SI (section S6), we also present the estimated effects of richness on productivity in levels (i.e., no transformation), including quadratic and cubic specifications that permit the estimated relationship to be non-linear.

Figure S2. **Plot-level changes in species richness through time**. This figure shows the change in plot-level species richness from year to year at each study site from between 2007-2017. All plots in the analysis have at least 5 years of data.

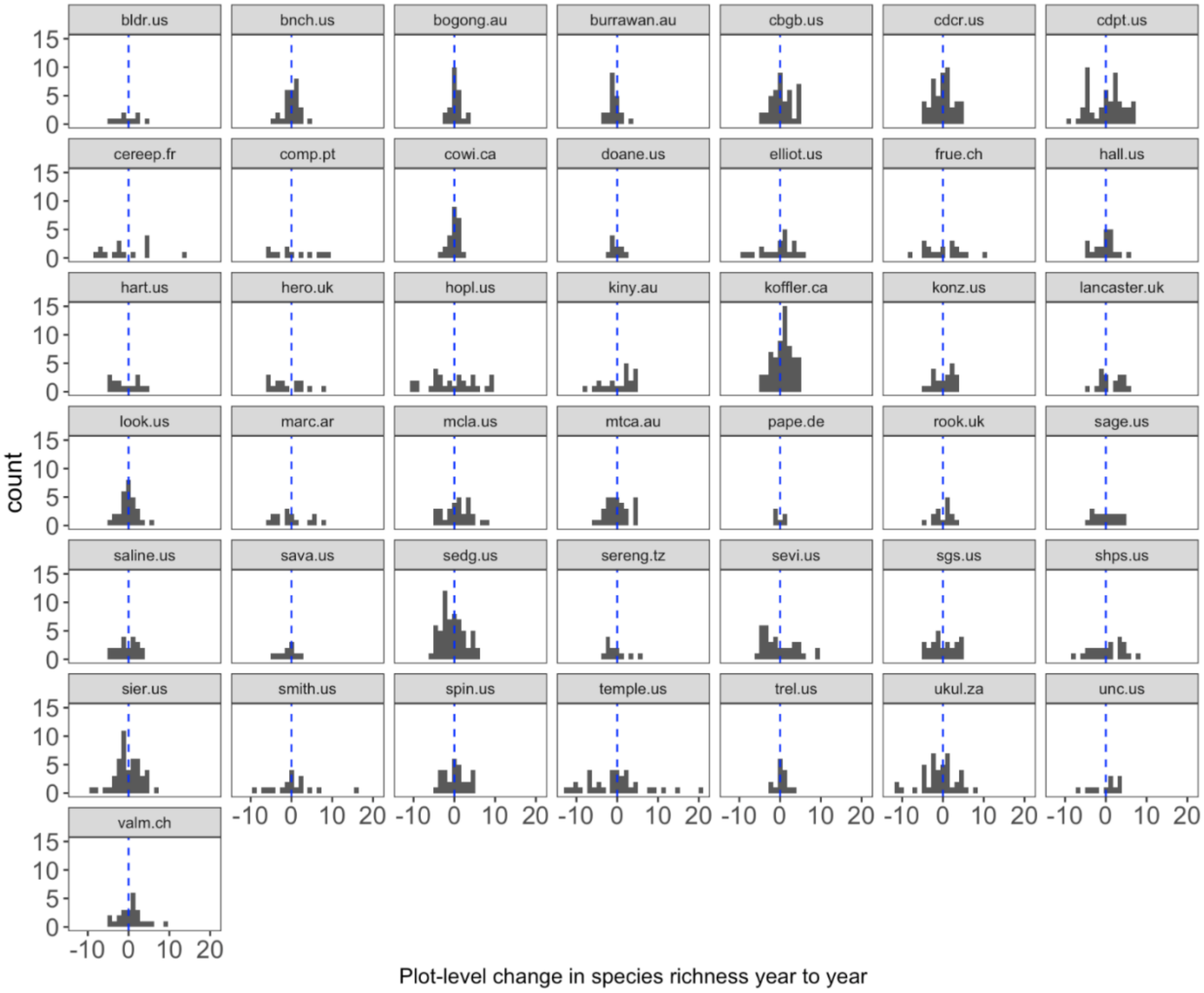

S4. Supplementary Methods: Main Design Estimator

S4a. Review of Main Design from Methods section

We present the details on the Main Design in the main text *Methods* section. Here, we elaborate on the estimation procedure used to implement the regression model for the Main Design. In our study design, an observation comes from a plot p located within a site *s* in a year *t*. Recall that, to eliminate the confounding effects of time-invariant plot attributes (δ_{ps}) and time-varying site attributes (μ_{st}), we estimate an equation of the following form:

$$\ln LiveMass_{pst} = \beta \ln Richness_{pst} + \delta_{ps} + \mu_{st} + \varepsilon_{pst} \qquad \text{(S1)}$$

Given that we have a ln-ln specification, β can be interpreted as an elasticity: the expected percent change in productivity given a one percent change in richness. In the economics literature, the time-invariant plot attributes (δ_{ps}) would be called "plot-level fixed effects." Note that fixed effects have a different meaning in economics than in ecology (see S1 Glossary). In economics, including δ_{ps} is said to control for "unobserved heterogeneity" across plots that can be a potential source of bias. Note that δ_{ps} is not part of the error term, as it would be in mixed (multi-level) models (see *Section S7*). Rather, it is a parameter to be estimated, just like β (i.e., β and δ_{ps} are assumed to be fixed and estimable, rather than assumed to follow a distribution). Time-invariant site attributes are not explicitly included in the equation because they are subsumed into the time-invariant plot attributes (i.e., plots are nested within sites and so fixed site attributes are controlled via fixed plot attributes).

The time-varying site attributes (μ_{st}) are modeled in a fully flexible way that allows a year-specific effect for each site (in the estimation, an indicator for each year is interacted with an indicator for each site). Explicitly estimating μ_{st} flexibly controls for confounding variation due to conditions at a site that vary from year to year, namely weather (e.g., temperature, precipitation), drought events, grazing, surrounding management, or other site-level attributes that change through time. In other words, this variable captures all year-specific conditions experienced by every plot at a given site.

The term ε_{pst} is a time-varying random error term at the plot level, assumed to have mean zero and no correlation with ln *Richness*, i.e., it corresponds to I_{pst} in Figure 1B. Errors at a given plot (ε_{pst}) may be serially correlated (i.e., temporally dependent even after conditioning on

richness and site-by-year effects), and thus we cluster the standard errors at the plot level (*43*). Our clustered estimation of the variance allows for arbitrary serial correlation within each plot, as well as heteroskedasticity across plots (*17*, *44*)). See our project page for code. Errors at a given site may also be correlated (even after conditioning on site-by-year effects) and thus, as a robustness check, we also estimate standard errors clustered at the site level (Table S2).

To sum up our design, we are asserting that, after controlling for time-invariant plot attributes that are correlated with richness and productivity, and time-varying site attributes that are correlated with richness and productivity, the remaining variation in richness in a plot is "as if randomly assigned," independently across time. In other words, the remaining variation in richness is driven by variables that have no link to productivity other than through their effect on richness (i.e., Z_{pst} in Fig. 1). If our assumption is correct, we can give a causal interpretation to the estimate of β. In section S7, we describe how we explore the sensitivity of our causal interpretation to violations of this assumption. The estimated effect of richness on productivity is reported in Figure 2 and Table S2.

As we noted in the Methods section of the main text, we seek to estimate the average causal response of an incremental change in R across all plots (i.e., the average effect across all possible one-unit changes). Recently, scholars have identified a potential form of misspecification bias that may arise when using models like Equation (S1) to estimate this average causal response when treatments are multi-valued and time-varying and the average causal response is heterogeneous across time or treatment values (*45*). Specifically, the regression estimator applies weights to all of the richness contrasts and year contrasts in the data and these weights can, in some rare cases, be negative. In the presence of heterogeneous average causal responses, such weights could overweight or underweight specific contrasts in a way that would create bias. This bias can arise when (1) the average causal response wanes or matures over the panel when treatment values change and remain at their new value across years (i.e., when they move to an absorbing state); and (2) the distribution of treatment values is highly non-normal and the average causal responses at extreme values of richness differ from the average causal responses for values in the middle of the distribution of richness values. There is no theoretical basis for the first source and the richness values in our panel data are well approximated by a normal distribution. Thus, we do not believe that this form of specification bias is a potential problem in our Main Design.

S4b. Estimation procedure and implementation

To ensure transparent, reproducible results among a wide range of scientists, we estimated Equation (S1) in two software programs and by multiple coauthors for reproducibility. We used the "reghdfe" command in STATA (v.16) (the "xtreg, fe" command yields the same estimates) and the "feols" command in R in the fixest package (v. 0.8.2). While there are other packages in R to execute this estimator, e.g., using the "felm" command in "lfe" (v 2.8-5) (*46*), we opt to use "fixest" because the standard error estimation matches STATA and yields a more conservative estimate based on the finite sample degrees of freedom correction for multi-way clusters. There is no consensus on the "correct" finite sample degrees of freedom correction for these models, so we opted for the more conservative option that results in larger standard errors.

S4c. Brief comparison of our design and aims to other study designs and aims

In our Main Design, our notion of causality and our approach differ from predictive (best-fitting) modeling approaches that use time-series data, such as convergent cross mapping designs (*47*). Our "intervention-based" notion of causality (*9*, *48*, *49*) is what experimentalists have in mind when they make causal claims (*50*). Furthermore, our model is not intended to be the best *predictiv*e model of productivity;[1] i.e., the best model for predicting the level of productivity in plots outside of our sample (*51*). In fact, the best predictive model of productivity may not even include richness as a variable. However, we are not interested in out-of-sample prediction of plot-level productivity. In contrast, our goal is to infer the causal effect of richness on productivity. But if one wanted to do such prediction, our design would pose challenges because our estimates are conditional on the sample; the plot-level fixed effects are not assumed to have a distribution (like they would in a mixed model), but rather are instead treated as fixed and estimable.

The approach in our Main Design also differs from a mixed-effect modeling approach more common in Ecology (*16*). While ecologists who are familiar with multi-level modeling may wonder why, given our data are comprised of plots nested within sites and annual observations nested within plots, we do not use this multi-level modeling approach as our Main Design. We opt to use our design, rather than a mixed effect model, because our approach makes weaker and

[1] This post by Paul Allison (2014) explains key differences in evaluating multivariate regression models for the aim of prediction versus causal inference: https://statisticalhorizons.com/prediction-vs-causation-in-regression-analysis

more plausible assumptions for our data context and question, compared to a mixed effect model. A full explanation is beyond the scope of this paper, but the main reason has two parts, which is laid out in more detail in Section S7. First, without more variable transformations, the multi-level modeling approach does not easily lend itself to controlling for as many unobservable sources of confounding as can be done in our estimator. Second, clustering our standard errors at the plot provides the same benefits that multi-level modeling does when estimating the variance-covariance matrix in the presence of intra-site correlations among plots (*44*). For more detail and discussion, see *Section S7. Comparison of Main Design to Common Design in Ecology* (a.k.a., multi-level modeling, hierarchical modeling, random effects modeling, mixed effects modeling, or variance components modeling).

Table S2. **Supplementary results on different variations of the main design.** Column (1) presents the results presented in the main text for the Main Design. The negative effect of ln of species richness on ln of productivity holds when clustering standard errors at the site level (column 2), when controlling for species evenness (column 3 & 6), and when using other measures of biodiversity (Simpson's Diversity – column 4) as well as the lagged effect of species richness in the prior year (ln $SpeciesRichness_{t-1}$) (columns 5 & 6). The estimated effect in column (1) is plotted in Figure 2.

Model with *ln*(live biomass) as outcome:	**(1)**	**(2)**	**(3)**	**(4)**	**(5)**	**(6)**
ln(SR)	**-0.2418 *****	**-0.2418 ****	**-0.2237*****		**-0.2185****	**-0.2057****
	(0.0854)	(0.0892)	(0.0851)		(0.0939)	(0.0948)
	[-0.40902; -0.0743]	[-0.4165; -0.0670]	[-0.3905; -0.0568]		[-0.4024; -0.0345]	[-0.3914; -0.0199]
ihs(Evenness)			-0.1864			-0.1450
			(0.2122)			(0.2387)
			[-0.6022; 0.2294]			[-0.6128; 0.3228]
ln(Simpson)				**-0.1701 ****		
				(0.0679)		
				[-0.3031; -0.0370]		
ln(lagged SR_{t-1})					-0.0146	-0.0096
					(0.0905)	(0.0903)
					[-0.1919; 0.1627]	[-0.1866; 0.1675]
Num. obs.	1231	1231	1231	1231	1093	1093
Num. plots	151	151	151	151	151	151
R^2 (full model)	0.87	0.87	0.87	0.87	0.87	0.87

S*ignif. Codes*: ***: 0.01, **: 0.05, *:0.1. *95% CI are shown in bracket.*
Robust Standard errors in parentheses, clustered at plot level in column 1 and clustered at site level in column 2.

S5. Supplementary Methods: Extensions of the Main Design Estimator

In this section, we present supplementary results, including variations in the specification of our Main Design shown in Equation S1 and Equation 2 in the main text.

S5a. Species evenness

The negative estimated effect of richness on productivity (Figure 2) could reflect changes in evenness, which may covary with changes in richness. In our sample, species richness varies a lot over time within plots, but evenness does not (Figure S3). Thus, we do not suspect that failing to include species evenness in Equation (S1) is a source of bias, but we nevertheless re-estimate the equation after adding a measure of evenness. We transform the evenness variable with an inverse hyperbolic sine (IHS) transformation, which has an effect similar to the natural logarithm transformation but, unlike the natural logarithm transformation, is appropriate for variables, like changes in evenness, that have many zero values (the natural logarithm of zero is undefined; (*52*)).

After accounting for evenness, the negative relationship between richness and productivity remains unchanged (Table S2), implying that the estimated effect of species richness in the first column comes from changes in species richness rather than evenness. The estimated coefficient on evenness is imprecisely estimated (Table S2) (i.e., large standard errors). Thus, anyone interested in estimating its causal effect on productivity would not be able to draw precise inferences from our data. This imprecision highlights that the strength of our design – its ability to leverage change in diversity over time within plots to isolate the causal effect of diversity – can be a weakness when the variable of interest does not change much over time within plots. If this lack of temporal variation is common in many non-experimental contexts, experimental designs varying evenness (e.g. (*53*)) may be the only way to obtain precise estimates of the role of evenness on productivity or other ecosystem functions.

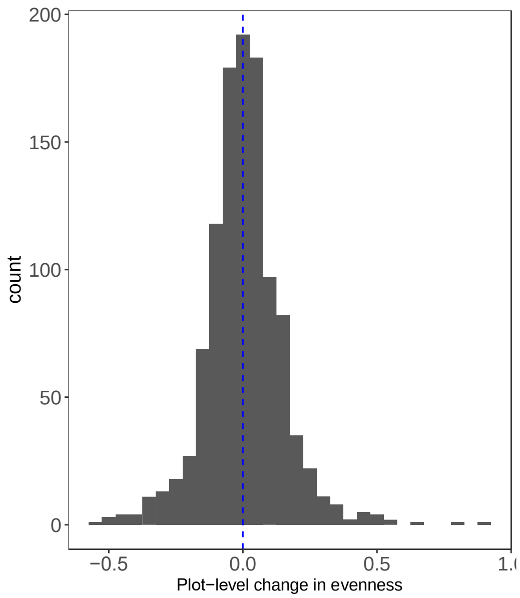

Figure S3. Year-to-year variation in species evenness per plot for the dataset described in Table S1.

S5b. Functional form

The effect of species richness on productivity could vary by the magnitude of the change in the number of species. To detect this non-linearity, we estimate a quadratic specification of our equation in which the variables are not log transformed (Table S3: ‘Quadratic’ column). We detect evidence that the negative effect of richness on productivity becomes smaller as richness increases, i.e., the coefficient on the non-squared term is negative and the coefficient on the squared term is positive. Species richness ranges from 1 to 37 species in our dataset, which determines the range over which there is a positive or negative effect. With the quadratic specification, we find that the estimated effect or richness on productivity only turns positive in plots over 31 species, which represents only 14 observations and 1.14% of the data (see Table S3 – ‘Quadratic’ column). For completeness, we also present estimates with the linear specification and untransformed variables (level-level) and estimates with only productivity log-transformed (log-level) (Table S3). We also estimated a cubic specification, and the estimated cubic term was quantitatively and statistically indistinguishable from zero (see code on our project page, DOI/10.5281/zenodo.7675340).

Table S3. Estimates of the effect of species richness on productivity *P* under changes in model specification for the functional form of this relationship. The columns compare the estimates from main log-log model (in column 1) to estimates from models with (2) the *ln* of productivity *P* but untransformed richness (i.e., richness in levels), (3) untransformed richness and productivity *P*, and (4) untransformed richness and productivity *P* with a quadratic term for richness. Standard errors, clustered at plot level, are in parentheses. 95% CIs are in brackets. All models include plot and site-by-year fixed effects as in Equation (S1).

	(1) ln(*P*)	(2) ln(*P*)	(3) *P*	(4) *P* (*Quadratic*)
ln(Richness)	-0.2418***			
	(0.0854)			
	[-0.4092, -0.0743]			
Richness		-0.0147*	-1.916	-16.83**
		(0.0080)	(2.919)	(6.623)
		[-0.0304, 0.0009]	[-7.637, 3.805]	[-29.81, -3.850]
Richness^2				0.5602**
				(0.2162)
				[0.1364, 0.9840]
Num. obs.	1231	1231	1231	1231
Num. plots	151	151	151	151
R^2 (full model)	0.867	0.865	0.83	0.83

S*ignif. Codes*: ***: 0.01, **: 0.05, *:0.1

S5c. Moderating effect of site-level species richness

We tested the hypothesis that site-level richness moderates the effect of plot level richness on productivity. This hypothesis is motivated by the observation that one potential reason for the observed negative effect of plot richness on plot-level productivity is that the “best performing” species enter a plot and win by becoming more productive (*54*) and thus causes a decline in richness to lead to an increase in productivity. This relies on having a large pool of species at a site that can colonize to take over during specific years.

We found no evidence supporting the hypothesis that the effect of plot-level species richness on plot-level live biomass depended on the levels of the observed site richness across all years or per year, nor on the numbers of introduced and native species at the site level (Table S4). We recommend that future research could test this hypotheses using data that includes direct observation of dispersal patterns (e.g., (*54*)), which are not available for this dataset at present.

Table S4. **No dependence of the plot-level species richness (SR) effect on site-level species richness (site SR) characteristics on productivity.** All estimates are on a ln-ln scale. We consider several site-level species richness measures, including: site-level richness across all years (Site SR): count of all unique taxa ever observed across all plots in all years at that site), the site-level richness per year (Site SR per year): count of unique taxa observed across all plots at the site in that year), the count of all unique introduced taxa at the site (Site Introduced SR) and the count of all unique native taxa at the site (Site Native SR). Interactions are indicated by a "x." If anything, we find evidence that controlling for site level richness variables makes the estimated effect of the log of plot species richness on log productivity more negative. To see 95% confidence intervals as well, see the project page (TableS4_R_CI.tex).

	**Model:**			
	Total Site SR	Site Introduced SR	Site SR by year	Site Native SR
ln (SR)	-0.4398 **	-0.2753**	-0.3834 *	-0.3184**
	(0.2155)	(0.1197)	(0.1998)	(0.1494)
ln(SR) x Site SR	0.0026			
	(0.0027)			
ln(SR) x Site Introduced SR		0.0018		
		(0.0061)		
ln(SR) x Site SR per year			0.0041	
			(0.0050)	
ln(SR) x Site Native SR				0.0021
				(0.0031)
Num. obs.	1231	1231	1231	1231
Num. plots	151	151	151	151
R^2 (full model)	0.87	0.87	0.87	0.87

Signif. Codes: ***: 0.01, **: 0.05, *:0.1
Robust Standard errors in parentheses (clustered at plot level).

S5d. Moderating effect of site-level productivity

A recent study by Wang et al. (*55*) found that the effect of biodiversity on productivity was moderated by the average level of productivity at a site, meaning that effect of biodiversity on productivity differed between high versus low productivity sites. In response, we test whether our results are altered by considering site-level productivity as a moderator. We consider site-level productivity in four ways: two using continuous variables and two using the cut-offs for high, medium, and low productivity classifications from Wang et al (*55*). Wang et al (*55*), however, used cross-sectional analyses to estimate this effect. Here, we can measure site-level

productivity in two ways: average over the entire time series (‘Average prod. per site’), and site-level productivity per year (‘Average Prod per site & year’). In all calculations of site-level productivity, we include the average productivity for the unmanipulated (control) plots, but not the experimental plots at the Nutrient Network experimental sites.

We expand and estimate our main model in (equation S1) adding an interaction term between ln $Richness_{pst}$ and *Avg Productivity* (Table S5). Next, Wang et al (*55*) categorize sites as high, medium, or low productivity in the 151 grids in HerbDivNet data based on mean productivity in a grid: low between 30.18-238.73 (g/m^2), medium between 239.67-409.69 (g/m^2), and high between 414.29-1382.42 (g/m^2). Our productivity, in terms of live aboveground biomass at the site average across years, ranged from 62.48 to 1124.27 g/m^2; whereas the average site-level productivity per year ranged from 5.372 to 1609 g/m^2. Thus, to be comparable to Wang et al (*55*), our groups were classified as: low below 239.67, high over 414.29, and the rest of sites as medium productivity. We adopt these cut-offs and rerun the models interacting the ln $Richness_{pst}$ with the *Productivity_Group* (see Table S5 for details).

Table S5. Estimating the moderating effect of site-level productivity on the effect of plot-level species richness (SR) on productivity, using continuous measures of productivity. As moderators, we consider the average site-level productivity per year (column 1) and the average site-level productivity across years (column 2). Interactions with plot-level species richness per year are indicated by a “x.” To see 95% confidence intervals as well, see the project page (TableS5_R_CI.tex). All estimates are on a ln-ln scale.

	**Model:** (1)	(2)
ln(SR)	**-0.3305 ****	**-0.3532****
	**(0.1609)**	**(0.1635)**
ln(SR) x Ave. Site Prod. Per Yr.	0.0003	
	(0.0004)	
ln(SR) x Ave. Site Prod.		0.0004
		(0.0004)
Num. obs.	1231	1231
Num. plots	151	151
R^2 (full model)	0.87	0.87

S*ignif. Codes*: ***: 0.01, **: 0.05, *:0.1
Robust Standard errors in parentheses (clustered at plot level).

Across these analyses, we can detect no moderating effect of site-level productivity, based on estimated coefficients and their SEs in Table S5 and S6. See R code to reproduce analyses at the project page.

Table S6. **Estimating the moderating effect of site-level productivity on the effect of plot-level species richness (SR) on productivity, using categorical groups of high, medium, low productivity based on Wang et al.** We interact plot-level richness with each productivity group; interactions are indicated with an 'x' in the results table. Productivity groups were determined as follows. Wang et al (*55*) use a single year of data; to mirror this measure, we use an average productivity per site across years (column 2). In contrast to Wang et al (*55*), we also interact plot-level richness with an average productivity per site per year (column 1). To see 95% confidence intervals as well, see the project page (TableS6_R_CI.tex). All estimates are on a ln-ln scale.

`	(1)<br>Average Prod. per site & year	(2)<br>Average Prod. per site
ln(SR)	**-0.2090***	**-0.1946**
	**(0.1070)**	**(0.1395)**
ln(SR)x ProdGroupMedium	0.1075	
	(0.1155)	
ln(SR) x ProdGroupHigh	-0.0718	
	(0.1740)	
ln(SR) x ProdGroup:WangCutoffsMedium		-0.1358
		(0.1985)
ln(SR) x ProdGroup:WangCutoffsHigh		0.0623
		(0.2123)
Num. obs.	1214	1231
R^2 (full model)	0.77	0.77
Num. plots	151	151

S*ignif. Codes*: ***: 0.01, **: 0.05, *:0.1
Robust Standard errors in parentheses (clustered at plot level).

S6. Supplementary Methods: Robustness Checks to Assess Potential Threats to Internal Validity

In our Main Design (*Section S4*), the key, untestable assumption for drawing a causal inference from our estimator is that, after controlling for time-invariant plot confounders and time-varying site confounders, the remaining variation in richness in a plot is "as if randomly assigned," independently across time. In the main text (Figure 3), we consider potential violations of this assumption and the implications for our inferences. More specifically, we conduct a series of analyses that rely on alternative assumptions for causal inference. As noted in the main text, the results are consistent across all approaches. Here, we describe these approaches in more detail.

First, we explore potential violations in our assumption that the effect we are estimating goes from richness to productivity, and not the other way around (in *Section 6a*). Because species richness and biomass measures are taken simultaneously each year, as they typically are in many ecological data sets, we cannot rely on temporal sequencing of the data to rule out reverse causality. To address this potential threat to causal inference in our design, and in the process also address potential bias from unobserved, time-varying plot attributes, we take two approaches: (a) we posit a mechanism through which productivity affects richness – *i.e.,* shading (based on (*56*)) – and then block this mechanism and evaluate the change in our estimated effect of richness on productivity (*Section 6a.i*); and (b) as an alternative to our main estimator (Equation S1), we use an estimator that can estimate the effect of richness on biomass for a subsample of the observations for which we can more credibly argue that the direction of causality goes from richness to productivity (*Section 6a.ii*).

After assessing the potential threat to inference from reverse causality, we then explore potential violations in our assumption that there are no time-varying plot attributes that are systematically correlated with richness and productivity. To do this, we take two approaches. First, we explore violations in our assumption that prior productivity does not influence current richness, an effect that could be mediated by prior species richness (i.e., reverse causality in prior year) or by other dynamic mediators. To address this potential source of bias from a plot-level, time-varying confounder, we use two alternative estimators (*Section S7b.i*) that replace the plot "fixed effects" with lagged productivity (i.e., lagged dependent variable estimators). Second, we take a more general approach to quantifying our uncertainty about the potential bias from a time-

varying, plot-level confounders. We create bounds our estimated targeted causal effect by assuming that there are time-varying plot attributes that are systematically correlated with richness and productivity. Specifically, we explore how our estimated effect would change if there were an unobserved confounder that was negatively correlated with richness and positively correlated with productivity (i.e., a source of bias that yields a spurious negative causal relationship between richness and productivity in our design; *Section S7b.ii*).

S6a. Reverse causality: productivity causes species richness

For changes in richness to cause changes in productivity, changes in richness must occur prior to changes in productivity. However, as in most experimental and observational studies on the relationship between diversity and productivity, the Nutrient Network data on diversity and productivity are collected at the same time each year. In the absence of high-resolution temporal data (e.g., daily), we must make additional assumptions and run additional tests to rule out reverse causality. If productivity were to negatively affect richness, that causal relationship could mask a positive relationship of R on P in our design. In other words, our estimated coefficient of β in Equation (S1) may reflect a causal relationship that runs from productivity to richness, rather than the other way around (i.e., it reflects a causal graph with a directed edge that flows from P to R instead from R to P).

To illustrate the problem caused by reverse causality, we create a new causal graph in Figure S4 (this graph is not acyclic because it has bi-directional arrows between two variables). We use the notation from Equation (S1) but suppress the plot subscript p and add a subscript $t\text{-}1$ for time lagged one period. In this new graph, we assume that bias from regressing P_t on R_t is not coming from unobserved confounders, but rather from simultaneous causal relationships in which P_t and R_t directly cause one another. If we regress P_t on R_t and the estimated coefficient β is less than 0, a critic of our analysis could argue that the true β is greater than zero but masked because $\alpha < 0$ and $|\alpha| > |\beta|$. If P_t affects R_t, ε_t is necessarily correlated with R_t, which is a violation of our assumption that, after controlling for time-invariant plot attributes and time-varying site attributes, the remaining variation in richness in a plot is "as if randomly assigned," independently across time (note: a similar violation arises if P_{t-1} affects R_t, whereby ε_{t-1} is necessarily correlated with R_t; we address that possibility in the next section).

The theoretical and empirical literature on the causal effect of productivity on richness does not have a clear conclusion: studies report productivity has zero effect on richness, a negative effect on richness, and a humped-shaped effect. Nevertheless, there are some studies that report detecting a negative effect of productivity on richness (e.g., (*56*)). To address this potential threat to the internal validity of our estimated negative effect of richness on productivity, we take two approaches: (a) we block a mechanism (M_t in Figure S4) through which productivity can affect richness; and (b) we find a variable has no effect on productivity other than through its effect on richness (Z_t in Figure S4) and use this instrumental variable to create an unbiased estimator of the effect of richness on productivity.

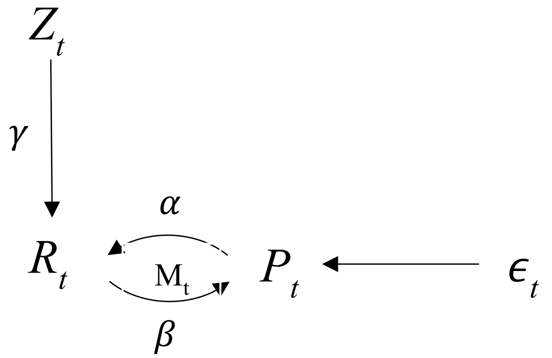

Figure S4. Reverse Causality in the Richness-Productivity Relationship. In this causal graph, richness in one period (*R*) has a causal effect on productivity (*P*) in the same period, and vice-versa. The variable *M* represents mechanisms that mediate these causal effects, which can be different depending on direction of the causal arrow. Richness in the prior period (R_{t-1}) affects richness in the current period (R_t). ε are time-varying factors that affect productivity, which, in our estimation, we assume can be correlated across time. Z_t is often called an "instrumental variable."

S6a.i. Blocked mechanism design

In regression analyses of a causal variable, it is well known that if one conditions on a mechanism variable, the estimated coefficient on the causal variable will no longer include the effect of the mechanism variable. In our grassland sample, if productivity were to negatively affect richness, we assume that this effect is, in part, mediated by shading; i.e., more productive plots generate greater shade, which in turn reduces richness (*56*). If the estimated negative relationship between richness and productivity in Figure 2 were an artifact of reverse causality mediated by shading, then putting our shading variable in Equation (S1) as a covariate would

block the effect of productivity on richness and the sign of the coefficient on richness (β) would become positive (or small and statistically insignificant if the true relationship between richness and productivity were zero). See Figure S5 below. If shading is not an important mechanism through which productivity would affect richness in our sample, or if our measure of shading is a poor measure of the shading mechanism, our mechanism-blocking design would fail to quantify the potential threat of reverse causality. Indeed, productivity could alter biodiversity through non-light pathways, such as soil resource use, but this effect of productivity on richness is expected to, at least in part, be mediated by reductions in light from increased biomass that, in turn, reduces richness in a plot. Thus, if reverse causality was a substantial threat to our identification strategy for the effect of richness on productivity, we would expect the coefficient of richness on productivity to shift towards more positive values.

As an estimate of shading, we measure the fraction of photosynthetically active radiation (e.g., light used by plants) that reaches the soil. This measure is calculated as the ratio of photosynthetically active radiation recorded below the plant canopy (ground level, mean of two readings) and that measured above the canopy. Measurements are carried out using a light meter (e.g. Ceptometer) at the same time and in the same 1m^2 sub-plots used for vegetation cover estimates. We have annual measures of ground-level light for 145 plots of our 151 plots (1011 of our 1231 observations).

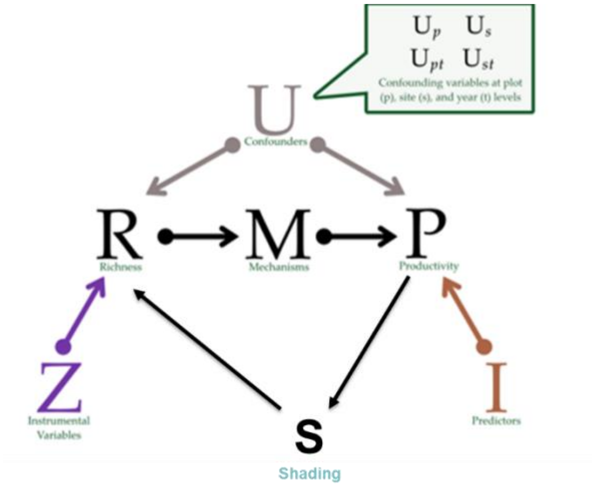

Figure S5. Productivity affects species richness via shading as a mechanism. In this causal graph, in addition to changes in richness causing changes in productivity, changes in productivity change causes changes in richness via shading (*s*). Other mechanisms (*M*) may also be operative but are not explicitly included in the graph.

First, we confirm that the estimated effect of richness on productivity does not change when we use the subsample of 1,007 observations for which we have measures of shading. It does not: a 10% increase in richness leads to an estimated 2.6% decrease in productivity, 95% CI [-4.4%, -0.8%] (see project page DOI/10.5281/zenodo.7675340). Next, we re-estimate Equation (S1) with our shading variable included. The estimated negative effect of richness on productivity does not change: a 10% increase in richness leads to an estimated 2.6% decrease in productivity, 95% CI [-4.4%, -0.8%].[2] Said another way, if reverse causality was a substantial threat to our identification strategy for the effect of richness on productivity, we would expect that, after adding shading to Equation (S1), the coefficient of richness on productivity would become substantially smaller in absolute value or, possibly, to become positive. Yet the estimate remains unchanged.

S6a.ii. Instrumental variable design

As an alternative approach to assess the potential threat of reverse causality that makes *different* assumptions from the mechanism blocking analysis, we adopt another statistical approach that is common in economics and public health, but rare in ecology: an instrumental variable design (*21*, *22*, *57–59*).

We seek an attribute of the system that has a relationship with richness, but, after conditioning on other attributes, has no relationship with productivity other than through its relationship with richness. Such an attribute is illustrated by *Z* in Figures 1, S4 and S5. In economics and biostatistics, *Z* is called an instrumental variable (IV) or a surrogate variable. An example of a potential IV is randomization of planted richness by an experimenter. In field experiments, randomization of richness helps isolate the causal effect of richness on productivity, but only when the randomization affects productivity in a plot solely through its effect on richness, an assumption called excludability (*60*) or the exclusion restriction (i.e., one must assume there is no arrow going from *Z* directly to *P*).

In the absence of randomization, one must use theory and experience to identify a naturally occurring IV. Each of the plots in our sample are unmanipulated plots that are embedded in blocks of manipulated plots in the Nutrient Network. In other words, each unmanipulated plot in

[2] See the Github project page output for Table_MechBlocking_R_se.tex and Table_MechBlocking_R_ci.tex for more details.

our sample is surrounded by a set of plots with experimental nutrient additions (see (*61*)). These manipulated experimental plots received randomized amounts of nutrient additions, which subsequently affected the experimental plots' richness (*62*). We assume that the experimentally manipulated richness in these plots can affect the richness in unmanipulated plots in the same block through ecological dispersal channels but does not affect the productivity of these unmanipulated plots except through the effect on the plots' richness (an assumption made more plausible by the randomization of nutrients in the neighboring plots). If that assumption is correct, we can use the average richness of an unmanipulated plot's neighboring manipulated plots in the same block as an instrumental variable for richness in the unmanipulated plot.

This time-varying spillover effect from manipulated to unmanipulated plots is plausible if either (a) the spatial pattern of nutrient manipulations in the experimental plots around each unmanipulated plot varies across blocks, or (b) the spatial pattern of manipulated plots around each unmanipulated plot varies across blocks (i.e., variation in how far apart plots in a block are to each other or in their plot-level attributes that moderate the effect of nutrient addition n on richness). We lack digital maps of the experimental designs for every site that we could use to determine the exact distance between and spatial configuration of plots and empirically confirm either of these assumptions. However, colleagues who manage the Nutrient Network believe these assumptions are credible (Dr. Eric Seabloom, *personal communication*). Moreover, when we regress unmanipulated plot richness on the average richness of the manipulated neighboring plots in the block, we obtain a positive and statistically significant coefficient, which is consistent with the posited spillover effect (Table S7*, column 2*).

Table S7. Results from the Instrumental Variable Design

	(1)	(2)
	Second Stage of 2SLS (Outcome=Productivity)	First Stage of 2SLS (Outcome=Richness)
ln(Richness)	-0.24<br>(0.37)<br>[-0.96, 0.49]	
ln(Average Neighboring Plots Richness)		0.49<br>(0.12)<br>[0.26, 0.72]
Number of Plots	151	151
Number of Sites	43	43

Number of Observations	1212	1212
Montiel-Pflueger effective F-statistic		17.44

2SLS refers to a two-stage least squares estimator, with the results from the first stage (predicting richness) in column 2 and the results from the second stage (estimating effect of richness on productivity using the instrument from the first stage) in the column 1. The M-P effective F-statistics is used to test for a weak instrument (a test that is robust to heteroscedasticity, serial correlation, and clustering; (*63*)). The value of the M-P effective F-stat implies we can reject the null hypothesis of a weak instrument. Standard errors in parentheses (clustered at plot level) and 95% CI in brackets.

The excludability assumption implies that, after we condition on time-invariant plot attributes and time-varying site attributes, the richness of a plot's manipulated neighbors affects the plot's richness but has no effect on the plot's productivity other than via the effect on the plot's richness. In other words, the drivers that cause the average richness of an unmanipulated plot's neighbors to change over time only affect the unmanipulated plot's productivity through a change in the unmanipulated plot's richness. The manipulated plots are randomly manipulated (*32*) and these manipulations have been shown to affect species richness (*62*). Thus, some of the changes in richness in neighboring manipulated plots are being driven by exogenous factors that could plausibly be assumed to not affect unmanipulated plot productivity except through their effects on the unmanipulated plots' richness. For this assumption to be valid, we must assume that the nutrient additions that affect the neighboring plots' richness have no direct effect on an unmanipulated plot's productivity other than via effects on an unmanipulated plot's richness (e.g., rather than dispersal being the mechanism through which neighbor plot richness affects own plot richness, it could be the nitrogen applications leaching through the ground). Colleagues who manage the Nutrient Network believe this assumption is credible (Dr. Eric Seabloom, *personal communication*).

In addition to the excludability assumption, we need two other assumptions to use this IV to estimate a causal effect of richness on productivity: (1) first-stage non-zero effect of the IV; and (2) monotonicity. The first-stage non-zero assumption of the IV design requires that there be a correlation between neighbor's richness and own richness, on average – in other words, the effect of neighbor's richness on own richness is not zero for all plots. We can verify this assumption empirically (Table S8, column 1): after controlling for plot-level and site-level confounders, the average richness of the neighboring plots has a positive association with own plot richness. The monotonicity assumption implies that, for all plots, the relationship between a plot's neighbor richness and its own richness can only be in one direction: it is either ≥ 0 or ≤ 0. In other words, we assume that we could not observe that, for some plots, higher neighbor

richness increases own richness, but for other plots, higher neighbor richness decreases own richness. The monotonicity assumption is untestable. Yet given our ecological motivations for using neighboring richness as an instrumental variable, we believe a non-negative, monotonicity assumption is a valid approximation of the field reality, i.e., assuming that an increase in the richness of surrounding manipulated plots can never decrease an unmanipulated plot's richness.

In comparison to our main design (*Section S4*), the IV design has two disadvantages. First, because the IV design uses only variation in richness that comes from neighboring plot richness, it will tend to have lower statistical power. Second, the IV design increases internal validity at the potential expense of external validity. Rather than estimate the average effect on productivity from any change in richness, we estimate the average effect for a subset of the changes in richness. This subset is comprised of what are called "compliers" – plot-year observations for which the richness value would have been different had the average richness in surrounding plots been different. Given our instrumental-variable is multi-valued, there are many types of compliers (e.g., plots that had 5 species in 2007 that would have had 6 species had their neighboring plots had higher species richness). If the average causal effect of changes in richness that come from changes neighboring plot richness differs from the average causal effect of changes in richness that come from other attributes of the system, the generalizability of the inferences from the IV design is more limited than in our main design. Another way to view the more limited external validity of the IV design is that the IV design allows us to estimate the average effect of richness on productivity for changes in a plot's richness that are induced by changes in a neighbor's richness. If that average effect is not the same as the overall average effect, the estimates from the IV design and the main design may differ, even if there is no reverse causality or other forms of hidden bias in the main design.

In the IV design, we use two-stage least squares, linear, additive, fixed-effects estimator (*3*), which we implement with the "ivreghdfe" command in STATA v.16. The estimated effect of richness on productivity, as well as the first-stage estimates and F-test are reported in Table S8. To ensure reproducibility and use open-access software, we also estimate the IV equation in R using 'feols' in the fixest package (v. 0.8.2).

The estimate from the IV design implies a nearly identical estimate of the relationship between richness and productivity as we obtained from the main design: a 10% decrease in richness leads to a 2.4% increase in biomass. As expected, the effect is estimated imprecisely

(i.e., large confidence intervals). IV is a less efficient estimator (*12*), thus leading to predictably large confidence intervals.

Note on interference among plots: An unstated assumption in each design we implemented in our study – an assumption found even in randomized controlled experimental designs – is "no interference among units" (see (*64*)), which means that the potential productivity outcome in an unmanipulated plot at a specific level of richness is unaffected by the richness levels of other unmanipulated plots. The IV design may seem to imply that this assumption is violated, but the IV design relies on manipulated plot richness affecting unmanipulated plot richness, which is not interference in our study designs. In our designs, each unmanipulated plot is still assumed to have one potential outcome per richness level. We believe that interference is absent in our designs because, based on discussions with Nutrient Network coordinators, the unmanipulated plots are sufficiently separated from each other within each site in order to not interfere with each other. In other words, each unmanipulated plot's potential productivity outcomes under different richness values only depends on its richness value, and not on the richness values of other unmanipulated plots.

S6bi. Dynamic panel designs

In Figure S6, we present a more complicated causal graph than the DAG in Figure 1B. To make the new graph more compact, we do not specify whether variables are acting at the plot or site level, and we assume that all variables are measured in the current period, unless otherwise stated. In this new causal graph, we have productivity (P_t) and lagged productivity (P_{t-1}), we have common causes (U) of richness (R) and P_t and P_{t-1}, and common causes (I) of P_t and P_{t-1}. This causal graph is "unidentified" – in other words, there is no observational design that could estimate the effect of R on P without bias, unless we make more assumptions.

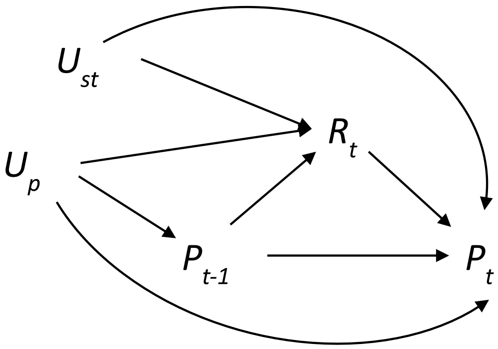

Figure S6. Reverse Causality in the Richness-Productivity Relationship in which Prior Productivity Affects Current Richness. In this causal graph, richness in the current period (R_t) has a causal effect on productivity (P_t) in the same period, and productivity in the prior period (P_{t-1}) has a causal effect on richness and productivity in the current period. The graph includes the two types of confounders that are the focus of the Main Design: time-invariant, plot-level confounders (U_p) and time-varying, site-level confounders (U_{st}). For simplicity, the graph does not include the variable *I* from Figure 1 (i.e., factors that affect *P* but not *R*).

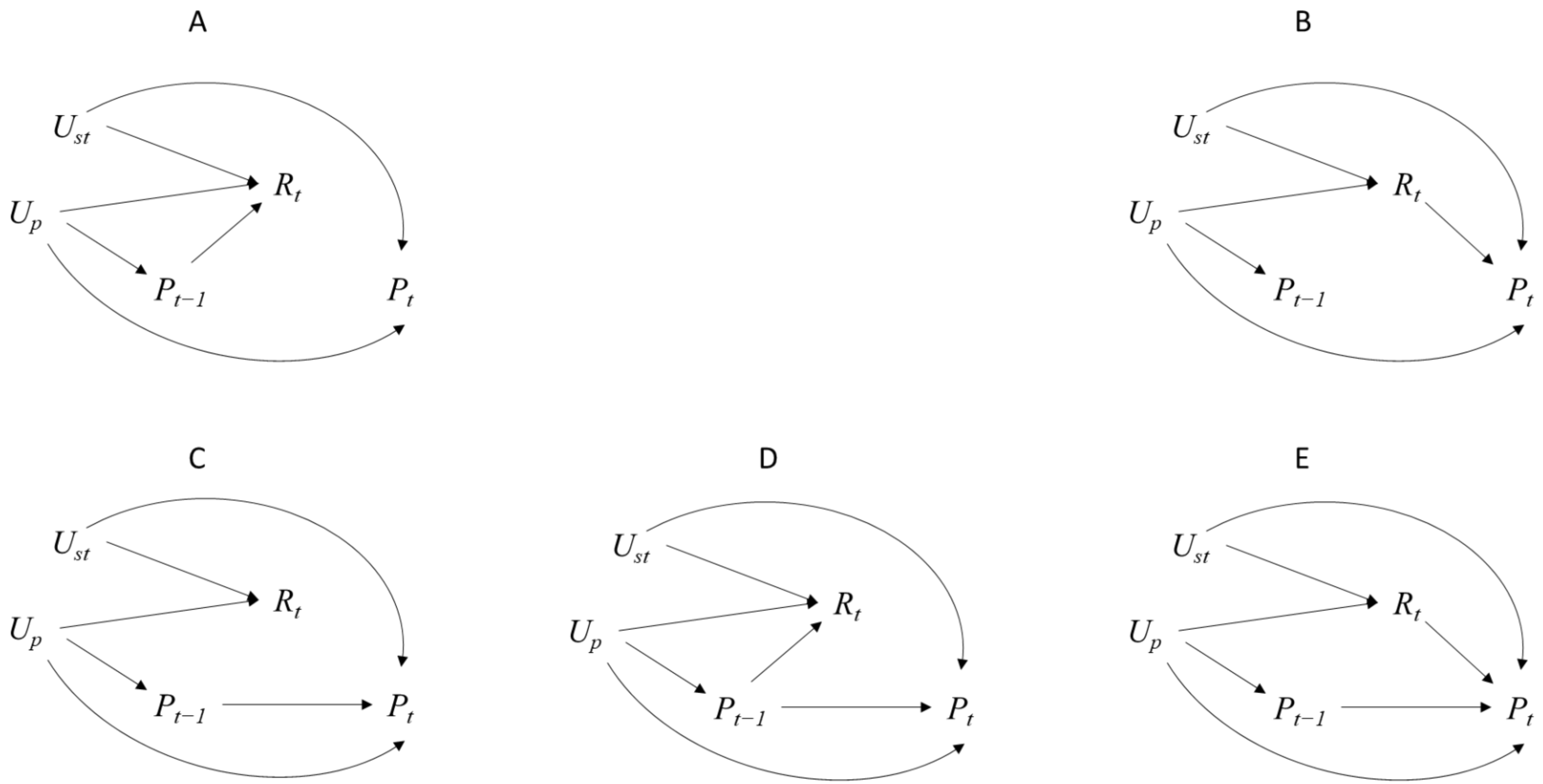

Figure S7. Causal Graphs that Reflect the Main Design Estimation Strategy. Our Main Design is valid for each causal graph in this figure. The graph includes the two types of confounders that are the focus of the Main Design: time-invariant, plot-level confounders (U_p) and time-varying, site-level confounders (U_{st}). For simplicity, the graph does not include the variable *I* from Figure 1 (i.e., factors that affect *P* but not *R*).

Our Main Design assumes the most important sources of confounding are from U_p and U_{st} and that a directed edge from P_{t-1} to R_t does not exist in combination with a directed edge from P_{t-1} to P_t (i.e., all panels in Figure S7 are allowed). Productivity can be serially correlated over time from unobserved variables, but there cannot be a causal effect of P_{t-1} on P_t that is mediated by R_t in this design.

If there were instead a directed edge from P_{t-1} to R, but either (i) no directed edge from U to R, P_{t-1} and P_t (panel A of Figure S8; i.e., no unobserved common causes of richness and productivity) or (ii) no directed edge from I to P_{t-1} and P_t (panel B of Fig. S8; i.e., no unobserved, persistent causes of productivity), one could estimate the effect of R on P without bias by conditioning on P_{t-1}; in other words, via a lagged-dependent variable specification (in this context we have both a form of reverse causation and a form of time-varying confounding).

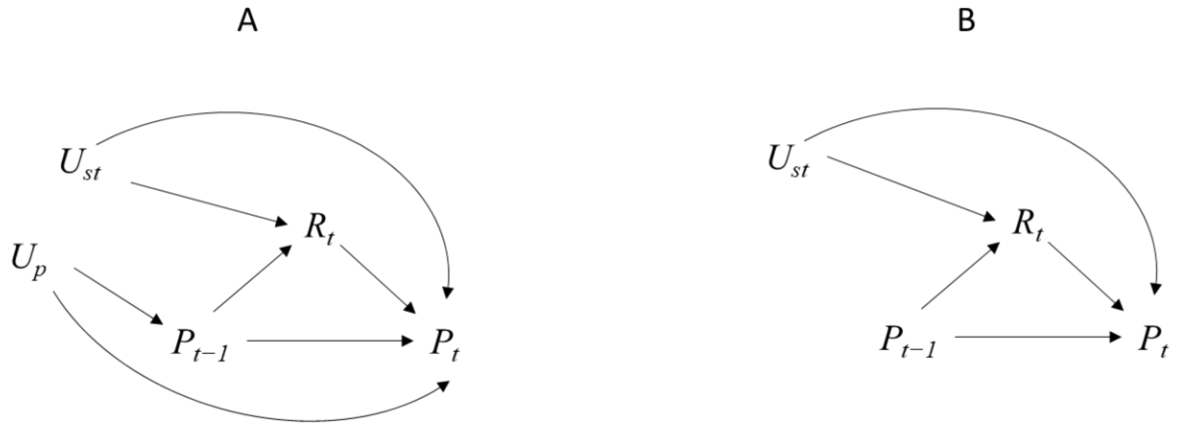

Figure S8. Reverse Causality in the Richness-Productivity Relationship in which Prior Productivity Affects Current Richness. In these causal graphs, richness in the current period (R) has a causal effect on productivity (*P*) in the same period, and productivity in the prior period has a causal effect on richness and productivity in the current period. The graph includes the two types of confounders that are the focus of the Main Design: time-invariant, plot-level confounders (U_p) and time-varying, site-level confounders (U_{st}). For simplicity, the graph does not include the variable *I* from Figure 1 (i.e., factors that affect *P* but not *R*).

The causal processes implied by the causal graphs in Figure S7 and S8 are observationally indistinguishable. The data alone cannot tell us which estimation strategy, our Main Design or a lagged-dependent variable specification, is appropriate (unless one is willing to make untestable, parametric assumptions). In other words, productivity is temporally correlated across time – we can see that correlation in the data. The source of that temporal dependence could be unobserved persistent causes of productivity (often called “unobserved heterogeneity” in social science and

biostatistics). The design used to generate the main estimate in Fig. 2 controls for these causes and thus eliminates any potential biases when such causes also are linked to richness. However, the source of temporal dependence could be a direct link between productivity in one year and productivity in the next year (e.g., via nutrient storage in roots). If that were the case, and if biomass in one year also affected richness in the next year (often called "*state dependence*" in social science and biostatistics), our main design may have bias. If, for example, productivity was positively correlated across years and lagged productivity had a negative effect on current richness, our estimated effect of richness on productivity using our main estimator would be too negative. To eliminate that bias, we could condition on lagged productivity:

$$\ln LiveMass_{pst} = \beta \ln Richness_{pst} + \Omega \ln LiveMass_{ps(t-1)} + \mu_{st} + \varepsilon_{pst} \quad \text{(S2)}$$

In other words, if Fig. S8 is the correct interpretation of the systems we are studying, and we use the estimator from Equation S1, the estimated β will be too large in the negative direction (away from zero) compared to the true effect. If, however, Fig S7 is the correct interpretation of the systems we are studying, and we use the lagged dependent variable estimator from Equation S2, the estimated β will be too large in the positive direction (towards zero) compared to the true effect. If the confounding process is a mix of the two, the two estimates bracket the true expected causal response (*3*). We estimate Equation S2 using the "reghdfe" command in STATA (v.16) and the 'feols' command in R using the fixest package (v. 0.8.2).

As expected, the estimated effect from Equation S2 is less negative (smaller in absolute magnitude), providing an upper bound on the effect of species richness on biomass (Table S8). Still, this estimate implies a negative effect of richness on productivity, with the lower 95% confidence interval overlapping the estimate from the estimator in the Main Design (Equation S1).

Finally, *what if the true system were characterized by Fig. S6 and not approximated by either Fig. S7 or Fig. S8?* Then we would have to make more assumptions to identify the effect of richness on productivity. For example, if we are willing to assume that the persistent causes of productivity across years (*I*) comprises autoregressive disturbances of order 1, the effect of *R* on *P* can be estimated using an autoregressive distributed lag equation of order 2 in autoregression and order 1 in distributed lags (*65*). Using the "reghdfe" command in STATA (v.16) and the 'feols' command in R using the fixest package (v.0.8.2), we estimate the following model:

$$\ln LiveMass_{pst} = \beta \ln Richness_{pst} + \beta' \ln Richness_{ps(t-1)} +$$

$$\Omega \ln LiveMass_{ps(t-1)} + \Omega' \ln LiveMass_{ps(t-2)} + \mu_{st} + \varepsilon_{pst} \quad \text{(S3)}$$

Table S8. Results from the Dynamic Panel Design. Results present coefficient estimates of a 1% increase in the ln richness on ln of productivity (measured as live biomass); clustered robust standard errors are shown in the parentheses, and 95% confidence intervals in brackets. To see 95% confidence intervals as well, see the project page (TableS8_R_CI.tex).

	ln(Productivity)
ln(SR)	**-0.1329***
	**(0.0789)**
	**[-0.2876, 0.0218]**
$\ln LiveMass_{ps(t-1)}$	0.1374***
	(0.0377)
	[0.0634, 0.2113]
Num. obs.	1063
R^2 (full model)	0.83

S*ignif. Codes*: ***: 0.01, **: 0.05, *: 0.1
Robust Standard errors in parentheses (clustered at plot level).

When we estimate this equation S3, our estimated effect of richness on productivity is similar to our main estimate in Fig. 2: a 10% increase in richness leads to a 2.4% decrease in productivity, 95% CI [-4.4, -0.4]. Adding one more lag for richness and productivity (i.e., including $\ln Richness_{ps(t-2)}$ and $\ln LiveMass_{ps(t-3)}$) yields a similar estimate of -2.0% for ln $Richness_{pst}$.

As a final estimator, we follow the suggestion of a peer reviewer and combine the Main Design estimator (Equation S1) and the Lagged Dependent Variable design (Equation S2). This estimator is potentially biased for reasons that can be found in other publications (e.g., (*3*), *Sections 5.3-5.4*). The intuition is that the combination of δ_{ps} from Equation (S1) and $LiveMass_{ps(t-1)}$ from Equation (S2) can create a correlation between richness and the error term in the model, which makes the estimator inconsistent (i.e., the estimator will not converge in probability to the true value of our target parameter even as the number of plots in our panel

goes to infinity). This problem is not caused by an autocorrelated error process. The problem arises even if the error process is *i.i.d*. If the error process is autocorrelated, the problem is even more severe. Despite this potential bias, the estimated effect is similar to the estimates from the other approaches: a 10% increase in richness, on average, decreases productivity by 2.8% [95% CI: -4.6%, -1.1%].

Note: In Figures S6-S8, we do not include a graph in which $P_{t-1} \rightarrow R_t$, $R_t \rightarrow P_t$, and no other direct edges exist. We exclude this graph because this pattern is not ecologically possible (i.e., a case where prior productivity had an effect on current productivity and richness would be the sole mediator or, equivalently, prior productivity has no effect on current productivity except through its effect on current richness).

S6b.ii. Design sensitivity to unobserved, plot-level confounding variables

Imagine a set of unobserved, time-varying, plot-level confounding variables that are negatively associated with richness and positively associated with productivity (so-called "negative selection bias"). Were such a set of confounding variables to exist in our system, the true average causal response in our sample could be closer to zero or positive. However, as we will see below, using time-invariant plot attributes and time-varying site attributes, we can explain about 90% of the variation in annual richness and productivity. Thus, there is little variation left that could come from this unobserved variable and induce bias in our estimator. Nevertheless, we test the sensitivity of our inferences to such a confounding variable.

Following the method introduced by Altonji et al. (*66*) and further developed by Oster (*67*), we assume that the relationship between species richness and the omitted, unobserved confounding variables can be characterized using information from the relationship between the richness variable and the variables in Equation (S1). Oster in (*67*) assumes that observable covariates contribute (approximately) proportionally to treatment and to the outcome (i.e., the time-invariant plot attributes and the time-varying site attributes are as important in explaining richness as they are in explaining productivity conditional on richness). If that assumption is a reasonable approximation of the truth, which seems credible given our data (Table S7), we can then explore the conditions under which the omitted variables could change our conclusions. Said another way, this analysis answers the question, "How much correlation between the unobserved variable and the richness/productivity variables would be sufficient to change our conclusions?"

Recall that our preferred estimation Equation (S1) includes variables capturing time-invariant plot attributes (δ_p) and time-varying site attributes (μ_{st}). That equation is most easily and efficiently estimated by taking first differences or deviations from means, so that δ_p drops out before estimating the coefficient β_1. Thus, any goodness-of-fit measure, like R^2, would not include the role of the plot fixed effects in explaining variation in productivity.

However, estimating Equation (S1) is equivalent to estimating the following specification:

$$\ln\,(Biomass)_{pst} = \beta_0 + \beta_1 \ln\,(Richness)_{pst} + \mu_{st} + \sum_{ps} \alpha_{ps} d_{ps} + \epsilon_{pst} \qquad \text{(S4)}$$

where the variables are as before but rather than eliminate <u>time-invariant</u> plot attributes (δ_{ps}) by taking deviations from the mean (*Section S4*), we model them through a new set of variables: d_p is a set of plot dummy variables and α is a vector of coefficients of the plot dummy variables. We can estimate this equation using the ‘areg’ command in STATA (v.16).

The estimated β_1 will be the same as the estimated β from our deviations-in-means estimation of Equation (S1), although the estimated standard errors will be larger using equation (3) (i.e., estimating Equation (S4) is less efficient than estimating Equation (S1) via deviations in means). These differences in estimated standard errors come from clustering the standard error estimation at the plot-level. Clustering standard errors in the ‘areg’ procedure adjusts the degrees of freedom by the number of fixed effects removed in the within-group transformation. In contrast, the reghdfe (or xtreg fe) procedure does not make such an adjustment and thus reports smaller cluster-robust standard errors. Thus, to estimate the effect of richness on productivity, the xtreg fe or reghdfe estimation procedure is preferred. However, for our sensitivity analysis, using the ‘areg’ procedure is preferred because it allows us to use the time-invariant, plot-level characteristics as control variables, rather than treat them as nuisance parameters that can be eliminated via first-differencing or taking deviations from means. Table S9 (column 1) reports the results from the areg regression with clustered standard errors at the plot level. As expected, the ‘areg’-estimated coefficient on species richness is the same as the estimate in the main text, but with a larger standard error.

To explore the sensitivity of our design to an unobserved confounder, or set of confounders, Oster (*67*) shows that one must first make an assumption about the R-squared value from a hypothetical regression of productivity on the unobserved confounder and the variables in Equation (S4). If this value, called R_{max}, were set equal to one, it would be equivalent to saying

that the variation in annual productivity can be fully explained by the hypothetical regression model. In other words, the unobserved confounder explains all the unexplained variation in regression specification (S4), an assumption that implies there is no measurement error in the live biomass measure of annual plot productivity. The R-squared from the regression specification (S4) is 0.87 (Table S9, column1). Thus, an assumption that R_{max} = 1 implies that the unobserved confounder explains all remaining variation in annual productivity, which is an implausibly powerful predictor of productivity (i.e., an implausibly strong confounder).

Table S9. Regression results to support sensitivity analysis to hidden bias.

	(1)	(2)
	Outcome Equation: Productivity	Selection Equation: Species Richness
Species Richness	-0.24<br>(0.09)<br>[-0.42, -0.06]	
R-Squared	0.87	0.91
Number of Plots	151	151
Number of Sites	43	43
Number of Observations	1231	1231

The first column replicates our main result from column 1, Table S2 but uses a dummy variable procedure to control for plot-level fixed effects rather than a deviations-from-means procedure (and thus standard error estimates are slightly larger in this table). The second column regresses plot-level richness on plot dummy variables and the year-by-site variables. The sample size is larger in second column because fewer plots have missing richness values than have missing productivity values. Robust standard errors in parentheses (clustered at plot level) and 95% CI in brackets.

We next specify the magnitude of the degree of selection on unobservable variables relative to the selection on observable variables. This parameter π (called δ in Oster's article) yields a measure of our design's sensitivity to hidden bias: how would our results change were there an unobserved confounder (or set of confounders) that is correlated with richness and productivity? The unobserved confounder is assumed to be uncorrelated with the other variables in our equation; it is most easily envisioned as a time-varying confounder that is orthogonal to our site-by-year variables and our time-invariant plot variables. To help determine a plausible value for π, we use 'areg' to estimate the relationship between the richness and the observable covariates in Equation (S4) using the following specification:

$$\ln(Richness)_{pst} = \beta_0 + \mu_{st} + \sum_{ps} \alpha_{ps} d_{ps} + \epsilon_{pst} \qquad \text{(S5)}$$

Results from this regression are reported in Table S9. The R^2 from this regression (Table S9, column 2) implies that the variables in our regression specification already explain about 91% of the variation in annual plot richness. Setting the value of π equal to -0.10 would be equivalent to assuming that the unobserved confounder explains all the remaining variation in richness (i.e., the unobserved confounder explains about 9% of the variation in annual plot richness).

Setting π = -0.10 and R_{max} = 1 would mimic a powerful potential unobserved confounder in our design: a confounder that is so strongly correlated with productivity and richness that, were we able to observe it (along with the other variables in the equation), we could predict with near certainty which of two plots would have higher productivity and which would have higher richness. Estimating the effect of richness on productivity with those implausible parameter values yields an upper bound on the impact of richness on productivity. For completeness, we also calculate a lower bound on the impact by setting π = 0.10 (i.e., positive selection bias).

To estimate these bounds, we use the 'psacalc' package in Stata (v.16) as a post-estimation function after estimating the effect of richness on productivity using Stata's 'areg' command. The estimated upper bound is still negative: a 10 % increase in richness implies a 2.0% decrease in productivity. In other words, in the presence of an unobserved confounder that is negatively associated with richness and positively associated with productivity relationship (thus creating some spurious correlation between richness and productivity), we would still infer that there is a negative relationship between richness and productivity. To infer a positive relationship between the two variables would require an infeasible value for π: it requires π>1, which implies the confounder would have to be more influential in explaining variation of productivity than the plot-level, time-invariant attributes and the site-level, time-varying attributes. We also consider an unobservable confounder that is masking some of the negative effect of richness on productivity. If the unobserved confounder was positively associated with both richness and productivity, a 10% increase in richness would decrease productivity by an estimated 3.0%.

Overall, the sensitivity analysis implies that our estimated effect of richness on productivity is not sensitive to the presence of a time-varying confounder.

S7. Comparison of Main Design to Common Designs in Ecology

Here, we refer to multi-level modeling, hierarchical modeling, random effects modeling, mixed effects modeling, or variance components modeling as a Common Design in Ecology with which we compare our Main Design. Ecologists who are familiar with multi-level modeling may wonder why, given our data are comprised of plots nested within sites and annual observations nested within plots, we do not use this multi-level modeling approach as our main design. A full explanation is beyond the scope of this SI, but the main reason has two parts:

(a) Without more variable transformations, the multi-level modeling approach does not easily lend itself to controlling for as many unobservable sources of confounding as can be done in our linear, additive, fixed-effects panel data estimator. Note that our use of the term 'fixed effects' corresponds to the use of the term in econometrics (*3*, *4*) – and the meaning differs from the use in multi-level modeling uses in Ecology; see Glossary S1 for more details. The typical multi-level model assumes "selection on observables" (see Glossary S1). In other words, the model requires stronger assumptions to infer causality from the correlation between richness and productivity; it assumes that there is no correlation between unobserved components of the error term at the site and plot levels and the species richness variable (*17*, *44*). This assumption is easily violated in this dataset. In other words, the multi-level model assumes that the propensity of plots to experience change in their species richness is not determined by variables that also affect productivity and are not explicitly in our model (i.e., not determined by variables in the error term). In contrast, in our panel data design, the time-invariant individual and site attributes are no longer part of the error term. Thus, we do not have to make that strong "selection on observables" assumption made by traditional multi-level models.

(b) Clustering our standard errors at the plot provides the same benefits that multi-level modeling does when estimating the variance-covariance matrix in the presence of intra-site correlations among plots (*44*).

The disadvantage of our design (i.e., Equation S1) is that, in the process of eliminating the role of confounding variables, we also eliminated variables that affect productivity but do not affect richness (*I* in Figure 1B) – i.e., both the "bad" between-plot (confounders) and "good" between-plot variation (predictors) are eliminated, thus reducing statistical power (increasing

standard error estimates) and limiting the scope of some of the analysis that can be performed (*68*). For example, we cannot, in our study, estimate the associations between productivity and time-invariant attributes like soils or climate. Some other research aims, like estimating how the effect of richness on productivity varies by site (versus site-level moderators), are easier to accomplish in multi-level modeling designs.

In our study, however, we are focused on estimating the causal effect of biodiversity on productivity. Thus, in our study context, the traditional multi-level modeling approach – which aims to control for observable confounding variables by including them in the model - has no advantages over our approach, and a serious disadvantage in its inability to control for unobservable sources of confounding variation without data transformation that mimics what happens in our estimation of Equation (S3) (*see last paragraph in this section for details*). Indeed, not controlling for unobservable sources of confounding variation leads to bias in estimates.

Nevertheless, to demonstrate how much design matters in drawing inferences from observational data, we estimate a traditional random-effects equation by using a GLS estimator

$$\ln LiveMass_{pst} = \beta \ln Richness_{pst} + \sum \gamma X_p + \sum \theta X_s + \vartheta_t + \varphi_p + \rho_s + \varepsilon_{pst} \quad \text{(S6)}$$

where φ_p and ρ_s are parts of the error term and X_p and X_s are observable attributes of plot and site, respectively. **This equation represents the "Common Design in Ecology" that we report in the main text.** Unlike Equation (S1), this equation uses both the within-plot variation in species richness and the between-plot variation in richness to estimate the effect of richness on productivity (i.e., the estimator produces a matrix-weighted average of the between-plot and within-plot estimates). The between-plot variation in species richness can be caused by many attributes that may also be correlated with productivity. We follow a traditional approach in ecology and attempt to control for (block) the confounding effects of these attributes by measuring them and adding them to the equation (X_p and X_s). In the words of the multi-level modeling community, the γ and the θ are "fixed effects" and the φ_p and ρ_s are "random effects." The "fixed effects" are directly estimated whereas the "random effects" are not, but rather summarized according to their estimated variances and covariances.

Because our data set is large, we can control for over 60 observable confounders, including variables for soil attributes, habitat types, historical management categories, weather, year, country, and elevation (see Table S10). In other words, we can control for a wide range of variables that may affect both richness and productivity. Along with species richness, these variables explain 57% of the overall (spatial and temporal) variation in productivity (live biomass) and 95% of between-plot (spatial) variation in productivity.

Using this design, the estimated relationship between richness and productivity is much more in line with the conventional wisdom in the ecology literature: a 10% increase in plot richness increases plot productivity, on average, by 3.8%, 95% CI [0.1%, 7.5%]. In other words, when we do not leverage the spatial and temporal variation to eliminate the effects of unobserved plot and site attributes (i.e., our Main Design), we draw the opposite conclusion about the relationship between richness and biomass.

The problem with this *Common Design in Ecology* is too few control variables, not too many. Failing to control for a confounding variable leads to omitted variables bias (reviewed in (*69*)), yet it is impossible to know or measure all confounding variables in a complex ecosystem system. Thus, the problem cannot be solved by adopting a "model selection" procedure to select, based on some measure of prediction error, a subset of the 60 variables (e.g., forward-selection or backward-selection procedures). Including control variables that have no correlation with the outcome will indeed add noise to the estimation procedure and unnecessarily reduce the precision of the estimated effect of richness on productivity. But precision is not a problem in our study because of our large sample size. Potential bias is the problem. Thus, the choice of control variables must not be driven by statistical benchmarks but rather by theory and field experience about which ecosystem features may affect both richness and productivity. One can only justify eliminating control variables from the model if one believes that the remaining covariates eliminate the correlation between richness and the model error term, which can only be justified based on theory or field experience.

A multi-level structural equation model (SEM) would be more flexible than the multi-level regression we implement in Equation (S6), but if we used the same observable variables, it would not affect the coefficient estimates. Use of a SEM would affect the standard error estimates, which is not relevant to make our point related to how our inference on the sign of the estimated effect of richness on productivity switches across designs. SEMs are also useful for

estimating mediator effects (e.g., by specifying direct and indirect paths), estimating the relationship between observed and latent variables, and developing predictive models of productivity, but none of those aims are relevant for our study design.

Table S10. Control Variables in Common Design in Ecology

**Attribute**	**Covariate Variables**
Country variables	Australia (AU), Canada (CA), Switzerland (CH), Germany (DE), Tanzania (TZ), United Kingdom (UK), United States (US)
Habitat variables	Alpine grassland, Annual grassland, Desert grassland, Mesic grassland, Montane grassland, Old field, Pasture, Savanna, Semiarid Grassland, Shortgrass prairie, Shrub steppe, Tallgrass prairie
Observation year variables (Year in site's panel data set)	1st, 2nd, 3rd, 4th, 5th, 6th, 7th, 8th, 9th, 10th, 11th
Historical site management variables	Active management (otherwise wild), Active managed burning regime, Regularly grazed by herbivores, Restored
Topographical variables	Elevation (meters)
*Weather variables**	Temperature Seasonality (standard deviation *100), Max Temperature of Warmest Month, Min Temperature of Coldest Month, Mean Temperature of Wettest Quarter, Mean Temperature of Driest Quarter, Mean Temperature of Warmest Quarter, Mean Temperature of Coldest Quarter
Soil physical property variables	Soil Percent Sand, Soil Percent Silt, Soil Percent Clay
Soil fertility variables	Soil Percent Carbon by Mass, Soil Percent Nitrogen by Mass, Soil Phosphorus by Mass (ppm), Soil Potassium by Mass (ppm), Soil Calcium by Mass (ppm), Soil Magnesium by Mass (ppm), Soil Sulfur by Mass (ppm), Soil Sodium by Mass (ppm), Soil Zinc by Mass (ppm), Soil Manganese by Mass (ppm), Soil Iron by Mass (ppm), Soil Copper by Mass (ppm), Soil Born by Mass (ppm), pH

* The weather variable values in NutNet are site-level averages over time (i.e., they are time-invariant). Thus, controlling for temperature variables listed in Table S10 also controls for the precipitation variables, evapotranspiration variables, and other weather variables. In other words, the temperature variables serve as site-level indicator variables and the estimated effect would be the same if, for example, we used precipitation variables instead of temperature variables.

The positive estimated effect from Equation (S6) is not driven by having to drop sites that did not measure all the covariates (the sites in France, Portugal, and South Africa did not collect the soil data). If we use only the 675 observations from the multi-level modeling in our Main Design, we still obtain a negative estimated effect of richness on productivity, albeit less precisely estimated because of the smaller sample size: a 10% increase in plot richness decreases

plot productivity, on average, by 3.11%, i.e., the estimated effect is - 3.11%, with 95% confidence interval of [-6.31%, 0.09%]. Thus, the contrast between the Main Design and the Common Design in Ecology is not affected by the change in the sample composition.

The key issue that we highlight is one of "design" – not of methods (i.e., type of estimation procedure). In principle, one could accomplish the same objectives of our design within a multi-level or SEM framework by using a group-mean centering transformation of the data (i.e., within-plot centering of time-varying richness; (*68*, *70*)). The key innovation in our *design* is to leverage the panel data to control for a wide range of time-invariant plot attributes and time-varying site attributes -- *a wider range of confounders than previous studies have addressed*. Whether that leverage is exploited in a single regression equation or in a system of regression equations matters little for the estimation of the effect of plot richness on plot productivity.

S8. Supplementary Methods: Heterogeneous Effects of Rare, Non-rare, and Non-native Species on Productivity

To shed light on the reasons why an increase in species richness reduces productivity, on average, we decompose species richness into groups of species. Using the plot-level data from the Nutrient Network (see section S8a), we first decompose overall species richness into native versus non-native species and rare versus non-rare species (by "non-rare", we mean dominant and subordinate species). We then create groups for four categories of species that combine rarity and non-native status: (1) rare and native, (2) rare and non-native, (3) non-rare and non-native, and finally (4) non-rare and native.

As in most ecosystems worldwide (*71*), and consistent with theory (*72*), the rank abundance curves of species from our sites imply that most species in these ecosystems are rare (Figure S10). Thus, not surprisingly, higher species richness at our 43 sites is associated with, on average, higher numbers of rare species (Fig S11(A)). Moreover, as in many natural ecosystems in the Anthropocene (*73*), higher species richness at our 43 sites is also associated with, on average, higher numbers of non-native species (Fig S11(B)). Thus, higher numbers of species tend to be associated with more rare and non-native species in a place, and these species may have different effects on productivity than native non-rare species (e.g. *60*, *61*). Our analysis

contributes to our understanding of how the effect of species richness on productivity depends on the characteristics of the biodiversity changing.

All code to reproduce the data processing in section S8 can be found at the project page. This code was checked by 3 additional people beyond the lead author.

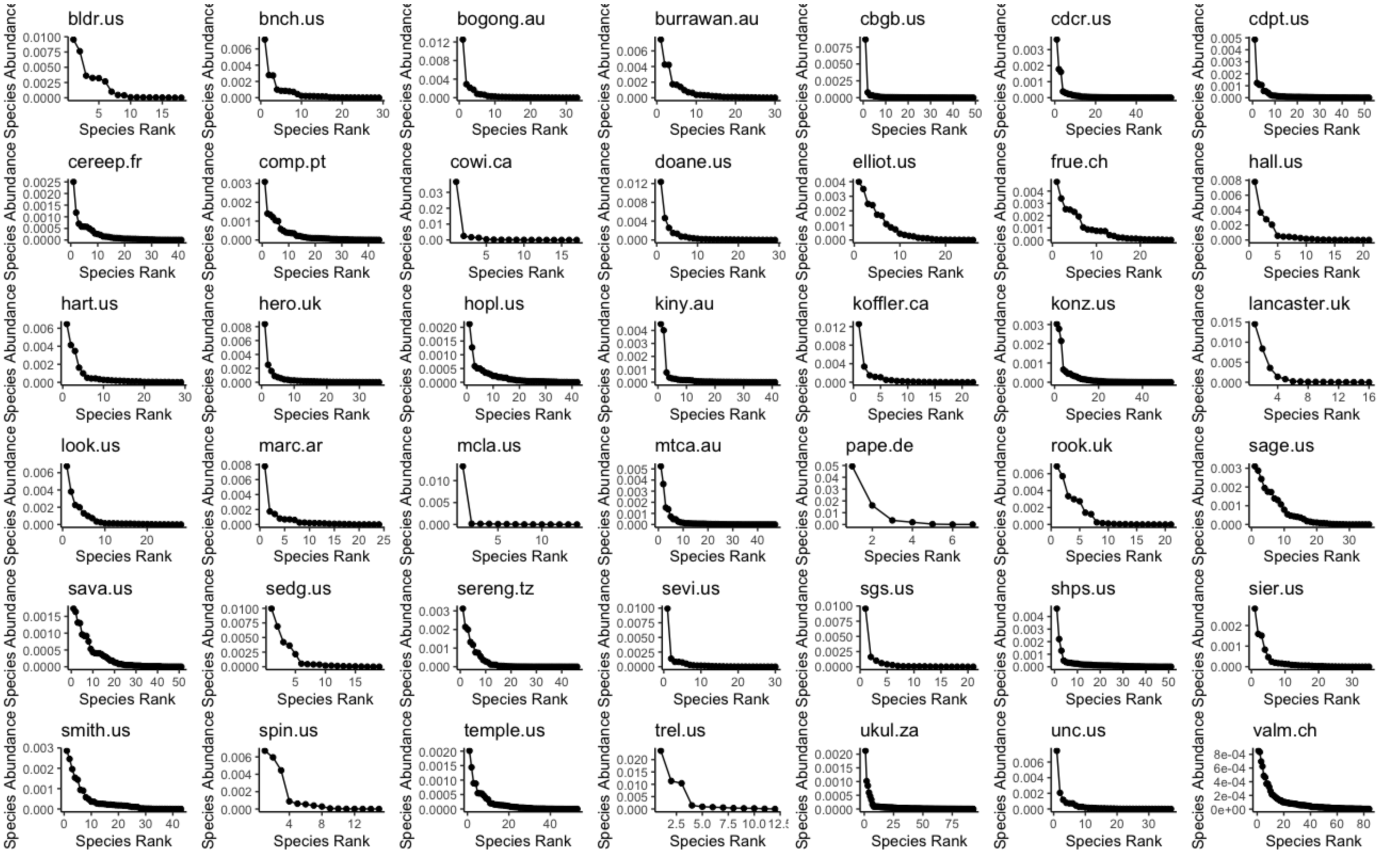

Figure S10. Most species are rare in these grassland ecosystems. Rank abundance curves (RAC) for each Nutrient Network site in our analysis shown in Table S1. These RACs are for the pre-treatment year, which we use to define species as rare or non-rare. Here, species abundance is based on relative live cover at the site-level.

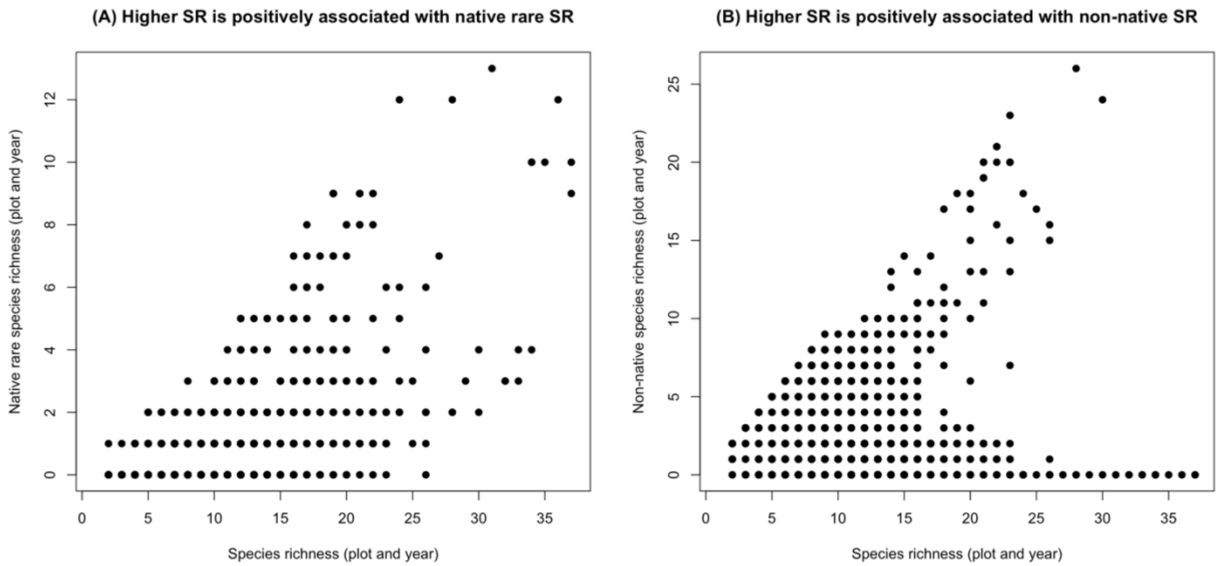

Figure S11. Greater diversity, in terms of more numbers of species, is associated with more (A) native rare species and (B) non-native species, on average. We plot the association between overall species richness (SR) and counts of native rare species and of non-native species, per plot and year.

S8a. Definitions and Measurement of groups in Figure 5

To classify species by rarity and origin, we use plot-level data from the entire site (i.e., not just our 151 unmanipulated plots). The rarity designation is based on measures of relative abundance at the site. To ensure that our relative abundance measures are unaffected by the experimental manipulations at the site, we use data only from the pre-treatment years. Species absent during the first year were treated differently (see below).

Note that because we classify the species in the groups based on pre-treatment year data, the site "saline.us" is excluded from this analysis because the site does not have pre-treatment data. Dropping the 24 observations from the site "saline.us" does not change our estimates in Figure 2 and 3.

Native versus Non-native Species

In the Nutrient Network, species origin was determined by the site coordinators and designated by one of three categories: "native", "INT" (i.e., non-native), or "unknown origin" (see "ProcessNutNet_coverData_FINAL_public.R" code on our project GitHub release DOI/10.5281/zenodo.7675340 for more details). We compute the number of species that are classified as native and non-native in each plot in each year and then construct species richness

variables for each species type in each plot in each year. We drop the species of "unknown origin" present in the pre-treatment year in our main analysis, but to consider how the uncertainty about the origin of some species in the data could affect our conclusions, we performed a bounding approach where we re-estimate the effects by first assuming all unknown origin species are native and then assuming they are all non-native (see *S8ci. Sensitivity Analyses: Species with unknown origin*).

Classifying Rare versus Non-Rare Species

We assign the labels of "rare" and "non-rare" to species in multiple ways, using definitions based on two metrics for relative abundance of a species at a site: the relative cover of each species at a site and the relative frequency of each species at a site. To calculate relative cover and relative frequency, we use live cover only in the pre-treatment year. Next, we classify species as "rare" based on their cover and frequency relative to other species at each site (see below). We then compute the number of species that are classified as rare or non-rare in each plot in each year and then construct species richness variables for each species type in each plot in each year. Below, we describe each step in this procedure.

In the main text, we define "rare" species using the relative cover metric. We use relative cover as our metric for abundance, because we believe better captures the range of mechanisms through which rare species may decrease productivity, including taking space formerly occupied by more productive species. In *Section S8c.ii*, we report the results using the relative frequency metric. In *Section S8c.iii*, we also test the sensitivity of our conclusions to different cutoff values for assigning a species to the "rare" and "non-rare" categories.

Computing the relative cover metric

For each species present in the pre-treatment year, we computed the relative cover as the sum of the plot cover of the species for all plots at the site, divided by the total cover in all plots at that site. Note that some plots exceed 100% cover, whereas other plots are <100%; thus, we standardize this metric by dividing the sum of the maximum cover of a species in each plot at a site by the total live cover in all plots at a site.

Classifying species as rare or non-rare using relative cover

Using the relative cover metric, we classify species into three categories: dominant, subordinate, and rare. The categorization of rare, subordinate, and dominant species is based on the quantiles of the species' relative cover data for each site, created using the 'quantile' function in R.

The first classification, presented in the main text Figure 5 and Table S10, labeled species at a site with relative cover in the lowest 60% of the site-level distribution (0.6 quantile) to be rare and species in the top 95% of the distribution (0.95 quantile) to be dominant. The species with relative cover in between these two cut-off values were labeled subordinate. These cut-off values lead to a maximum of 1 to 4 species dominant species per site, consistent with (*76*), and a median of 2 dominant species (average of 2.3). This cut-off leads to an average of 21.7 rare species and median of 20 rare species per site and an average of 12.73 and median of 12 subordinate species per site. To maintain objectivity in the analysis, the person who recommended these cut-off values (Kaitlin Kimmel) was not the person running the estimation analyses. To assess whether the cut-off values generated a sensible classification of species – particularly with regard to differentiating dominant from rare species – the person who recommended the cut-off values (co-author Dr. Kaitlin Kimmel) checked which species were labeled "dominant" at two sites about which she had extensive knowledge (cdcr.us and knz.us). She confirmed that the three species that were labeled dominant at each site were indeed what experts would label as the dominant species.

Given that few species are dominant at each site and, by definition, these species do not exit and enter plots with great frequency, we combine the numbers of dominant and subordinate species into the non-rare species richness variable. We test the sensitivity of our results to the choice of cut off values for classifying species as rare or non-rare (Section S8c.iii).

Several species were not observed in the first year of data collection at a site, implying that those species had a relative cover and relative frequency of 0 in that pre-treatment year. However, rather than assume these species are rare or non-rare, we classified these species separately (as "NA species") and controlled for them in our analyses (see Section *S8c.i*).

Once we classify species are "rare", "non-rare" and "NA" based on pre-treatment data, we then count the number of species in each combined category for each plot and year.

S8b. Statistical Analyses

Combining the classification of species by origin and the classification of species by rarity, we can count the number of Non-Rare Native species, Rare Native species, Non-Rare Non-Native Species, Rare Non-Native species, and species classified as NA for each plot and year. We then substitute the richness variables of each species category for the overall "species richness" variable used in our main Equation (S1). In other words, we substitute the five categories of richness for the single "richness" variable of Equation (S1):

$$\ln(LiveMass_{pst}) = \beta_{DN}\, ihs(\,NonRareNative_{pst}) + \beta_{RN}\, ihs(\,RareNative_{pst}) + \beta_{DNN}\, ihs(\,NonRareNative_{pst}) + \beta_{RNN}\, ihs(\,RareNonNative_{pst}) + \beta_{NA}\, ihs(\,NAspp_{pst}) + \delta_{ps} + \mu_{st} + \varepsilon_{pst} \qquad \text{(S7)}$$

The species richness variables are transformed with an inverse hyperbolic sine transformation rather than a natural logarithm transformation. The inverse hyperbolic sine transformation is analogous to a log-transformation but can be used when there are many 0 observations (*52*). Given the inverse hyperbolic sine transformation of the richness variables, the estimated effects cannot be interpreted as elasticities without further manipulation, but their signs and relative magnitudes can be compared to each other.

S8c. Comparing the effect of species richness per group on productivity

In Figure 5 and Table S11, we present the estimated effects of species richness on productivity, conditional on species type, using relative cover to classify species as non-rare or rare. The estimates imply that an increase in species richness has a positive effect on productivity when the increase is coming from non-rare, native species or from rare, non-native species. But for rare, native species, as well as for non-rare, non-native species, the estimated effects are negative. The estimated effect is largest in absolute value for rare, native species and non-rare, non-native species. We reject the null hypotheses that changes in the richness of these groups of species have an equivalent effect on live mass (*Chisq* = 9.8205, *Pr*(>*Chisq*) = 0.02016).

Controlling for the number of species labeled "NA" that enter the plots after the pre-treatment year (which we call "NA species richness") does not change the inferences drawn (Table S11 – i.e., compare column *1 versus* column 2). In the main text Figure 5, we present the conservative model that controls for the NA species richness.

Table S11. The effect of species richness (SR) on biomass production conditional on species type, using species relative cover to determine rare versus non-rare species. We estimate Equation (S1) with species richness disaggregated into the numbers of non-rare native species, rare native species, non-rare non-native species, and rare non-native species. In column 1, we controlled for the number of species not found in the pre-treatment year at site; in column 2, we perform a sensitivity analysis, dropping that species count. All estimated effects of each category of species richness (SR) are on a log-inverse-hyperbolic-sine scale. Given the inverse hyperbolic sine (his) transformation of the species richness variables, the estimated effects cannot be interpreted as elasticities without further manipulation, but their signs and relative magnitudes can be compared to each other. Clustered-robust standard errors are used and clustered at the plot level. To see 95% confidence intervals as well, see the project page (output/TableS11_R_CI.tex).

	Main Text	*Dropping counts of NA species*
Non-rare, Native SR	0.0488	0.0507
	(0.0721)	(0.0723)
Non-Rare, Non-Native SR	-0.1721***	-0.1794***
	(0.0649)	(0.0651)
Rare, Non-Native SR	0.0397	0.030
	(0.0711)	(0.0701)
Rare. Native SR	-0.1473***	-0.1429***
	(0.0459)	(0.0466)
SR of NA species	-0.0901**	
	(0.0430)	
Num. obs.	1,175	1,175
Num. plots	146	146
R^2 (full model)	0.79	0.79

S*ignif. Codes*: ***: 0.01, **: 0.05, *:0.1
Robust Standard errors in parentheses (clustered at plot level).

S8ci. Sensitivity Analyses for species with unknown origins

Several species were classified as of "unknown origin." The analyses presented in Table S11 and S13 and Figure 5 omit species of unknown origin from the groups. To test the sensitive of our results to this uncertainty, we bound the estimated effects by considering two possible extreme scenarios: 1) all species of unknown origin are native and 2) all species of unknown origin are non-native. Thus, we revise the species groups in Equation (S7) and re-rerun the analyses with these two sets of models to establish bounds. The signs and magnitudes of the estimated effects in Tables S12 and S13 are similar to those in Table S11.

Table S12. Sensitivity Analyses: treating species of unknown origin as native. We estimate Equation (S1) with species richness disaggregated into the numbers of non-rare native species, rare native species, non-rare non-native species, and rare non-native species. Here we treat species of unknown origin as native (rare or non-rare). In column 1, we controlled for the number of species not found in the pre-treatment year at site; in column 2, we perform a sensitivity analysis, dropping that species count. All estimated effects of each category of species richness (SR) are on a log-inverse-hyperbolic-sine scale. Given the inverse hyperbolic sine transformation of the species richness variables, the estimated effects cannot be interpreted as elasticities without further manipulation, but their signs and relative magnitudes can be compared to each other. Clustered-robust standard errors are used and clustered at the plot level. To see 95% confidence intervals as well, see the project page (output/TableS12_R_CI.tex).

	Controlling for NA species	*Dropping counts of NA species*
Non-rare, Native + unknown SR	0.0848	0.0878
	(0.0712)	(0.0719)
Non-Rare, Non-Native SR	-0.1717***	-0.1796***
	(0.0640)	(0.0646)
Rare, Non-Native SR	0.0367	0.0263
	(0.0704)	(0.0693)
Rare. Native + unknown SR	-0.1399***	-0.1333***
	(0.0458)	(0.0470)
SR of NA species	-0.0914**	
	(0.0432)	
Num. obs.	1,175	1,175
Num. Plots	146	146
R^2 (full model)	0.79	0.79

S*ignif. Codes*: ***: 0.01, **: 0.05, *:0.1
Robust Standard errors in parentheses (clustered at plot level).

S8cii. Sensitivity analyses using relative frequency as a metric for rarity

We next check the robustness of the results to our choice of metric for determining rarity, comparing our results in Figure 5 to estimates when defining rarity based on relative frequency, rather than relative abundance. We calculate relative frequency as the number of plots that a species occurs in a year divided by the total number of plots at a site in each year.

The inferences about the effects of different species groups on productivity are similar using frequency instead of cover (Table S14). The Zenodo release of project page also includes results for relative frequency dealing with unknown species origin as above in section *S8c.i.* Results are similar; for more details see the project page (/output/TableS14Sensitivity_R_se.tex).

Table S13. Sensitivity Analyses: treating species of unknown origin as non-native. We estimate Equation (S1) with species richness disaggregated into the numbers of non-rare native species, rare native species, non-rare non-native species, and rare non-native species. Here we treat species of unknown origin as non-native (rare or non-rare). In *column 1*, we controlled for the number of species not found in the pre-treatment year at site; in *column 2*, we perform a sensitivity analysis, dropping that species count. All estimated effects of each category of species richness (SR) are on a log-inverse-hyperbolic-sine scale. Given the inverse hyperbolic sine transformation of the species richness variables, the estimated effects cannot be interpreted as elasticities without further manipulation, but their signs and relative magnitudes can be compared to each other. Clustered-robust standard errors are used and clustered at the plot level. To see 95% confidence intervals as well, see the project page (output/TableS13_R_CI.tex).

	Controlling for NA species	*Dropping counts of NA species*
Non-rare, Native SR	0.0451	0.0458
	(0.0725)	(0.0726)
Non-Rare, Non-Native + unknown SR	-0.1362**	-0.1382**
	(0.0669)	(0.0666)
Rare, Non-Native + unknown SR	0.0512	0.0514
	(0.0617)	(0.0616)
Rare. Native SR	-0.1500***	-0.1467***
	(0.0456)	(0.0462)
SR of NA species	-0.0916**	
	(0.0432)	
Num. obs.	1,175	1,175
Num. Plots	146	146
R^2 (full model)	0.79	0.79

S*ignif. Codes*: ***: 0.01, **: 0.05, *:0.1
Robust Standard errors in parentheses (clustered at plot level).

Table S14. Sensitivity Analysis using Relative Frequency. We determine species groups and the effect of species richness (SR) on biomass production conditional on species type, using species relative frequency to determine rare versus non-rare species. We estimate Equation (S1) with species richness disaggregated into the numbers of non-rare native species, rare native species, non-rare non-native species, and rare non-native species. In *column 1*, we controlled for the number of species not found in the pre-treatment year at site; in *column 2*, we perform a sensitivity analysis, dropping that species count. All estimated effects of each category of species richness (SR) are on a log-inverse-hyperbolic-sine scale. Given the inverse hyperbolic sine transformation of the species richness variables, the estimated effects cannot be interpreted as elasticities without further manipulation, but their signs and relative magnitudes can be compared to each other. Clustered-robust standard errors are used and clustered at the plot level. To see 95% confidence intervals as well, see the project page (output/TableS14_R_CI.tex). Also, please find other sensitivity analyses as done in Table S12 and S13 but using relative frequency on the project page (analyses_fig5_smsection8.R). The conclusions remain unchanged.

	Controlling for NA species	*Dropping counts of NA species*
Non-rare, Native SR	0.0411	0.0505
	(0.0804)	(0.0825)
Non-Rare, Non-Native SR	-0.1739**	-0.1844***
	(0.0688)	(0.0685)
Rare, Non-Native SR	-0.0126	-0.0183
	(0.0606)	(0.0592)
Rare. Native SR	-0.0936*	-0.0914*
	(0.0500)	(0.0500)
SR of NA species	-0.0834*	
	(0.0420)	
Num. obs.	1,175	1,175
Num. plots	146	146
R^2 (full model)	0.78	0.78

S*ignif. Codes*: ***: 0.01, **: 0.05, *:0.1
Robust Standard errors in parentheses (clustered at plot level).

S8ciii. Sensitivity Analyses using different cut-offs for rare versus non-rare categories

To assess the sensitivity of the results to the classification criteria for rare and non-rare species, we use two additional cut-offs. Cut-off 2 labels species at a site with relative frequency in the lowest 70% of the site-level distribution (0.7 quantile) to be rare and species the top 95% of the distribution (0.95 quantile) to be dominant. The species with relative cover in between these two cut-off values were labeled subordinate. Cut-off 3 labels species at a site with relative frequency in the lowest 50% of the site-level distribution (0.5 quantile) to be rare and species the top 95% of the distribution (0.95 quantile) to be dominant. The species with relative cover in

between these two cut-off values were labeled subordinate. As in the analysis for Table S11, the subordinate and dominant species were grouped together in the “non-rare” category. As shown in Table S15, these changes in the cut-off criteria do not change the signs of our estimates and have little effect on their magnitudes.

Table S15. Sensitivity Analyses: Comparing inferences when using different cutoffs for defining species as rare or non-rare based on their relative cover at a site. We compare our results presented in Figure 5 (column 1 with Cut off 1) to two additional cut offs for classifying a rare versus non-rare species (in columns 2 and 3); these cutoffs are described in Section *S8ciii.* Again, we estimate Equation (S1) with species richness disaggregated into the numbers of non-rare native species, rare native species, non-rare non-native species, and rare non-native species with groups defined based on each of the three cut offs. All estimated effects of each category of species richness (SR) are on a log-inverse-hyperbolic-sine scale. Given the inverse hyperbolic sine transformation of the species richness variables, the estimated effects cannot be interpreted as elasticities without further manipulation, but their signs and relative magnitudes can be compared to each other. Clustered-robust standard errors are used and clustered at the plot level. To see 95% confidence intervals as well, see the project page (output/TableS15_R_CI.tex).

	**Cut off 1 [Main Text]**	**Cut off 2**	**Cut off 3**
Non-rare, Native SR	0.0488	0.0505	0.0097
	(0.0721)	(0.0848)	(0.0717)
Non-Rare, Non-Native SR	-0.1721***	-0.2143***	-0.1746***
	(0.0649)	(0.0736)	(0.0664)
Rare, Non-Native SR	0.0397	-0.0178	-0.0254
	(0.0711)	(0.0658)	(0.0691)
Rare, Native SR	-0.1473 ***	-0.1050**	-0.0889*
	(0.0459)	(0.0445)	(0.0483)
Num. obs.	1,175	1,175	1,175
Num. plots	146	146	146
R^2 (full model)	0.79	0.78	0.78

S*ignif. Codes*: ***: 0.01, **: 0.05, *:0.1; Robust Standard errors in parentheses (clustered at plot level).

S8d. Variation in each species group

Our analysis exploits observed temporal variation in the data, namely year-to-year changes in each species richness group in a plot. To provide more contextual detail on this variation, we show how changes in the richness of each group of species varies over time by site in Figure S12. Figure S13 breaks this variation down even further by site. These figures imply that the

variation in overall species richness in Figure S2 is driven by changes in rare, native species and non-rare, non-native species.

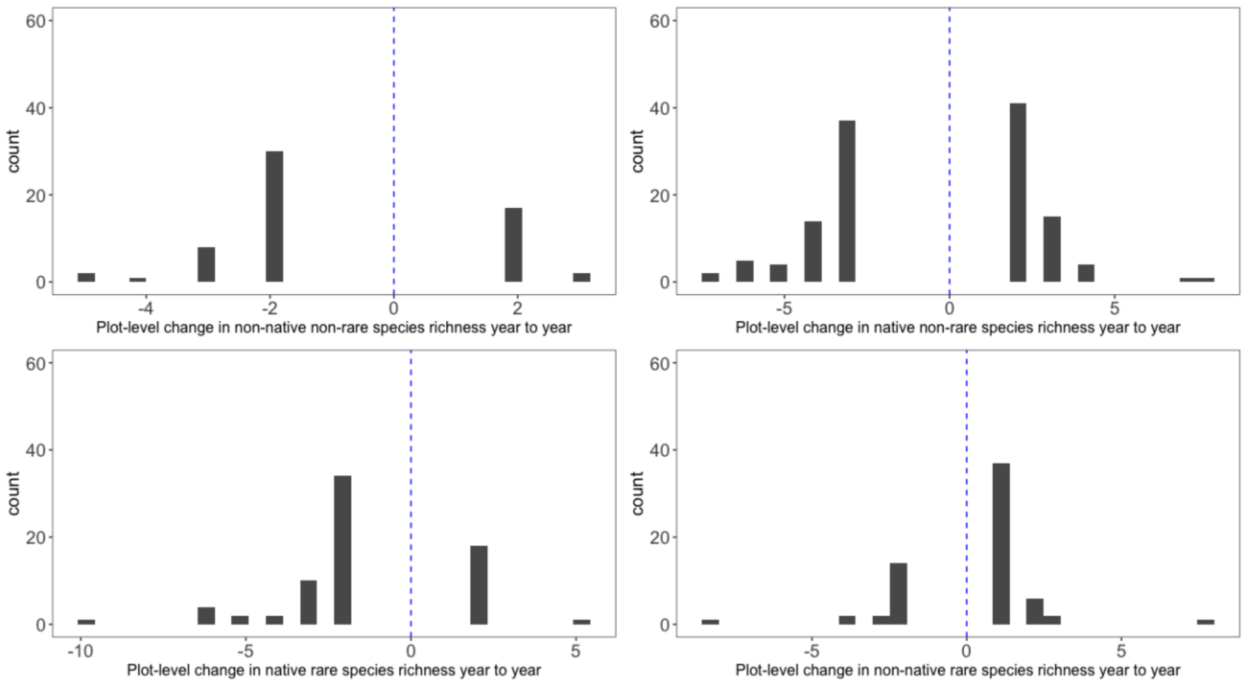

Figure S12. Year-to-year changes in the counts of each species group per plot. (A) non-native, rare species richness; (B) native, non-rare species richness; (C) native, rare species richness; and (D) non-native rare species richness.

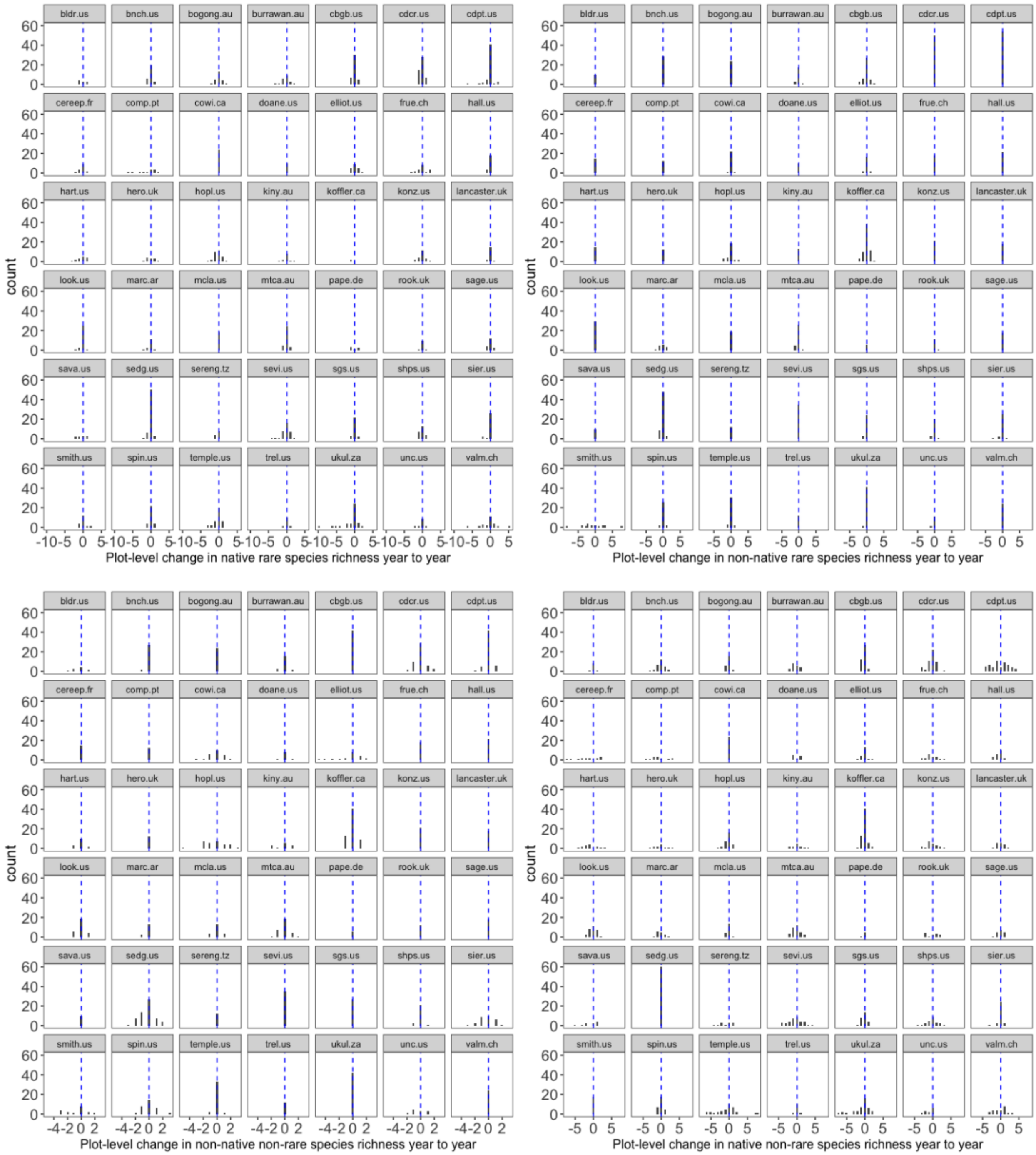

Figure S13. Year-to-year changes in the counts of each species group per plot for each site in the analysis.

Supplementary Discussion

S9. Nine Frequently Asked Questions (FAQ) about Dee et al.

An FAQ is not usually added to an SI, but we found this FAQ to be an effective way to answer a set of common questions that many readers have and to further clarify how our study differs from prior studies in ecology.

FAQ#1. Does Dee et al. overturn the popular wisdom from over thirty years of research on the effect of species richness on productivity?

No, but we hope our study gives ecologists a different perspective on the popular wisdom, a new approach for conducting empirical ecological research, and fruitful avenues to pursue in theory and experiments.

Regarding the new perspective on the popular wisdom, we believe our study:

(i) Estimates the average effect of changes in species richness on productivity when richness changes as it does in natural ecosystems, as opposed to when it changes via manipulations in experimental systems (see FAQs #2-#4)

(ii) Accounts for the biological complexity of the ecosystem more comprehensively than prior observational designs (see FAQs #6-#8).

Our results are, in fact, broadly consistent with experimental findings of biodiversity manipulations ('BEF experiments' hereafter). We estimate that an increase in the richness of species typically planted in BEF experiments – i.e., native, relatively common ('non-rare') species – has a positive effect on aboveground productivity. However, the Nutrient Network plots contain many more species than are, and can be feasibly, manipulated in experiments; namely many more rare and non-native species. More rare species and non-native species are associated with higher species richness (Fig. S10), and most species in these ecosystems are rare (Fig. S11) as in most ecosystems (*71*). These are the species that are changing the most from year to year (Fig. S12 & S13), and they have a different estimated average effect on productivity than do the native non-rare species (Figure 5, main text).

Regarding the new approach for ecological research, we believe our approach:

(iii) Makes our causal <u>aspirations</u> transparent ("*Exactly what ecological relationship are we trying to estimate?*"). See FAQ #2, #3, and #6.

(iv) Makes our causal assumptions transparent and motivates them through a combination of field knowledge and ecological theory ("*What exactly are we assuming when we give a causal interpretation to an estimated correlation between richness and productivity?*"). See FAQ#4, #5, #7, and #8.

(v) Assesses how changes in our causal assumptions affect our inferences ("*How would our interpretations change if we use alternative assumptions that may be plausible or equally as valid as the assumptions we originally made?*"). See FAQ #7 and Figure 3 in manuscript.

Our goal was to build on prior work and advance our collective understanding of the role of biodiversity in ecological functions – not to claim a 'final answer.' We make our assumptions about relationships between the data and our inferences as transparent as possible and explore the implications of these assumptions. This transparency, we hope, makes it easier for ecologists to continue to build on this work, by probing and relaxing these assumptions, and assessing their robustness to new data and methods.

FAQ#2. Why is the estimated average effect in Dee et al. negative while most experimental evidence implies the effect is positive? Is it because the research questions differ; in other words, because the causal effect that experiments aim to estimate and the causal effect that Dee et al. aim to estimate differ?

Yes, differences in research questions could be one reason why the signs of the estimated effects of species richness on productivity are different. Species richness could change in many ways, and these different ways are unlikely to have the same effects on ecosystem function.

The typical BEF experiment aims to estimate the expected effect from a change in richness that arises from a random draw from the pool of species that could grow at the study location and from the potential values of evenness (i.e., the effect of changing richness independent of composition attributes). That causal effect is helpful for developing theory because it isolates the effect of species richness separate from changes in species identities and evenness that may normally accompany a change in richness.

However, for understanding the ecology of natural systems and processes, particularly when thinking about the conservation implications of anthropogenic change, we and others (e.g., (*77*))

argue that the most relevant causal effect of richness on productivity is the expected effect of a change in species richness that mimics how richness changes in naturally occurring systems – more precisely, a change in richness that arises from a random draw from a weighted conditional distribution of species richness compositions at different richness values. For example, if dominant species tend to comprise the majority of species additions or subtractions when diversity is low, but rare species tend to comprise the majority of species additions or subtractions when diversity is high, changes in richness in the study should reflect those probability distributions (i.e., as a plot or site gets more diverse, the marginal/incremental species should be more likely to be rare). Our study aims to estimate this causal effect.

So, in sum, "Yes," the different research aims of the experimental and Dee et al. designs could be a reason for the divergence in results (see also **Box 1**).

> After reading this FAQ answer, are you thinking that "composition" is a confounder in Dee et al.? If yes, see **Figure 4** in main text.

FAQ#3. Why is the estimated average effect in Dee et al. negative while most experimental evidence implies the effect is positive? Is it because the effects of richness on productivity is conditional on species identity (a heterogeneous treatment) and the experiments do not plant the same set of species that are found in natural ecosystems?

Yes, the estimated effects of species richness on productivity may differ because the set of species growing in Dee et al. and in experimental studies differ.

To estimate the average effect of richness on productivity independent of other attributes of diversity, experiments would have to randomize *all* other attributes of diversity (e.g., identities,

Box 1: Spatial and temporal dimensions of diversity's effects on productivity

The causal effects described in FAQ#2 are not sufficiently precise because they lack spatial and temporal dimensions. *Over what spatial scale and time horizon are we evaluating the effect of a change in richness*? For example, are we referring to the difference in productivity in a patch over one year when the species richness changes from X to Y in a manner that mimics naturally occurring processes? Or are we referring to the difference in productivity in a patch over one hundred years when the species richness changes from X to Y in a manner that mimics naturally occurring processes? **We return to this issue in FAQ #5.**

densities, relative abundances, traits/functional characteristics). Of course, to do so would be prohibitively expensive or logistically infeasible. Instead, to our knowledge, most biodiversity-ecosystem functioning (BEF) experiments hold *planted* evenness *constant* at 1, with some exceptions (for instance, an experiment by Wilsey & Polley (2004) randomize richness across two values of evenness). Some experiments, for a subset of treatments, consider crosses between functional and species diversity (*78*). But it's logistically impossible to do all combinations. Moreover, BEF experiments plant some combinations of species from the set of all possible species identities and compositions that grow at a site, but not all. For example, in some places, sourcing seeds for rare species is prohibitively expensive. To our knowledge, BEF experiments do not plant all combinations of species identities, particularly at higher richness values.

In other words, the species planted in the experimental designs do not comprise the full set of species that may naturally grow at a study site. Recall that in Dee et al., the negative estimated effect of richness on productivity appears to come from rare species and non-native species. For native, dominant species, the estimated effect of richness on productivity is positive. This latter result from Dee et al result is consistent with BEF experiments, because these native, dominant species typically comprise most species planted in experiments. Few experiments plant truly rare species or non-native species, or in the proportions found in natural systems. We further test this conjecture by analyzing two long-term biodiversity experiments with our study design and find evidence consistent with the conjecture (see *Section S10* for more information).

In the few experiments that have planted rare species, the rare species are a small fraction of the total species that have been planted in nearly all cases (*but see* the Jena experiment (*79*)). In the few experiments that have planted non-native species, the non-native species are also a small fraction of the total species that have been planted. For example, BioCon includes two non-native species out of the 16 planted, and these two were naturalized to the site (P. Reich, *pers comm*). An exception is Wilsey *et al.* (2009), which paired species according to their native vs introduced status at a site in Texas, USA (*80*), where a maximum of 9 non-native species were planted, out of 20 total planted species.

Even in BEF experiments that plant rare species, the planted rare species may have been less likely to emerge and persist. This phenomenon is known as 'non-compliance' with treatment assignment.[3] With non-compliance, *Z* species are planted in a plot (planted richness), but only *D*

[3] Non-compliance is also a common, and frequently discussed, challenge in medical randomized control trials.

$< Z$ species emerge or persist (i.e., the 'realized richness') and subsequently affect productivity. If the species that have a negative or zero effect on productivity are less likely to grow or survive, it is possible that the true average effect of species richness on productivity in the experiments could be zero or negative ("true effect" meaning the effect if all planted species were to survive), while the "as planted" estimated average effect of richness on productivity reported by the experimenters is positive. Analyzing an example of experimental data, we find evidence for differential non-compliance – some species (e.g., *Anemone cylindrica*) that are rare in natural systems in Minnesota, USA do not frequently emerge in the experiment when planted, particularly in high diversity plots (**Box 2**). To determine relative abundance and rarity in natural communities, we use long-term survey data of grasslands around Minnesota, USA from the Minnesota Department of Natural Resources (MN DNR); see dataset details in (*81*).

Box 2. Differential non-compliance in experiments

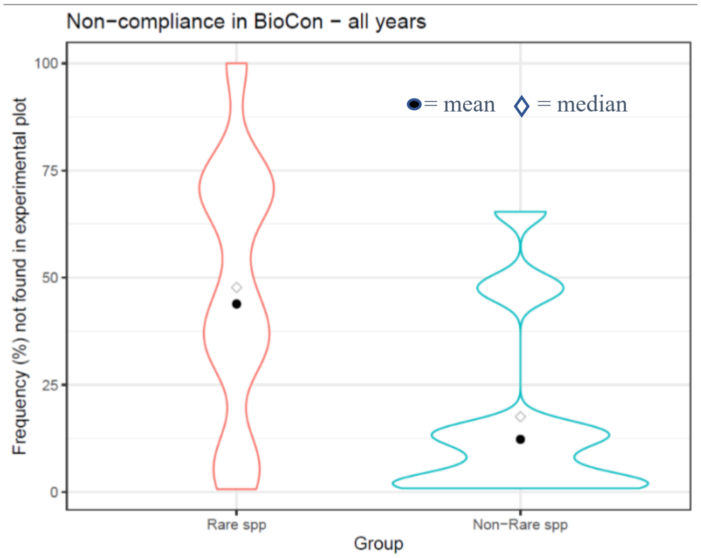

Are rare species less likely than other species to be observed growing after being planted in BEF experiments? To shed light on this question, we first analyze relative cover and relative frequency data from MN DNR's grassland monitoring. These data comprise over 41,000 observations of species, sampled over the last 10 years from 701 transects (each with ~25 plots). Using these data, we then classify species from BioCon experiments (Cedar Creek) as naturally rare or non-rare (dominant or subordinate) based on the metrics and cut-offs described in the Dee et al SI (section S8). Then we calculate the probability that a species is observed growing at each planted diversity level (1,2,4,8,16) by comparing the planted versus realized species data. We can then assess if "non-compliance" varies based on the species classifications from the MN DNR data (rare, subordinate, dominant). *We find that rare species are less likely be observed post-planting. Some rare species almost never emerge in low diversity plots and never emerge in the 16-species plots (e.g., Anemone cylindrica).*

So, in contrast to the causal effect that experiments aim to estimate (described in FAQ #2), the *causal effect that experiments actually estimate is the expected effect of a change in richness that arises from a random draw from a <u>subset of species</u> that could grow at the site, while holding evenness to a specific value*. These causal effects from different subsets, or versions, of richness can be helpful for shedding light on the mechanisms through which biodiversity affects ecosystem functions -- and thus for shedding light on the heterogeneity of biodiversity's effects on ecosystem functions.[4] But they may not match, in sign or magnitude, the target causal effect that experimentalists aim to estimate.

In sum, "Yes," the results from experimental designs and Dee et al.'s design may differ because different species are planted or survive in the two designs.

FAQ#4. Why is the estimated average effect in Dee et al. negative while most experimental evidence implies the effect is positive? Is it because of potential statistical biases in Dee et al., like unobserved confounding variables or reverse causality, or potential statistical biases in experimental designs?

Yes, biases in either experimental designs or in Dee et al.'s design could create the divergence in results. In our study, we highlight a variety of potential sources of hidden bias in their design: for example, unobserved confounders, including measurement error, and reverse causality, where the effect of productivity on diversity masks or mimics the effect of diversity on productivity. We attempt to shed light on whether these sources of potential bias could plausibly mask a true positive relationship between richness and productivity. We find no evidence for such masking, but, of course, absence of evidence is not evidence of absence. Note that the estimation method itself (i.e., the statistical model) is not likely to yield a negative effect when the true effect is positive. Indeed, applying our estimation method to experimental data (with a time-varying treatment or before-after data) gives the same answer as a simpler method that simply compares mean differences among treated and control groups (section S10).

Experimental designs with randomized treatments can also have hidden biases (reviewed in (*82*)). For example, a design that uses comparisons of productivity across different levels of planted richness to estimate richness's effect on productivity could be biased if randomized planting, followed by weeding of non-planted species, affects productivity through channels

[4] However, a broader suite of mechanisms could be operative in natural systems or the relative importance of the mechanisms could differ (*92*).

other than richness (e.g., a channel like soil disturbance). Wilsey & Polley (2006) noted that "manipulative experiments have the disadvantage of disturbing soil during plot establishment (*84*)."

Although hidden biases in one or both types of designs (ours and experimental) could be one reason for the divergence in results, we believe the two reasons outlined in FAQ #2 and FAQ #3 are more likely: different research questions and different sets of species.

FAQ#5. Models and causal inference: could we use structural equation modeling?

The aim of causal inference is to move from a statistical model to a causal model – or more precisely, to move from a statistical interpretation of a model to a causal interpretation. One cannot make that move without making assumptions, some of which are likely to be untestable. In other words, causal inference cannot rely simply on statistical methods (e.g., structural equation modeling and t-tests). Methods must be complemented by assumptions that determine whether a relationship estimated within a model can be interpreted causally (*5*, *8*, *9*). Assumptions are required for all casual inference, whether the data come from an experimental design or an observational design.

These causal assumptions typically matter more than the statistical methods – an insight that is often summarized in variants of the phrase "design matters more than methods." For example, both structural equation modeling (SEM) and our approach use regression models. In fact, the Dee et al. design could be implemented within an SEM framework. On their own, regression models are simply statistical models without any causal content.

The key innovation of our study is the *design* rather than the estimation method. In other words, our key contribution is the insight that panel data can be exploited to control for a wider range of confounding variables than prior studies have achieved. We exert this control in two ways: (1) control for plot-level fixed effects via a deviation-in-means statistical estimation procedure; and (2) adding site-by-year dummies to our regression estimator. We could have exerted this control in multi-level or SEM model by (1) controlling for plot-level fixed effects via a centering transformation of the data; and (2) adding site-by-year dummies to a SEM model. Like any causal study, a study that uses SEM requires assumptions for causal interpretation of its estimates. For SEMs that do not use panel data, a large set of assumptions must be satisfied for each equation in a SEM to be able to estimate, without bias, its target causal effects. For

example, a SEM with a single year of data cannot address potential confounders for each of the target causal variables unless either: (1) all potential confounders are measured and in the model; or (2) the SEM has a valid instrument variable (IV) for each causal variable of interest (variable *Z* in Fig. 1). In other fields, scholars view these assumptions as hard to defend and thus there is some skepticism about such "all-cause" models (with multiple hypothesis tests, one also needs to maintain the family-wise Type 1 error rate or control the false discovery rate).

FAQs #6 and #7. Readers of earlier versions of our manuscript have asked us to explain how it is possible that Dee et al. estimate a negative average effect of plot-level richness on productivity while Grace et al. (2016, *Nature*) estimate a positive effect, even though both studies use unmanipulated plots from the Nutrient Network.

The Grace et al. study was a seminal study because it took seriously the complexity of the biological system and used a multivariate approach to quantify relationships among different variables. As noted in the main text, we build on their study and others that followed. Although we cannot know with certainty why the results from our study differ, we briefly summarize our intuition in the main text and, in this FAQ, we describe in more detail the most plausible reasons for the differences.

As noted in FAQ#6, Dee et al. builds on the multivariate advance of Grace et al. Both studies are based on strong theory and field experience about the biology of the systems being studied. Although Dee et al.'s design may look, on the surface, to be less complex than Grace et al.'s SEM, the Dee et al. model tries to address the same ecological complexity. Both designs pose biologically informed hypotheses. It is true that the Grace et al. design aims to test many more hypotheses, i.e., it aims to estimate many more causal relationships than Dee et al. try to estimate (at least 15, by our count). Dee et al. chose to narrow the set of research questions to focus on ruling out rival explanations that arise from spatial and temporal biological complexity in grassland ecosystems. Thus, the key distinction between Grace et al and Dee et al is in the *designs* – not the methods.

FAQ#6. Why is the estimated average effect of plot richness on plot productivity in Dee et al. negative while in the Grace et al. study it is positive? Is it because the causal effect that the studies aim to estimate differs?

No, the target causal effect in Dee et al. is also one of the target causal effects in Grace et al. Grace et al. try to estimate more causal effects than Dee et al., but both studies aim to estimate the average effect of a change in plot-level richness on plot-level productivity. It is true that Grace et al. and Dee et al. measure productivity differently. Grace et al. measure it as total biomass (sum of live and dead biomass), whereas Dee et al., and many other studies, measure it as live biomass. But using total biomass, Dee et al. generate the nearly same estimated negative effect as they do with live biomass (-0.24, CI [-0.37, -0.11]). See code on project page.

Aren't the "changes in species richness" in Grace et al., which uses spatial variation in richness to estimate richness's effect on productivity, different from the "changes in species richness" in Dee et al., which uses temporal variation in richness? More specifically, doesn't Grace et al. measure a "long-run effect" of richness on productivity, while Dee et al. measure a "short-run effect?"

We believe this question, which we have received from many people, requires some clarification and elaboration about the two study analysis designs. So, we break down our answer in two parts. First, we clarify that *both* designs use spatial variation, but only Dee et al. uses *both spatial and temporal* variation. Second, we clarify when and whether comparing productivity across plots with different values of richness, as the comparison is done in Grace et al., can provide insights into a "long-run effect" of richness on productivity.

Both studies use spatial variation, but each uses this variation differently.

Our analysis in Dee et al. uses **both** *spatial variation across sites and plots* and *temporal variation across both sites and plots*. In Grace et al., the authors use spatial variation *across sites and plots*; temporal variation is not used because there is only one year of data per plot.

The Dee et al. model eliminates the spatial variation that comes from the "between-plots" comparisons because we believe those comparisons will yield biased inferences about the relationship between richness and productivity – hidden bias that comes from unobserved confounding variables (*see our accompanying Rmarkdown tutorial for elaboration and a visual*).

Yet, even if we were to ignore the potential bias from the between-plots comparisons and use information from both within and between-plot comparisons and within and between-site comparisons (called *random effects*" estimator in economics, (*17*)), we would still get a negative estimated effect: -0.19 (SE=0.07, p= 0.01, 95% CI [-0.33, -0.04]). Leveraging temporal variation across sites and plots is a key innovation.

So, the negative estimated effect in Dee et al. does not arise because their design does not use the between-plot, spatial variation in richness.

Can focusing on spatial variation across plots and sites yield insights into the "long-run effect" of richness on productivity?

By "long-run effect," we mean the effect on productivity of a more permanent, or persistent, shift in richness that harnesses long-run mechanisms driven by processes like speciation and evolutionary history occurring over long periods of time -- e.g., *does greater diversity explain why certain ecosystems are more productive than others, holding all other factors constant*?

In Dee et al., the estimated effect in the main design is a short-run effect: the effect of a change of richness on productivity within ecosystems in a year. We rely on the annual within-plot changes in richness because this variation allows us to control for unobserved plot-level and site-level confounders that are not easily controlled for in the Grace et al. design with its single year of data. The annual changes in species richness also allows us to control for reverse causality in two ways that differ from Grace et al.'s approach (see FAQ#7). We believe that estimates of the short-run effects of changes in richness on productivity are ecologically relevant because they capture the effects of changes in today's ecosystems. Nevertheless, using short-term variation in richness may not provide insights into the effects of more persistent shifts in richness over long time periods.

We next explain and explore considerations for estimating a long-run effect. First, we address this question: "If we only use spatial variation in richness across plots, and ignore the within-plot temporal variation, could we infer the long-run effects of richness on productivity?" To do so, we apply a between-plot estimator to our data. We still obtain an estimate that is negative, albeit small and imprecisely estimated because we only have 2 or 3 plots per site (see STATA code on the release of the project page).

The challenge of estimating long-run effects without long-run data is not unique to ecology – it's a challenge in all empirical science. An important example is the debate over the effects of climate change, a long-run phenomenon, versus the effects of weather, a short-run phenomenon. As in the diversity-productivity context, when there are no data at the temporal scale that one seeks to estimate an effect, one is stuck either (a) drawing inferences about long-run effects (climate change, persistent changes in biodiversity) by making strong, and hard to justify, assumptions about the data-generating process, or (b) drawing inferences about short-run effects (weather, short-run changes in biodiversity) by making more credible assumptions, which come at the cost of less certainty over the generalizability of the estimated effects to longer time scales. For these reasons, we opt to estimate the short-run effect.

FAQ#7. Why is the estimated average effect in Dee et al. negative while in the Grace et al. study it is positive? Is it because each study makes different assumptions about what is driving changes in richness and productivity?

Yes, we believe this reason is the main reason for the different results. The key insight from Dee et al is that imposing different causal assumptions leads to different causal models, and different models can yield different conclusions. If the underlying assumptions were false, the estimated correlation between richness and productivity may not reflect a causal relationship.

Here we try to contrast the assumptions being made in the two studies – our main design (Figure 2) and the Grace et al design. In doing so, we are not criticizing the Grace et al. design, but rather showing how their assumptions differ and thus can lead to different conclusions. In this FAQ, we only review the assumptions made in the Dee et al. main design. One strength of our study is that we also use other designs that require different assumptions for drawing casual inferences from the data (Figure 3). Through those designs, we probe the robustness of our results to violations in the assumptions in the main design. The alternative assumptions in these complementary analyses are described in detail in the SI below (section S5).

Grace et al. assumptions

To estimate the effect of plot-level richness on plot-level productivity using the Grace et al. design, one must assume the following:

G1. The plot-level "soil suitability" variable, which is a weighted combination of the percent silt and percent sand, only affects plot productivity via its effect on plot richness; i.e., soil

suitability is correlated with richness but uncorrelated with the error term in the plot productivity equation, after conditioning on site productivity. The key insight in Grace et al. design is that, to estimate the effect of plot richness on plot productivity without bias (i.e., to control for omitted variables and for reverse causality), one needs a variable that is correlated with richness, but not correlated with productivity, except through its correlation with richness; a so-called "**instrumental variable**" (IV). If, however, soil suitability affects productivity through channels other than species richness (after conditioning on other variables in the model), the estimated correlation between richness and productivity may not reflect a causal relationship.

a. If assumption G1 were wrong, the SEM in Grace et al. can still control for confounders (but not reverse causality) if the only confounders are the three observable variables in the plot productivity equation, or if one were willing to make a very strong assumption about the covariance between unobserved richness and productivity shocks (i.e., confounders).

G2. There is no non-classical measurement error[5] in any of the model variables that creates bias in the design. Measurement error could come from either field measurements of the variables in the model or the imputed missing soil variable values. For example, one must assume:

a. No measurement error in the observable control variables in the regression that are also assumed to be correlated with richness (e.g., soil suitability).
b. No measurement error in productivity that is correlated with richness.

G3. Implicit in the design are also assumptions about the nature of heterogeneous causal effects (i.e., the variability across plots in the effect on productivity from changing richness from *X* to *Y* species) and how the moderators of those heterogeneous effects are distributed across plots. Because those assumptions are more technical, we do not discuss them in detail here, but they are described in (*85*).

In summary, Grace et al. recognize that, in a design with only one year of data, control for unobserved confounders is challenging unless one has a valid IV. A valid IV also controls for

[5] Classical measurement error in a variable is when the variable is measured with error but that error is independent of the variables value. Non-classical measurement error is when measurement error of the variable is correlated with the variables value.

reverse causality. There is no empirical test that can validate the assumption that there is no correlation between an IV (soil suitability) and the outcome variable (productivity) except through the causal variable (richness). One must use theory and field experience to assess its validity. Other implicit assumptions made in using SEMs can be found in (*86, 87*).

Dee et al. assumptions

With data that varies over space and time, the challenges to inferring causality from observational data are less formidable than the challenges when using data that only varies across space. Using panel data, Dee et al. address bias from confounding variables (omitted variable bias) and bias from reverse causality in multiple and separate ways (see Fig 2 & 3 in manuscript). The assumptions used in the Main Design are in the manuscript and SI, but we summarize them here (other implicit assumptions in our main design are reviewed in (*17, 88*)):

D1. No plot-level confounders that vary over time. We try several approaches to assess the robustness of results to this assumption and also use alternative designs that do not require this assumption (see Figure 3 and section S7):

a. We test the sensitivity of their inferences to changes in this assumption.
b. We use an alternative specification that controls for time-varying, plot-level confounders that are correlated with plot productivity, lagged one year.
c. To address reverse causality, we use an instrumental variable (IV) design (a different IV from the IV used in Grace et al; see assumption D3). This design also controls for time-varying plot-level confounders when the IV is valid.

D2. No non-classical measurement error that would create bias in the design (*89*). The only "control variables" are site and year, and thus one may reasonably assume that these variables are measured without error. Thus, the only measurement error of concern is error in productivity measures that are correlated with richness (or correlated with the predictors of richness in the instrumental variable design).

D3. In the IV design, the species richness of neighboring plots (the experimentally manipulated plots) only affects plot productivity via its effect on plot species richness; i.e., richness of neighbors is correlated with own richness but uncorrelated with the error (disturbance) term in the own plot productivity equation after conditioning on time-invariant plot attributes and time-varying site attributes. To control for reverse causality, another analysis makes a different set of assumptions than the IV design. We call this the "mechanism blocking" in

Figure 3, and it assumes that one important mechanism through which productivity affects richness is shading (a proposed mechanism from Grace et al.)

D4. A weaker version of assumption G3 from the Grace et al design (weaker because we do not rely on a large set of plot or site-level covariates in our estimation procedure).

No empirical test can validate these four assumptions. One must use theory and field experience to assess their validity and one must probe the robustness of the results to potential violations in the assumptions. To probe the robustness of our results, we use four approaches (Fig. 3). In each approach, like our Main Design, causal inference requires causal assumptions. But each approach makes different assumptions. In other words, each approach can detect hidden biases in our design (i.e., threats to internal validity) under different conditions. Although each analysis uses different causal assumptions, each comes to the same conclusion. That pattern is the source of our paper’s strength of evidence: the results of each individual analysis could be explained by a different rival explanation, but it is harder to come up with a coherent set of rival explanations that could explain them all (including the results in Fig. 5).

Why would these different assumptions lead to estimated effects of opposite sign?

Here, we elaborate on the logic that underlies the progression of analyses in the Results section of the main text. This progression also highlights how our study builds on prior studies. Let’s start by assuming that the data-generating process in the Nutrient Network grassland ecosystems is such that the true average effect of an increase in species richness on live biomass is negative (perhaps small).

Could the three designs generate different conclusions from the same data set? Before using Nutrient Network data to illustrate how the designs can indeed generate different conclusions, we explain the intuition via four arguments:

1. In an observational design using Nutrient Network data, there are plot and site attributes that affect richness and productivity in the same directions and that affect richness and productivity in opposite directions (e.g., nitrogen positively affects productivity and negatively affects richness). If these positive and negative confounding forces are roughly similar in magnitude, the simple bivariate correlation between richness and productivity

would be close to zero and thus would be difficult to detect statistically. The estimated correlation may look weakly positive or negative, depending on the sample.

2. The plot and site-level confounders that are typically measured in ecological field work tend to move richness and productivity in opposite directions, on average (e.g., nitrogen negatively affects richness and positively affects productivity). Controlling for only these variables thus yields a positive estimated correlation between richness and productivity.
3. The plot and site-level confounders that are typically not collected in ecological field work tend to move richness and productivity in the same directions, on average (e.g., growing season precipitation). If one could observe these variables and only controlled for these variables, the estimated correlation between richness and productivity would be negative and much larger than the true negative effect.
4. Controlling for both the observed and unobserved sources of bias leads to a negative estimated effect of species richness on productivity, but one that is smaller than the estimate from (3). Arguments (1)-(3) reflect what is sometimes called “point-by-point bias,” which arises when one controls for some sources of statistical bias but not others. “Point-by-point bias” results in the estimated effect moving further from the true effect in comparison to when there are no controls for statistical bias.

Now, using the Nutrient Network data, we present three analyses that yield patterns consistent with the four arguments:

A. *Bivariate Correlation between Richness and Productivity*: If we were to do an Adler et al-like analysis (controlling for year, given we have multiple years), we obtain an Adler et al-like result: the estimated correlation is positive (b = 0.14) but we cannot reject the null hypothesis that the correlation between richness and productivity is zero (p = 0.17). If we just used data from a single year, we find a positive, but statistically insignificant, estimated effect in some years and a negative, but statistically insignificant, effect in other years.

B. *Multivariate Model that Controls for Observed Confounders*: Multivariate models not only have higher explanatory power than traditional bivariate analyses, they also can control for confounders when the confounding variables are observable. In Grace et al., a multivariate model has much higher explanatory power than the bivariate model and yields a larger, positive effect of richness on productivity than the bivariate model. Here, we construct a multivariate model that yields similar results. We assume that we do not have a valid IV,

but we can try to control for many potential confounders using Nutrient Network data. If we were to build on the bivariate analysis via a multivariate analysis that controls just for 17 soil variables, we obtain a result consistent with prior multivariate analyses: a positive estimated effect (b=0.28) for which one can reject the null hypothesis of the correlation being equal to zero (p=0.04). Another similar result: in contrast to the low R^2 in the bivariate analysis (0.04), the R^2 of multivariate analysis is much higher (0.39). If we add to the model more possible confounders like weather, country, habitat type, and prior use (60 variables in total), the estimated effect is larger (b=0.38; p=0.02) and the R^2 is 0.55 (that's an overall R^2 for variation both within and between plots; the between-plot R^2 is 0.95).

C. *Multivariate Model that Controls for a Wider Range of Observed and Unobserved Confounders (the "Common Design in Ecology" presented in this paper)*: Drawing on our understanding of the biological complexity of these grassland systems, we know that there are *many* unmeasured variables that could affect both biodiversity and productivity and thus could be confounding the relationship between richness and productivity; e.g., land-use intensity and history (e.g., (*90*)), grazing intensity, pollinator diversity at the site, drought or extreme precipitation events, annual growing season start time, *etc.* Even adjusting for 60 covariates, as in (B), is unlikely to eliminate all confounding effects. Because we have data over both space and time, we can control for a wide range of confounders, whether the confounding variables can be observed or not. For this reason, we build on the multivariate analysis in (B) by first controlling for site-level confounders via site-by-year variables in the model. The estimated correlation between richness and productivity is of a similar magnitude to the estimate in (B), but negative (b=-0.21). When we further control for plot-level confounders, we get the estimated negative effect in Dee et al. (b = -0.24).

What's happening? The Nutrient Network sites are likely to experience site-specific "shocks" that vary each year (e.g., weather shocks, like a particularly dry April, or herbivory shocks, like higher herbivore pressure than the prior year). We don't know what exactly these shocks are, but because we observe the same sites over many years, we can control for them. In our data, we observe that these shocks affect productivity and richness in the same direction, on average. We also observe that many of the observable variables collected by Nutrient Network researchers affect productivity and richness in opposite directions, on average. So, as noted by

Grace et al., when one looks at a single year of data, the bivariate correlation will not show anything of note. If one does not have a valid IV, then when one controls for observable confounders (like soil attributes), one will see a positive correlation between richness and productivity. However, when one controls for a much larger range of sources of positive and negative bias, as done in Dee et al., you see an overall negative average effect.

So, "Yes," we believe that some or all the difference in results between Dee et al. and Grace et al. is attributable to the studies making different assumptions. Which, if any, of these sets of assumptions better approximates the truth is something a reader must decide. Future research could focus on assessing the ecological conditions under which each set of assumptions may be plausible.

FAQ#8. Why is the estimated average effect in Dee et al. negative while in the Grace et al. study it is positive? Is it because Grace et al. have a larger data set?

No, both studies have large data sets. Both studies have roughly the same number of sites: 43 in Dee et al. vs 39 in Grace et al.). Grace et al. has more plots, whereas Dee et al. has more years. Given that the unmanipulated plots in Nutrient Network are a random sample of plots from the sites, the two samples have similar expected values for plot and site attributes ("similar" because the sets of sites are not identical). In other words, the external validity of the two data sets is roughly the same, with Dee et al. perhaps having an advantage with more sites and more years. Moreover, neither study suffers from low statistical power (**Box 3**).

Box 3: Statistical power

Dee et al. have two or three plots per 43 sites, on average, with at least five years of data per plot. Grace et al. have larger number of plots from 39 sites from a single year. Grace et al.'s larger number of plots helps increase the statistical power of their between-plot design. But Dee et al. have five or more time periods per plot and that helps to increase the statistical power of their within-plot design. The Dee et al. design is underpowered if, like Grace et al., we were to rely only on the between-plot variation in richness. The Grace et al. design is underpowered if, like Dee et al., they were to try to control for all site differences with site-level dummy variables.

FAQ#9. Won't the answer always be that the effect of species richness on productivity 'depends'?

Yes and no. Although the sign and strength of the relationship between richness and productivity will likely vary with context, the average effect of changes in species richness on productivity in an ecosystem is relevant both for science and for practice. Nevertheless, we acknowledge that elucidating how the relationship between richness and productivity varies is important. In our study, we make some advances by exploring two sources of heterogeneity: compositional variations in the construct "richness" (i.e., multiple versions of richness depending on composition) and variations in site and plot-level moderators (i.e., attributes that are off the causal path between richness and productivity, like precipitation or clay content of soil, but which moderate the mechanism effects between richness and productivity). In other words, we explore the implications of both heterogeneous treatments and heterogeneous average causal responses (see (*91*) for more on these concepts).

Regarding richness as a heterogeneous treatment, we demonstrate that the effect of richness on productivity depends on what type of species are changing at a site. We focus on the heterogeneous effects of changes in rare species and dominant species, as well as changes in native and non-native species. But other species attributes may also matter.

Regarding heterogeneous average causal responses from variations in moderating site and plot conditions, we present some initial results on those moderators, but more work is needed (see *Section S6c & S6d*, Tables S3-S6). Estimating heterogeneous treatment effects is challenging because the probability of false positives grows dramatically as one explores various interaction effects. Investigating heterogeneity in a treatment effect can quickly become an exploratory, data-mining exercise in which we look for variation in the estimated effect of richness conditional on a wide range of site and plot attributes. Instead, we advocate for a focus to a test of a hypothesis implied by theory. Thus, more detailed exploration of heterogeneous treatment effects is beyond the scope of the Dee et al. study.

S10. Supplementary References

1. S. L. Morgan, C. Winship, *Counterfactuals and causal inference* (Cambridge University Press, 2015).

2. D. Rubin, Causal inference using potential outcomes: Design, Modeling, Decisions. *J. Am. Stat. Assoc.* **100**, 322–331 (2005).

3. J. D. Angrist, J. Pischke, *Mostly Harmless Econometrics: An Empiricist's Companion* (Princeton University Press, Princeton, NJ, 2009).

4. G. W. Imbens, J. M. Wooldridge, Recent Developments in the Econometrics of Program Evaluation. *J. Econ. Lit.* **47**, 5–86 (2009).

5. J. Pearl, *Causality: Models, reasoning, and inference, second edition* (2011).

6. D. B. Rubin, Estimating causal effects of treatments in randomized and nonrandomized studies. *J. Educ. Psychol.* **66** (1974), doi:10.1037/h0037350.

7. A. E. Larsen, K. Meng, B. E. Kendall, Causal Analysis in Control-Impact Ecological Studies with Observational Data. *Methods Ecol. Evol.*, 2041–210X.13190 (2019).

8. D. Rubin, Causal inference using potential outcomes: Design, Modeling, Decisions. *J. Am. Stat. Assoc.* **100**, 322–331 (2005).

9. J. Pearl, Causal inference in statistics: An overview. *Stat. Surv.* **3**, 96–146 (2009).

10. G. T. Smith, On Construct Validity: Issues of Method and Measurement. *Psychol. Assess.* **17**, 396–408 (2005).

11. M. E. Strauss, G. T. Smith, Construct validity: Advances in theory and methodology. *Annu. Rev. Clin. Psychol.* **5**, 1–25 (2009).

12. E. Everts, Identifying a particular family humor style: A sociolinguistic discourse analysis. *Humor*. **16**, 369–412 (2003).

13. P. J. Ferraro, M. M. Hanauer, Advances in Measuring the Environmental and Social Impacts of Environmental Programs. *Annu. Rev. Environ. Resour.* **39**, 495–517 (2014).

14. P. R. Armsworth, K. J. Gaston, N. D. Hanley, R. J. Ruffell, Contrasting approaches to

statistical regression in ecology and economics: FORUM. *J. Appl. Ecol.* **46** (2009), doi:10.1111/j.1365-2664.2009.01628.x.

15. V. Butsic, D. J. Lewis, V. C. Radeloff, M. Baumann, T. Kuemmerle, Quasi-experimental methods enable stronger inferences from observational data in ecology. *Basic Appl. Ecol.* **19** (2017), , doi:10.1016/j.baae.2017.01.005.

16. B. M. Bolker, M. E. Brooks, C. J. Clark, S. W. Geange, J. R. Poulsen, M. H. H. Stevens, J.-S. S. White, Generalized linear mixed models: a practical guide for ecology and evolution. *Trends Ecol. Evol.* **24**, 127–135 (2009).

17. J. M. Wooldridge, Econometric Analysis of Cross Section and Panel Data. *Booksgooglecom* (2002), doi:10.1515/humr.2003.021.

18. P. J. Ferraro, M. M. Hanauer, Advances in Measuring the Environmental and Social Impacts of Environmental Programs. *Annu. Rev. Environ. Resour.* **39**, 495–517 (2014).

19. M. A. Huston, Hidden treatments in ecological experiments: Re-evaluating the ecosystem function of biodiversity. *Oecologia*. **110**, 449–460 (1997).

20. G. W. Imbens, Instrumental Variables: An Econometrician's Perspective. *Stat. Sci.* **29**, 323–358 (2014).

21. J. D. Angrist, G. W. Imbens, D. B. Rubin, J. D. Angrist, G. W. Imbens, D. B. Rubin, Identification of Causal Effects Using Instrumental Variables Linked references are available on JSTOR for this article : Identification of Causal Effects Using Instrumental Variables. *J. Am. Stat. Assoc.* **91**, 444–455 (1996).

22. B. E. Kendall, A statistical symphony: Instrumental variables reveal causality and control measurement error. *Ecol. Stat. Contemp. Theory Appl.*, 149–167 (2015).

23. A. J. Macdonald, E. A. Mordecai, Erratum: Amazon deforestation drives malaria transmission, and malaria burden reduces forest clearing (Proceedings of the National Academy of Sciences of the United States of America (2019) 116 (22212-22218) DOI: 10.1073/pnas.1905315116). *Proc. Natl. Acad. Sci. U. S. A.* **117**, 20335 (2020).

24. A. J. MacDonald, A. E. Larsen, A. J. Plantinga, Missing the people for the trees:

Identifying coupled natural–human system feedbacks driving the ecology of Lyme disease. *J. Appl. Ecol.* **56**, 354–364 (2019).

25. H. S. Wauchope, T. Amano, J. Geldmann, A. Johnston, B. I. Simmons, W. J. Sutherland, J. P. G. Jones, Evaluating Impact Using Time-Series Data. *Trends Ecol. Evol.* **36** (2021), pp. 196–205.

26. J. Fieberg, M. Ditmer, Understanding the causes and consequences of animal movement: A cautionary note on fitting and interpreting regression models with time-dependent covariates. *Methods Ecol. Evol.* (2012), doi:10.1111/j.2041-210X.2012.00239.x.

27. D. R. Schoolmaster, J. B. Grace, E. W. Schweiger, B. R. Mitchell, G. R. Guntenspergen, A causal examination of the effects of confounding factors on multimetric indices. *Ecol. Indic.* (2013), doi:10.1016/j.ecolind.2013.01.015.

28. D. R. Schoolmaster, C. R. Zirbel, J. P. Cronin, A graphical causal model for resolving species identity effects and biodiversity–ecosystem function correlations. *Ecology* (2020), doi:10.1002/ecy.3070.

29. B. Shipley, *Cause and Correlation in Biology A User ' s Guide to Path Analysis , Structural Equations and Causal Inference* (2004).

30. J. B. Grace, K. M. Irvine, Scientist's guide to developing explanatory statistical models using causal analysis principles. *Ecology*. **101**, 1–14 (2020).

31. J. Pearl, The Causal Foundations of Structural Equation Modeling. *Handb. Struct. Equ. Model.* (2014).

32. E. T. Borer, W. S. Harpole, P. B. Adler, E. M. Lind, J. L. Orrock, E. W. Seabloom, M. D. Smith, Finding generality in ecology: A model for globally distributed experiments. *Methods Ecol. Evol.* **5**, 65–73 (2014).

33. E. T. Borer, J. B. Grace, W. S. Harpole, A. S. MacDougall, E. W. Seabloom, A decade of insights into grassland ecosystem responses to global environmental change. *Nat. Ecol. Evol.* **1**, 1–7 (2017).

34. J. B. Grace, T. M. Anderson, E. W. Seabloom, E. T. Borer, P. B. Adler, W. S. Harpole, Y.

Hautier, H. Hillebrand, E. M. Lind, M. Pärtel, J. D. Bakker, Y. M. Buckley, M. J. Crawley, E. I. Damschen, K. F. Davies, P. A. Fay, J. Firn, D. S. Gruner, S. M. Prober, M. D. Smith, Integrative modelling reveals mechanisms linking productivity and plant species richness. *Nature*. **529**, 390–393 (2016).

35. P. B. Adler, E. W. Seabloom, E. T. Borer, H. Hillebrand, Y. Hautier, A. Hector, W. S. Harpole, L. R. O. Halloran, J. B. Grace, T. M. Anderson, J. D. Bakker, L. a Biederman, C. S. Brown, Y. M. Buckley, L. B. Calabrese, C. Chu, E. E. Cleland, S. L. Collins, Productivity Is a Poor Predictor of. *Science (80-.)*. **1750**, 1750–1754 (2011).

36. J. E. Duffy, C. M. Godwin, B. J. Cardinale, Biodiversity effects in the wild are common and as strong as key drivers of productivity. *Nature*. **549**, 261–264 (2017).

37. B. J. Cardinale, K. L. Matulich, D. U. Hooper, J. E. Byrnes, E. Duffy, L. Gamfeldt, P. Balvanera, M. I. O'Connor, A. Gonzalez, The functional role of producer diversity in ecosystems. *Am. J. Bot.* **98**, 572–92 (2011).

38. F. van der Plas, Biodiversity and ecosystem functioning in naturally assembled communities. *Biol. Rev.* **94**, 1220–1245 (2019).

39. F. I. Isbell, D. Craven, J. Connolly, M. Loreau, B. Schmid, C. Beierkuhnlein, T. M. Bezemer, C. Bonin, H. Bruelheide, E. de Luca, A. Ebeling, J. N. Griffin, Q. Guo, Y. Hautier, A. Hector, A. Jentsch, J. Kreyling, V. Lanta, P. Manning, S. T. Meyer, A. S. Mori, S. Naeem, P. A. Niklaus, H. W. Polley, P. B. Reich, C. Roscher, E. W. Seabloom, M. D. Smith, M. P. Thakur, D. Tilman, B. F. Tracy, W. H. van der Putten, J. van Ruijven, A. Weigelt, W. W. Weisser, B. Wilsey, N. Eisenhauer, Biodiversity increases the resistance of ecosystem productivity to climate extremes. *Nature*. **526**, 574–577 (2015).

40. Lauenroth, W. K., H. W. Hunt, D. M. Switft, J. Singh, Estimating aboveground net primary production in grasslands: a simulation approach. *Ecol Model.* **33**, 297–314 (1986).

41. J. C. Moore, in *Encyclopedia of Biodiversity (Second Edition)*, S.A. Levin, Ed. (Elsevier, Second., 2013), pp. 648-656.

42. H. W. Jari Oksanen, F. Guillaume Blanchet, Michael Friendly, Roeland Kindt, Pierre

Legendre, Daniel McGlinn, Peter R. Minchin, R. B. O'Hara, Gavin L. Simpson, Peter Solymos, M. Henry H. Stevens, Eduard Szoecs, vegan: Community Ecology Package (2019).

43. M. Bertrand, E. Duflo, S. Mullainathan, How much should we trust differences-in-differences estimates? *Q. J. Econ.* (2004), , doi:10.1162/003355304772839588.

44. A. C. Cameron, D. L. Miller, A Practitioner's Guide to Cluster- Robust Inference. *J. Hum. Resouces*. **50**, 317–372 (2015).

45. B. Callaway, A. Goodman-Bacon, P. H. C. Sant'Anna, Difference-in-Differences with a Continuous Treatment (2021) (available at http://arxiv.org/abs/2107.02637).

46. S. Gaure, Lfe: Linear group fixed effects. *R J.* **5**, 104–116 (2013).

47. G. Sugihara, R. May, H. Ye, C. Hsieh, E. Deyle, M. Fogarty, S. Munch, Detecting Causality in Complex Ecosystems. *Science (80-.).* **338** (2012).

48. P. W. Holland, Statistics and causal inference. *J. Am. Stat. Assoc.* **81**, 945–960 (1986).

49. D. B. Rubin, Causal Inference Using Potential Outcomes. *J. Am. Stat. Assoc.* **100**, 322–331 (2005).

50. K. Kimmel, L. E. Dee, M. L. Avolio, P. J. Ferraro, Causal assumptions and causal inference in ecological experiments. *Trends Ecol. Evol.* **36**, 1141–1152 (2021).

51. S. Arif, M. A. MacNeil, Predictive models aren't for causal inference. *Ecol. Lett.* **25**, 1741–1745 (2022).

52. M. F. Bellemare, C. J. Wichman, Elasticities and the Inverse Hyperbolic Sine Transformation. *Oxf. Bull. Econ. Stat.* (2020), doi:10.1111/obes.12325.

53. B. J. Wilsey, H. W. Polley, Realistically low species evenness does not alter grassland species-richness-productivity relationships. *Ecology*. **85**, 2693–2700 (2004).

54. M. A. Leibold, J. M. Chase, S. K. M. Ernest, Community assembly and the functioning of ecosystems: how metacommunity processes alter ecosystems attributes. *Ecology*. **98**, 909–919 (2017).

55. Y. Wang, M. W. Cadotte, Y. Chen, L. H. Fraser, Y. Zhang, F. Huang, S. Luo, N. Shi, M. Loreau, Global evidence of positive biodiversity effects on spatial ecosystem stability in natural grasslands. *Nat. Commun.* **10**, 1–9 (2019).

56. J. B. Grace, T. M. Anderson, E. W. Seabloom, E. T. Borer, P. B. Adler, W. S. Harpole, Y. Hautier, H. Hillebrand, E. M. Lind, M. Pärtel, J. D. Bakker, Y. M. Buckley, M. J. Crawley, E. I. Damschen, K. F. Davies, P. A. Fay, J. Firn, D. S. Gruner, S. M. Prober, M. D. Smith, Integrative modelling reveals mechanisms linking productivity and plant species richness. *Nature*. **529**, 390–393 (2016).

57. A. J. MacDonald, A. E. Larsen, A. J. Plantinga, Missing the people for the trees: Identifying coupled natural–human system feedbacks driving the ecology of Lyme disease. *J. Appl. Ecol.* **56**, 354–364 (2019).

58. S. Creel, M. Creel, Density dependence and climate effects in Rocky Mountain elk: An application of regression with instrumental variables for population time series with sampling error. *J. Anim. Ecol.* **78**, 1291–1297 (2009).

59. G. W. Imbens, Instrumental Variables: An Econometrician's Perspective. *Stat. Sci.* **29**, 323–358 (2014).

60. P. J. Ferraro, J. N. Sanchirico, M. D. Smith, Causal inference in coupled human and natural systems. *Proc. Natl. Acad. Sci.*, 201805563 (2018).

61. E. T. Borer, E. W. Seabloom, D. S. Gruner, W. S. Harpole, H. Hillebrand, E. M. Lind, P. B. Adler, J. Alberti, T. M. Anderson, J. D. Bakker, L. Biederman, D. Blumenthal, C. S. Brown, L. A. Brudvig, Y. M. Buckley, M. Cadotte, C. Chu, E. E. Cleland, M. J. Crawley, P. Daleo, E. I. Damschen, K. F. Davies, N. M. Decrappeo, G. Du, J. Firn, Y. Hautier, R. W. Heckman, A. Hector, J. Hillerislambers, O. Iribarne, J. A. Klein, J. M. H. Knops, K. J. La Pierre, A. D. B. Leakey, W. Li, A. S. MacDougall, R. L. McCulley, B. A. Melbourne, C. E. Mitchell, J. L. Moore, B. Mortensen, L. R. O'Halloran, J. L. Orrock, J. Pascual, S. M. Prober, D. A. Pyke, A. C. Risch, M. Schuetz, M. D. Smith, C. J. Stevens, L. L. Sullivan, R. J. Williams, P. D. Wragg, J. P. Wright, L. H. Yang, Herbivores and nutrients control grassland plant diversity via light limitation. *Nature* (2014), doi:10.1038/nature13144.

62. E. W. Seabloom, P. B. Adler, J. Alberti, L. Biederman, Y. M. Buckley, M. W. Cadotte, S. L. Collins, L. Dee, P. A. Fay, J. Firn, N. Hagenah, W. S. Harpole, Y. Hautier, A. Hector, S. E. Hobbie, F. Isbell, J. M. H. Knops, K. J. Komatsu, R. Laungani, A. MacDougall, R. L. McCulley, J. L. Moore, J. W. Morgan, T. Ohlert, S. M. Prober, A. C. Risch, M. Schuetz, C. J. Stevens, E. T. Borer, Increasing effects of chronic nutrient enrichment on plant diversity loss and ecosystem productivity over time. *Ecology* (2021), doi:10.1002/ecy.3218.

63. J. L. M. Olea, C. Pflueger, A Robust Test for Weak Instruments. *J. Bus. Econ. Stat.* (2013), doi:10.1080/00401706.2013.806694.

64. P. J. Ferraro, J. N. Sanchirico, M. D. Smith, Causal inference in coupled human and natural systems. *Proc. Natl. Acad. Sci.*, 201805563 (2018).

65. N. Beck, J. N. Katz, Modeling dynamics in time-series-cross-section political economy data. *Annu. Rev. Polit. Sci.* **14**, 331–352 (2011).

66. J. G. Altonji, T. E. Elder, C. R. Taber, Selection on Observed and Unobserved Variables: Assessing the Effectiveness of Catholic Schools. *J. Polit. Econ.* **113**, 151–184 (2005).

67. E. Oster, Unobservable Selection and Coefficient Stability: Theory and Evidence. *J. Bus. Econ. Stat.* **0**, 1–18 (2017).

68. A. Bell, M. Fairbrother, K. Jones, Fixed and random effects models: making an informed choice. *Qual. Quant.* **53**, 1051–1074 (2019).

69. A. E. Larsen, K. Meng, B. E. Kendall, Causal Analysis in Control-Impact Ecological Studies with Observational Data. *Methods Ecol. Evol.*, 2041–210X.13190 (2019).

70. P. Allison, *Fixed Effects Regression Models* (2012).

71. B. J. Enquist, X. Feng, B. Boyle, B. Maitner, E. A. Newman, P. M. Jørgensen, P. R. Roehrdanz, B. M. Thiers, J. R. Burger, R. T. Corlett, T. L. P. Couvreur, G. Dauby, J. C. Donoghue, W. Foden, J. C. Lovett, P. A. Marquet, C. Merow, G. Midgley, N. Morueta-Holme, D. M. Neves, A. T. Oliveira-Filho, N. J. B. Kraft, D. S. Park, R. K. Peet, M. Pillet, J. M. Serra-Diaz, B. Sandel, M. Schildhauer, I. Šímová, C. Violle, J. J. Wieringa, S. K. Wiser, L. Hannah, J. C. Svenning, B. J. McGill, The commonness of rarity: Global and

future distribution of rarity across land plants. *Sci. Adv.* **5**, 1–14 (2019).

72. F. W. Preston, With respect to commonness or rarity. *Ecology*. **29**, 254–283 (1948).

73. D. F. Sax, S. D. Gaines, Species invasions and extinction: The future of native biodiversity on islands. *Light Evol.* **2**, 85–106 (2009).

74. S. S. Parker, W. S. Harpole, E. W. Seabloom, Plant species natural abundances are determined by their growth and modification of soil resources in monoculture. *Plant Soil*. **445**, 273–287 (2019).

75. M. D. Smith, A. K. Knapp, Dominant species maintain ecosystem function with non-random species loss. *Ecol. Lett.* **6**, 509–517 (2003).

76. M. L. Avolio, E. J. Forrestel, C. C. Chang, K. J. La Pierre, K. T. Burghardt, M. D. Smith, Demystifying dominant species. *New Phytol.* **223**, 1106–1126 (2019).

77. D. S. Srivastava, M. Vellend, Biodiversity-ecosystem function research: Is it relevant to conservation? *Annu. Rev. Ecol. Evol. Syst.* **36**, 267–294 (2005).

78. P. B. Reich, D. Tilman, J. Craine, D. Ellsworth, M. G. Tjoelker, J. Knops, D. Wedin, S. Naeem, D. Bahauddin, J. Goth, W. Bengtson, T. D. Lee, Do species and functional groups differ in acquisition and use of C, N and water under varying atmospheric CO2 and N availability regimes? A field test with 16 grassland species. *New Phytol.* **150**, 435–448 (2001).

79. A. Weigelt, E. Marquard, V. M. Temperton, C. Roscher, C. Scherber, P. N. Mwangi, S. Felten, N. Buchmann, B. Schmid, E.-D. Schulze, W. W. Weisser, The Jena Experiment: six years of data from a grassland biodiversity experiment. *Ecology*. **91**, 930–931 (2010).

80. B. J. Wilsey, T. B. Teaschner, P. P. Daneshgar, F. I. Isbell, H. W. Polley, Biodiversity maintenance mechanisms differ between native and novel exotic-dominated communities. *Ecol. Lett.* **12**, 432–442 (2009).

81. H. Ratcliffe, M. Ahlering, D. Carlson, S. Vacek, A. Allstadt, L. E. Dee, Invasive species do not exploit early growing seasons in burned tallgrass prairies. *Ecol. Appl.* (2022), doi:10.1002/eap.2641.

82. K. Kimmel, L. E. Dee, M. L. Avolio, P. J. Ferraro, Causal assumptions and causal inference in ecological experiments. *Trends Ecol. Evol.* (2021), , doi:10.1016/j.tree.2021.08.008.

83. B. J. Wilsey, H. Wayne Polley, Aboveground productivity and root-shoot allocation differ between native and introduced grass species. *Oecologia*. **150**, 300–309 (2006).

84. C. D'Antonio, S. E. Hobbie, in *Species invasions: insights into ecology, evolution and biogeography.*, G. S. Sax DF, Stachowicz JJ, Ed. (Sinauer Associates, Inc., Sunderland, MA, 2005), pp. 65–85.

85. M. Lechner, A. Strittmatter, Practical procedures to deal with common support problems in matching estimation. *Econom. Rev.* **38**, 193–207 (2019).

86. K. A. Bollen, J. Pearl, *Handbook of Causal Analysis for Social Research* (Springer Netherlands, Dordrecht, 2013; http://link.springer.com/10.1007/978-94-007-6094-3), *Handbooks of Sociology and Social Research*.

87. P. W. Holland, *ETS Res. Rep. Ser.*, in press, doi:10.1002/j.2330-8516.1988.tb00270.x.

88. P. J. Ferraro, J. J. Miranda, Panel Data Designs and Estimators as Substitutes for Randomized Controlled Trials in the Evaluation of Public Programs. *J. Assoc. Environ. Resour. Econ.* **4**, 281–317 (2017).

89. E. Battistin, A. Chesher, Treatment effect estimation with covariate measurement error. *J. Econom.* **178** (2014), doi:10.1016/j.jeconom.2013.10.010.

90. E. Allan, P. Manning, F. Alt, J. Binkenstein, S. Blaser, N. Blüthgen, S. Böhm, F. Grassein, N. Hölzel, V. H. Klaus, T. Kleinebecker, E. K. Morris, Y. Oelmann, D. Prati, S. C. Renner, M. C. Rillig, M. Schaefer, M. Schloter, B. Schmitt, I. Schöning, M. Schrumpf, E. Solly, E. Sorkau, J. Steckel, I. Steffen-Dewenter, B. Stempfhuber, M. Tschapka, C. N. Weiner, W. W. Weisser, M. Werner, C. Westphal, W. Wilcke, M. Fischer, Land use intensification alters ecosystem multifunctionality via loss of biodiversity and changes to functional composition. *Ecol. Lett.* **18**, 834–843 (2015).

91. P. J. Ferraro, A. Agrawal, Synthesizing Evidence in Sustainability Science through Harmonized Experiments: Community monitoring in common-pool resources. *Proc. Natl.*

Acad. Sci. USA (2021).

92. U. Brose, H. Hillebrand, Biodiversity and ecosystem functioning in dynamic landscapes. *Philos. Trans. R. Soc. B Biol. Sci.* **371** (2016).
