# Supplementary material for: Clarifying the effect of biodiversity on productivity in natural ecosystems with longitudinal data and methods for causal inference
Source: Nat Commun. 2023 May 5;14:2607. doi: 10.1038/s41467-023-37194-5 (PMC10163230; doi:10.1038/s41467-023-37194-5)
Supplement: Supplementary file 3 — Supplementary Dataset 1 [file 41467_2023_37194_MOESM3_ESM.zip › tutorial in R/RMarkdown_panel_designs_Dee_and_Severen.html]

Panel Data and Fixed Effects Designs for Ecology


# Panel Data and Fixed Effects Designs for Ecology

#### Online tutorial for the main analyses run in Dee et al. Nature Communications. This tutorial is made by Laura Dee and Chris Severen with data from the Nutrient Network

#### Last updated: 2023-02-16


# 1 Setup and Load Data

The data used in this tutorial comes from the Nutrient Network. It has been cleaned and processed and is ready to use. The data correspond to what we use in the paper for the main analysis, and consists of control plots from 43 NutNet sites. Each site includes one or more control plots that we use. We only use plots for which we have at least 5 years of data.

We’ll primarily use the *fixest* package for analysis; the other packages (*tidyverse*, *data.table*, *lme4*) aid data wrangling and comparing research designs.

```
library(tidyverse) #v 1.3.2
library(data.table) # v 1.14.6
library(fixest) # v 0.11
library(lme4)  # Need at least version 1.1-26
library(knitr) #v1.41
library(ggplot2) # v 3.4.0

cdir <- getwd()
comb <- fread(paste(cdir,"/cleaned_comb_data.csv",sep=""),na.strings='NA')
comb$V1 = NULL
comb$site <- comb$site_code
## Make dummy variables for panel regression ##
# make year a character, to be a dummy variable: 
 comb$year <- as.character(comb$year)
#make a factor that is site by year
comb[, site.by.yeardummy := paste(site_code, year, sep = "_")]
```

The *comb* dataset contains a wide number of variables; we’ll mainly be using the variables *live\_mass* and *rich*. Also important are the *site\_code*, *plot*, and *year* variables, which we use to create fixed effects (site-by-year fixed were created with `"r comb[, site.by.yeardummy := paste(site_code, year, sep = "_")]"`).

# 2 Standard Approaches

For causal inference, we need to answer, *how much does a change in \(X\) lead to a change in \(Y\)*. Causal inference involves ruling out **rival explanations** for an estimated relationship. This aim and requirement differs from other research tasks (e.g., predicting where total productivity will be the highest, which can be done without knowing the true underlying causes of productivity). Thus, the criteria for evaluating causal inference differ from other task. For example, prediction or forecasting of rely on model fit measures, such as R-squared values or mean squared error (see Ferraro, Sanchirico, and Smith 2018).

We evaluate causal inference by assessing the credibility and validity of the assumptions made by the research design for each dataset, system, and question (reviewed in Morgan and Winship 2014; Ferraro, Sanchirico, and Smith 2018), as we will illustrate next. Being explicit about these assumptions and evaluating how reasonable they are is important for causal inference.

Dr. Paul Allison has an excellent blog post explaining why R-squared values are NOT important the important criteria for causal interpretations compared to the credibility and validity of the assumptions made by the research design.

## 2.1 Simple correlations in single years

Let’s begin by looking at simple correlations. We will examine both the overall correlation and correlations within a single year (i.e., using cross-sectional variation). We proceed with a linear regression framework (2.1), using the log of productivity (measured as live aboveground biomass) as the outcome and the log species richness as the explanatory variable. We’ve found that a log-log specification captures the nature of the relationship in our data well (see Figure 2.1 below and the paper for details).

Figure 2.1: log(Live Mass) as a function of log(Richness).

We initially estimate and report \(\beta\) in:
\[\begin{equation}
\ln(\text{Live Mass}\_{pst}) = \alpha + \beta \ln(\text{Richness}\_{pst}) + e\_{pst}
\tag{2.1}
\end{equation}\]
where \(p\) indexes plots, \(s\) indexes sites, and \(t\) indexes years. The unobserved error term is \(e\_{pst}\). We include a constant \(\alpha\), but do not report estimates of it as it tells us little.

We report results below using all years of data first, and then report results using two individual years: 2012 and 2013. Here, as everywhere below, we **cluster standard errors** by plot to reflect serial correlation in errors terms within a plot across years (we do not assume that errors are iid). Note that when we use only a single year of data, this is equivalent to using heteroskedasticity-robust errors.

```
SimpleCorrAll <- feols(log(live_mass) ~ log(rich), comb, cluster = "newplotid") 

SimpleCorr2012 <- comb %>%
  filter(year==2012) %>%
  feols(log(live_mass) ~ log(rich), ., cluster = "newplotid") 

SimpleCorr2013 <- comb %>%
  filter(year==2013) %>%
  feols(log(live_mass) ~ log(rich), ., cluster = "newplotid") 

etable(SimpleCorrAll, SimpleCorr2012, SimpleCorr2013, 
          cluster = "newplotid", 
          drop = "Intercept", 
          subtitles = c("Data All Years", "Data in 2012", "Data in 2013"))
```

|  | SimpleCorrAll | SimpleCorr2012 | SimpleCorr2013 |
| --- | --- | --- | --- |
|  | Data All Years | Data in 2012 | Data in 2013 |
| Dependent Var.: | log(live\_mass) | log(live\_mass) | log(live\_mass) |
|  |  |  |  |
| Constant | 5.288\*\*\* (0.2398) | 4.915\*\*\* (0.4111) | 6.421\*\*\* (0.2294) |
| log(rich) | 0.0699 (0.0952) | 0.1849 (0.1706) | -0.3766\*\*\* (0.1006) |
| \_\_\_\_\_\_\_\_\_\_\_\_\_\_\_ | \_\_\_\_\_\_\_\_\_\_\_\_\_\_\_\_\_ | \_\_\_\_\_\_\_\_\_\_\_\_\_\_\_\_\_ | \_\_\_\_\_\_\_\_\_\_\_\_\_\_\_\_\_\_\_ |
| S.E.: Clustered | by: newplotid | by: newplotid | by: newplotid |
| Observations | 1,231 | 145 | 121 |
| R2 | 0.00152 | 0.00928 | 0.08045 |
| Adj. R2 | 0.00071 | 0.00235 | 0.07272 |

As you can see, using all years of data gives a non-significant positive relationship between productivity and richness. In just the 2012 data, the coefficient is larger in magnitude, but still not significant. Finally, just using 2013 data, the coefficient switches signs and becomes significant.

So… which one to believe? Well, probably none of these, because they likely do not identify the true causal effect of richness on productivity. Their variability highlights that these estimates, which rely on non-experimental **cross-sectional** data, are likely contaminated by omitted variable bias.

When does \(\hat{\beta}\) capture a causal relationship? When there are no unobservables that are correlated with richness that also influence productivity: \(\mathbb{E}[e\_{pst} \times \ln(\text{Richness}\_{pst})]=0\) (i.e., \(e\_{pst}\) and \(\ln(\text{Richness}\_{pst})\) aren’t correlated). In the above results, there’s probably stuff in \(e\) that is correlated with richness, like precipitation, disturbance, land-use history, soil characteristics, and other characteristics of sites and plots.

A **Directed Acyclic Diagram** (DAG) can help us see the challenges of omitted, and potentially confounding, variables more clearly. In the above analyses from equation (2.1), \(\beta\) is only identified if we assume that any variables that matter but that we omitted are uncorrelated with richness.
One benefit of a DAG is that it makes transparent the assumptions on which one relies for making causal claims from observable data. A DAG therefore allows the researcher and the reader to better judge the credibility of the causal claims from a specific research design. Another way to view this benefit is that a causal graph helps identify the sources of variation in a causal variable and in its outcome, thereby emphasizing potential sources of bias that must be addressed in a research design and pointing to designs that can address these sources of **statistical bias** (Morgan and Winship 2014).

In our case, many variables that are correlated with biodiversity can also drive productivity (Fig. 1B – main text), creating the possibility for rival explanations and biased estimates for estimated effects from observations. For example, climatic conditions, soil nutrients, evolutionary history, and historic contingency during community assembly are all related to both productivity and biodiversity (Loreau 1998; Fukami and Nakajima 2011; Grace et al. 2016). With a common driver of both variables that is not included in a model, two variables (i.e., biodiversity and an ecosystem function) may be correlated, even when there is no causal relationship between them. Similarly, no correlation between variables does not imply a lack of causation. Causal relationships can also be masked when examining correlations, due to an omitted variable (e.g., nitrogen addition), which positively affects productivity but negatively affects richness (Isbell et al. 2013). Models that do not control for that common driver will consequently tend to give estimates that do not correspond to causal effects of biodiversity on productivity (or *vice versa*).

  

  

Figure 1B (main text – right panel) is known as a directed acyclic causal graph (DAG) and is a visualization of qualitative causal assumptions ((Pearl 2009, 2011; Fieberg and Ditmer 2012; Schoolmaster Jr et al. 2013; Schoolmaster Jr, Zirbel, and Cronin 2020; Grace and Irvine 2020)). A DAG encodes knowledge and beliefs about how a system works. The graphical relations depicted in the DAG encode causal claims – not just representations of associations. A directed edge (e.g., R –> P) depicts a claim about the results of many hypothetical experiments, whereby if every other variable represented in the graph is held fixed, R and P will covary if R if manipulated, but not if P is manipulated (note, a DAG assumes that one can isolate the effect of R on P, but does not imply that P can never affect R; another DAG may represent the reverse direction, P –> R).

TAKEAWAY:

Omitted variable bias is a pervasive feature in observational analysis, and the assumptions that permit identification of causal effects are unlikely to hold when using cross-sectional variation.

## 2.2 Common Ecological Design - Multivariate Regression

Of course, in the above correlations, we include plots in sites from across the world, implicitly comparing grasslands in warmer climates with those in cooler ones, or wetter with dryer, or Europe with the Americas. There are a lot of differences between these places!

A common response to this problem is to try to measure these differences and include them in the model. In the causal inference literature, this is known as **“conditioning on observables”** or Pearl’s **back-door criteria**. Conditioning on observables is convenient but makes strong assumptions for causal inference, namely the **“Selection on Observables” Assumption.** Informally, this assumption implies that confounding variables that could introduce bias into a design are known and observable to the researcher. The bias they introduce into an estimator can be eliminated (controlled, blocked) by conditioning strategies, such as regression, matching, or stratification methods. To read more, see (Morgan and Winship 2014). We can visualize this assumption by modifying our DAG, and using some examples in R:

  

  

To explore the consequences of adding in covariates, we show the results of five models below. The first column repeats the first column from above. The second column adds in soil chemistry covariates, the third column instead adds weather covariates, and the fourth instead adds management variables plus habitat. The last columns adds in everything. For the purposes of this tutorial, we only show coefficient estimates for richness in the following table, even though the other terms are included in the model.

```
SoilCovars <- feols(log(live_mass) ~ log(rich) +
                      pct_C + pct_N + ppm_P + ppm_K + ppm_Na + ppm_Mg + ppm_S + ppm_Na + ppm_Zn +  ppm_Mn +  ppm_Fe + ppm_Cu + ppm_B +
                      pH + PercentSand + PercentSilt + PercentClay, 
                    comb, cluster = "newplotid") 

WeatherCovars <- feols(log(live_mass) ~ log(rich) +
                         elevation + TEMP_VAR_v2 + MIN_TEMP_v2 + MAX_TEMP_v2 + TEMP_WET_Q_v2 + TEMP_DRY_Q_v2 + TEMP_WARM_Q_v2 + 
                         TEMP_COLD_Q_v2, 
                       comb, cluster = "newplotid") 

MgmtCovars <- feols(log(live_mass) ~ log(rich) +
                      as.factor(habitat) + managed + burned + grazed + anthropogenic, 
                    comb, cluster = "newplotid") 

AllCovars <- feols(log(live_mass) ~ log(rich) +
                     pct_C + pct_N + ppm_P + ppm_K + ppm_Na + ppm_Mg + ppm_S + ppm_Na + ppm_Zn +  ppm_Mn +  ppm_Fe + ppm_Cu + ppm_B +
                     pH + PercentSand + PercentSilt + PercentClay +
                     elevation + TEMP_VAR_v2 + MIN_TEMP_v2 + MAX_TEMP_v2 + TEMP_WET_Q_v2 + TEMP_DRY_Q_v2 + TEMP_WARM_Q_v2 +
                     TEMP_COLD_Q_v2 + as.factor(habitat) + managed + burned + grazed + anthropogenic, 
                   comb, cluster = "newplotid") 

etable(SimpleCorrAll, SoilCovars, WeatherCovars, MgmtCovars, AllCovars,
          cluster = "newplotid", 
          drop = "!rich", 
          subtitles = c("Data All Years", "+ Soil", "+ Weather", "+ Management", "+ All"))
```

|  | SimpleCorrAll | SoilCovars | WeatherCovars | MgmtCovars | AllCovars |
| --- | --- | --- | --- | --- | --- |
|  | Data All Years | + Soil | + Weather | + Management | + All |
| Dependent Var.: | log(live\_mass) | log(live\_mass) | log(live\_mass) | log(live\_mass) | log(live\_mass) |
|  |  |  |  |  |  |
| log(rich) | 0.0699 (0.0952) | 0.2251\* (0.1127) | 0.0203 (0.0907) | 0.0275 (0.0822) | 0.4190\* (0.1649) |
| \_\_\_\_\_\_\_\_\_\_\_\_\_\_\_ | \_\_\_\_\_\_\_\_\_\_\_\_\_\_\_ | \_\_\_\_\_\_\_\_\_\_\_\_\_\_\_\_ | \_\_\_\_\_\_\_\_\_\_\_\_\_\_\_ | \_\_\_\_\_\_\_\_\_\_\_\_\_\_\_ | \_\_\_\_\_\_\_\_\_\_\_\_\_\_\_\_ |
| S.E.: Clustered | by: newplotid | by: newplotid | by: newplotid | by: newplotid | by: newplotid |
| Observations | 1,231 | 675 | 1,231 | 1,231 | 675 |
| R2 | 0.00152 | 0.33781 | 0.20626 | 0.37496 | 0.51377 |
| Adj. R2 | 0.00071 | 0.32068 | 0.20041 | 0.36568 | 0.48309 |

Estimates jump around depending on which covariates are used! This is likely a sign of some sort of omitted variables bias. Even though we consecutively explain more and more of the variation in the data, we are not necessarily any closer to a causal relationship.

There are a few other points to make about the above results that speak the practice of science.

1.First, the number of observations changes based on which controls we use. Columns 2 and 5, where we only have 675 observations, might be on highly selected and not representative of the overall populations under study. A careful analysis could do any of a number of approaches to control for that, but far too often we just say ``This is what I have (all the) data for, so this what I estimate.’’ It would be nice if there were a way to move forward even if we were unable to collect data on all the variables we wanted.

2.Second, and more perniciously, is specification hunting. What’s to keep from only displaying results in columns 2 and 5 above, with no mention of the other results. P-hacking is something we should all be concerned about, but it’s really easy to twiddle and play until one gets just the right set of results that agree with ones hypothesis.

3.Lastly, we assumed that all the covariates entered linearly and did not interact. What if what really matters is the interaction of nitrogen and precipitation? How do we capture that? We could include interactions and quadratics, or splines, but at some point, the number of covariates will exceed our sample size and nothing is identified.

TAKEAWAY:

It is hard to assume the we observe and correctly control for all confounding variables when analyzing cross-sectional data

# 3 Fixed Effects: Changing the Source of Variation

We now move on to the main course: Let’s switch up where the **identification** comes from.

## 3.1 Plot Fixed Effects

Let’s ignore sites for a minute, and just think about the plots that lie in a single site. We’re going to estimate the following model:
\[\begin{equation}
\ln(\text{Live Mass}\_{pt}) = \beta \ln(\text{Richness}\_{pt}) + \delta\_p + \mu\_t + e\_{pt}
\end{equation}\]
where we’ve added the term \(\delta\_p\). This represents a vector of plot-specific fixed effect—a dummy variable for each plot. We also add time fixed effects (\(\mu\_t\), a dummy for each year) to control for the common differences to all plots in a year (in a site). We’ll touch on that more later, but really, the *plot fixed effects are of greatest consequence*.

What does adding this vector of plot dummy variables do? Two big things. First, it controls for any and all time-invariant features of the plot, whether we observe them **or not**!!! To see this, imagine putting in a variable \(x\_p\) into the above equation linearly with the coefficient \(\gamma\). We wouldn’t actually be able to estimate \(\gamma x\_p\); it’s already a component of \(\delta\_p\). Don’t know what functional for you should use for \(x\_p\) or whether it should be interacted with another variable? That’s fine, that’s already included in \(\delta\_p\)! We get a whole lot for the inclusion of this variable. Let’s see our updated DAG: now we have removed confounding effects from plot-level attributes whether we can measure them or not!
  

  

Second, and most importantly conceptually, is that we are no longer directly comparing different plots; we aren’t using cross-sectional variation any more. Instead, we are using variation in richness and productivity within the same plot *over time*. So, we’re implicitly comparing a plot in year \(t\) with this same plot in year \(t+k\) for some \(k\). Another way to see this is that we could write a very similar equation in differences (ignore the \(\mu\_t\) for a moment):
\[\begin{equation}
\left(\ln(\text{Live Mass}\_{pt})-\ln(\text{Live Mass}\_{pt-1}) \right)= \beta \left( \ln(\text{Richness}\_{pt}) - \ln(\text{Richness}\_{pt-1}) \right) + \left( e\_{pt} - e\_{pt-1}\right)
\end{equation}\]
Where did \(\lambda\_p\) go? Well, \(\lambda\_p-\lambda\_p=0\), so we don’t need it. (NB: We could also subtract the mean of each variable over time within each plot and arrive at a similar estimator. There are subtle differences between the two approaches that depend on the nature of the error terms \(e\), but they draw on the same source of variation).

What do we have to assume for a causal interpretation? There are a couple of different assumptions we could choose; I think it’s easiest to frame it like this: \(\mathbb{E}[ (e\_{pt} - e\_{pt-1}) \times (\ln(\text{Richness}\_{pt}) - \ln(\text{Richness}\_{pt-1}))]=0\). That is, changes in richness are uncorrelated with *changes in* unobserved determinants of richness. Because time-invariant unobservable variables do not change, they are no longer a concern! Instead, we’re concerned if movements in some unobserved factor could both be driving our outcome variable and be correlated with richness.

What’s the cost? Well, there are a few to consider, but some really aren’t much of a restriction:

1. We use to worry about computational issues. Instead of differencing, we could have including the additional fixed effects as regressors. Were this a large vector, computation could have become difficult. This is rarely a concern thanks to better computers and better techniques.
2. We need longitudinal (panel) data (or repeated cross sections in some special circumstances). This is why economists get so much use out of administrative data! This data is rarer in ecology, but with the growth in LTERs and other multi-year sites, this will be less of a constraint.
3. Our target of interest has to be time-varying. Any interesting time-invariant factors have been removed from the equation (literally).

Figures 3.1 and 3.2 illustrate graphically what the plot fixed effects do to the outcome variable (productivity). Figures 3.1 is just the raw data, and shows log(live mass) in four plots split between two sites (at the Sedgwick Reserve [sedg.us] and at the Sevilleta LTER [sevi.us]). Sedgwick has higher productivity on average. The productivity at these sites also appear to be following different trajectories through time (e.g., note the dip in productivity at Sevilleta in 2009). The plot fixed effects remove the average productivity in each site, as shown in Figure 3.2. They do not remove site-and-year specific sources of confounding variation (e.g., if a more extreme drought happened at Sevilleta than at Sedgwick in 2009 affecting both productivity and richness); we turn to eliminating site and year specific confounding variables below in 3.3.

Figure 3.1: Raw variation in live mass at four plots across two sites.

Figure 3.2: Variation demaned by plot in live mass at four plots across two sites.

To the statistical model: We’re first going to estimate the following equation site-by-site on the five sites with the largest number of observations (in terms of the number plot-years we observe; see Table S1).
\[\begin{equation}
\ln(\text{Live Mass}\_{pt}) = \beta \ln(\text{Richness}\_{pt}) + \delta\_p + \mu\_t + e\_{pt}
\end{equation}\]
The year fixed effects \(\mu\_t\) control for *time-varying* factors (observed or unobserved) that affect all plots at the site under consideration. For example, suppose 2007 was a particularly damp and rainy year at the site; \(\mu\_t\) controls for the average impact of that across all plots.
Because what happens at one site in a year is probably very different from what happens at a different site in the same year, we estimate these separately for each site. This will make the point estimates for each site less precise (especially because we’re clustering by plot), but this is just for illustration’s sake.

```
PlotFE_1 <- comb %>%
  filter(site_code=="cdcr.us") %>%
  feols(log(live_mass) ~ log(rich) | newplotid + year, ., cluster = "newplotid")

PlotFE_2 <- comb %>%
  filter(site_code=="cdpt.us") %>%
  feols(log(live_mass) ~ log(rich) | newplotid + year, ., cluster = "newplotid")

PlotFE_3 <- comb %>%
  filter(site_code=="koffler.ca") %>%
  feols(log(live_mass) ~ log(rich) | newplotid + year, ., cluster = "newplotid")

PlotFE_4 <- comb %>%
  filter(site_code=="sedg.us") %>%
  feols(log(live_mass) ~ log(rich) | newplotid + year, ., cluster = "newplotid")

PlotFE_5 <- comb %>%
  filter(site_code=="sier.us") %>%
  feols(log(live_mass) ~ log(rich) | newplotid + year, ., cluster = "newplotid")

etable(SimpleCorrAll, PlotFE_1, PlotFE_2, PlotFE_3, PlotFE_4, PlotFE_5,
          cluster = "newplotid", 
          drop = "!rich", 
          subtitles = c("Data All Years","US - CDCR", "US - CDPT", "CA - Koffler", "US - SEDG", "US - SIER" ))
```

|  | SimpleCorrAll | PlotFE\_1 | PlotFE\_2 | PlotFE\_3 | PlotFE\_4 | PlotFE\_5 |
| --- | --- | --- | --- | --- | --- | --- |
|  | Data All Years | US - CDCR | US - CDPT | CA - Koffler | US - SEDG | US - SIER |
| Dependent Var.: | log(live\_mass) | log(live\_mass) | log(live\_mass) | log(live\_mass) | log(live\_mass) | log(live\_mass) |
|  |  |  |  |  |  |  |
| log(rich) | 0.0699 (0.0952) | -0.0972 (0.2189) | -0.6247. (0.2475) | 0.0257 (0.2501) | -0.0569 (0.1133) | -0.2651 (0.2472) |
| Fixed-Effects: | ————— | —————- | —————– | ————— | —————- | —————- |
| newplotid | No | Yes | Yes | Yes | Yes | Yes |
| year | No | Yes | Yes | Yes | Yes | Yes |
| \_\_\_\_\_\_\_\_\_\_\_\_\_\_\_ | \_\_\_\_\_\_\_\_\_\_\_\_\_\_\_ | \_\_\_\_\_\_\_\_\_\_\_\_\_\_\_\_ | \_\_\_\_\_\_\_\_\_\_\_\_\_\_\_\_\_ | \_\_\_\_\_\_\_\_\_\_\_\_\_\_\_ | \_\_\_\_\_\_\_\_\_\_\_\_\_\_\_\_ | \_\_\_\_\_\_\_\_\_\_\_\_\_\_\_\_ |
| S.E.: Clustered | by: newplotid | by: newplotid | by: newplotid | by: newplotid | by: newplotid | by: newplotid |
| Observations | 1,231 | 55 | 60 | 72 | 66 | 53 |
| R2 | 0.00152 | 0.74115 | 0.72916 | 0.26473 | 0.73402 | 0.62055 |
| Within R2 | – | 0.00325 | 0.05917 | 0.00011 | 0.00444 | 0.04260 |

We again show the bivariate correlation on all sites first (SimpleCorrAll), and then the estimate for each site. **Whoa!** Now we’re getting some negative coefficients (though mostly insignificant due to smaller effective sample sizes). We’re controlling for lots and lots of things that we couldn’t control for before, either because we didn’t think to include them or we couldn’t collect data on them. The R-squared values confirm that this is the case; we’re generally explaining much more of the data than before (but note: R-squared values are NOT important for causal interpretations generally). *Note that, with the plot fixed effects, we do not have much statistical power estimating sites individually.*

TAKEAWAY:

Using unit fixed effects in panel data shifts the identifying variation from across units to within units over time.

## 3.2 Bringing it all Together with Site-by-Year Fixed Effects

We now combine all sites together to give us more statistical power to detect effects. We do want to account for the fact that different sites experience different conditions in different years.
To do so in a flexible way, we include site-by-year fixed effects, \(\mu\_{st}\).
\[\begin{equation}
\ln(\text{Live Mass}\_{pst}) = \beta \ln(\text{Richness}\_{pst}) + \delta\_p + \delta\_{st} + e\_{pst}
\end{equation}\]
These additional fixed effects control for all time-varying effects that impact the site as whole (i.e., that apply to all the plots equally). Thus, they capture the first order effects of weather, among other factors that could shift outcomes for the site as whole. This gives us sufficient power to conduct conservative inference on our estimated average treatment effect.

To get a sense for what these site-by-year effects do, first recall Figure 3.2. Plots that are in the same site seem to have similar movements in productivity over time, even after controlling for plot fixed effects. The site-by-year fixed effects remove the average of everything that happens across the site in the data in a year (e.g., a drought at a site). Figure 3.3 removes this variation; see how the big drop in Sevilleta live mass in 2009 is much less Figure in 3.3.

Figure 3.3: Variation demaned by plot and site-by-year effects in live mass at four plots across two sites.

To provide confidence that the results are robust, we will also include a couple of time-varying controls, evenness and lagged richness. NB: To make sure we don’t drop locations with values of zero evenness, we use the inverse hyperbolic sine instead of the natural log. Note that we don’t need to worry about that for productivity or richness because they never take a zero value.

```
ihs <- function(x) {
  y <- log(x+sqrt(x^2 + 1))
}


MainMod_Rich     <- feols(log(live_mass) ~ log(rich)  | newplotid + site.by.yeardummy, comb) 
MainMod_RichEven <- feols(log(live_mass) ~ log(rich) + ihs(even) | newplotid + site.by.yeardummy, comb) 
MainMod_RichLag  <- feols(log(live_mass) ~ log(rich) + log(laggedrich) | newplotid + site.by.yeardummy, comb) 
MainMod_RichEvenLag <- feols(log(live_mass) ~ log(rich) + log(laggedrich) + ihs(even) | newplotid + site.by.yeardummy, comb)

etable(MainMod_Rich, MainMod_RichEven, MainMod_RichLag, MainMod_RichEvenLag,
          cluster = "newplotid")
```

|  | MainMod\_Rich | MainMod\_RichEven | MainMod\_RichLag | MainMod\_RichEve.. |
| --- | --- | --- | --- | --- |
| Dependent Var.: | log(live\_mass) | log(live\_mass) | log(live\_mass) | log(live\_mass) |
|  |  |  |  |  |
| log(rich) | -0.2418\*\* (0.0854) | -0.2237\*\* (0.0851) | -0.2185\* (0.0939) | -0.2057\* (0.0948) |
| ihs(even) |  | -0.1864 (0.2122) |  | -0.1450 (0.2387) |
| log(laggedrich) |  |  | -0.0146 (0.0905) | -0.0096 (0.0903) |
| Fixed-Effects: | —————— | —————— | —————– | —————– |
| newplotid | Yes | Yes | Yes | Yes |
| site.by.yeardummy | Yes | Yes | Yes | Yes |
| \_\_\_\_\_\_\_\_\_\_\_\_\_\_\_\_\_ | \_\_\_\_\_\_\_\_\_\_\_\_\_\_\_\_\_\_ | \_\_\_\_\_\_\_\_\_\_\_\_\_\_\_\_\_\_ | \_\_\_\_\_\_\_\_\_\_\_\_\_\_\_\_\_ | \_\_\_\_\_\_\_\_\_\_\_\_\_\_\_\_\_ |
| S.E.: Clustered | by: newplotid | by: newplotid | by: newplotid | by: newplotid |
| Observations | 1,231 | 1,231 | 1,093 | 1,093 |
| R2 | 0.86669 | 0.86689 | 0.87092 | 0.87103 |
| Within R2 | 0.01281 | 0.01425 | 0.01064 | 0.01148 |

As you can see, estimate on log richness are relatively stable across different specifications. Of special note: the coefficient on lagged richness (richness from the year before) is small and insignificant, given us confidence that our results reflect *contemporaneous* movement in richness, and not some factor that is also correlated with last year’s richness.

TAKEAWAY:

Fixed effects can alter the research design and control for a wide variety of potentially confounding factors!

Our updated DAG, including both plot and site-by-year fixed effects, show that we can control more flexibly for a broader set of confounding variables in space and time – critically, whether they are measured or not!

# 4 Graphical Depictions of Fixed Effects

Using simple plots of the relationships between richness and productivity (as log of richness and log of live mass), we can see the relationships shift from positive to negative as we add in fixed effects.

Figure 4.1 just shows the raw bivariate correlation, and is somewhat positive. Notice the range of variation across productivity and richness.

Figure 4.1: The bivariate relationship with no controls.

Figure 4.2 shows the relationship conditional on plot fixed effects. Note that this collapses the range of variation substantially. Recall that the plot fixed effects remove factors that are time-invariant and plot-specific. However, this graph still reflects the variation in sites across years.

Figure 4.2: Controlling for only plot-level attributes that do not change through time

Figure 4.3 continues to control for plot fixed effects, but also removes site-by-year variation via site-by-year fixed effects. This shifts the variation negative as factors that are common to sites within years no longer confound the relationship.

Figure 4.3: Controlling for plot attributes and attributes of sites that change through time

# 5 Random Effects Don’t Usually Cut It…

Researchers sometimes turn to random effects models to attempt to deal with confounding variables or *unobserved heterogeneity*. While random effects models have a number of useful properties (reviewed in Bell, Fairbrother, and Jones (2019)), they **do not solve endogeneity problems** on their own (see (Wooldridge 2010, 2015; Larsen, Meng, and Kendall 2019)). Any variable that is omitted from a random effects model is implicitly assumed to either have no effect on productivity or to be uncorrelated with explanatory variables. So, random effects on their own don’t really buy us anything more than conditioning on observables in terms of establishing causality.

To see this, consider again our simple model:
\[\begin{equation}
\ln(\text{Live Mass}\_{pt}) = \beta \ln(\text{Richness}\_{pt}) + \delta\_p + \mu\_t + e\_{pt}
\end{equation}\]
The random effects assumption is that \(\ln(\text{Richness}\_{pt})\) and \(\delta\_p\) are uncorrelated. This is a strong assumption; it says that no tunobservables (even time-invariant ones) are correlated with richness! So, random effects with no controls assume average rainfall or latitude don’t matter for richness. Of course, one could add controls, but then we’re back to the question of do we include the right measures of the controls in the right functional form, etc.

By contrast, recall that the fixed effects assumption allows for arbitrary correlation between richness and any time-invariant variables, whether observed or not. So, should you include a fifth-degree polynomial in latitude. Nope, just include the fixed effect.

Check out how the results below change (or not) in response to fixed effects (references for more details on the mathematics behind this are listed later on). First, consider the two linear models (without random effects) with different sets of soil variables:

```
## Linear Model with Soil Covariates
SoilCovars <- feols(log(live_mass) ~ log(rich) +
                      pct_C + pct_N + ppm_P + ppm_K + ppm_Na + ppm_Mg + ppm_S + ppm_Na + ppm_Zn +  ppm_Mn +  ppm_Fe + ppm_Cu + ppm_B, 
                    comb, cluster = "newplotid") 

## Linear Model with More Soil Covariates
SoilCovars_More <- feols(log(live_mass) ~ log(rich) +
                      pct_C + pct_N + ppm_P + ppm_K + ppm_Na + ppm_Mg + ppm_S + ppm_Na + ppm_Zn +  ppm_Mn +  ppm_Fe + ppm_Cu + ppm_B +
                      pH + PercentSand + PercentSilt + PercentClay, 
                    comb, cluster = "newplotid") 

etable(SoilCovars, SoilCovars_More,
          cluster = "newplotid", 
          drop = "!rich", 
          subtitles = c("Some soil covariates","More soil covariates"))
```

|  | SoilCovars | SoilCovars\_More |
| --- | --- | --- |
|  | Some soil covariates | More soil covariates |
| Dependent Var.: | log(live\_mass) | log(live\_mass) |
|  |  |  |
| log(rich) | 0.0309 (0.1213) | 0.2251\* (0.1127) |
| \_\_\_\_\_\_\_\_\_\_\_\_\_\_\_ | \_\_\_\_\_\_\_\_\_\_\_\_\_\_\_ | \_\_\_\_\_\_\_\_\_\_\_\_\_\_\_\_ |
| S.E.: Clustered | by: newplotid | by: newplotid |
| Observations | 959 | 675 |
| R2 | 0.19321 | 0.33781 |
| Adj. R2 | 0.18211 | 0.32068 |

See how the covariate of interest jumps around. Again, that’s a reminder that there are likely omitted variables that are pretty correlated with our variable of interest.

So, that’s our baseline. Now let’s see what happens with random effects!

```
## First, characteristics of soil influence both productivity and richness. We use covariates that characterize several aspects of the soil fertility and nutrients. We include plot and site random effects.
MixedMod_Rich_soil <- lmer(log(live_mass) ~ log(rich) + 
                             pct_C + pct_N + ppm_P + ppm_K + ppm_Na + ppm_Mg + ppm_S + ppm_Na + ppm_Zn +  ppm_Mn +  ppm_Fe + ppm_Cu + ppm_B +   pH + PercentSand + PercentSilt + PercentClay +    (1|newplotid) +   (1|site_code), comb, REML = F)
summary(MixedMod_Rich_soil)
```

```
## Linear mixed model fit by maximum likelihood  ['lmerMod']
## Formula: log(live_mass) ~ log(rich) + pct_C + pct_N + ppm_P + ppm_K +  
##     ppm_Na + ppm_Mg + ppm_S + ppm_Na + ppm_Zn + ppm_Mn + ppm_Fe +  
##     ppm_Cu + ppm_B + pH + PercentSand + PercentSilt + PercentClay +  
##     (1 | newplotid) + (1 | site_code)
##    Data: comb
## 
##      AIC      BIC   logLik deviance df.resid 
##   1644.8   1739.6   -801.4   1602.8      654 
## 
## Scaled residuals: 
##      Min       1Q   Median       3Q      Max 
## -12.0550  -0.4639   0.0515   0.5742   3.0163 
## 
## Random effects:
##  Groups    Name        Variance Std.Dev.
##  newplotid (Intercept) 0.0000   0.0000  
##  site_code (Intercept) 0.3677   0.6064  
##  Residual              0.5618   0.7495  
## Number of obs: 675, groups:  newplotid, 82; site_code, 28
## 
## Fixed effects:
##               Estimate Std. Error t value
## (Intercept) -1.716e+02  1.502e+02  -1.142
## log(rich)    3.647e-01  8.920e-02   4.089
## pct_C       -6.353e-02  7.675e-02  -0.828
## pct_N        9.655e-01  1.203e+00   0.803
## ppm_P        3.866e-03  2.703e-03   1.430
## ppm_K        2.754e-04  4.423e-04   0.623
## ppm_Na       7.475e-05  7.108e-04   0.105
## ppm_Mg      -4.267e-04  3.364e-04  -1.268
## ppm_S        1.336e-02  8.144e-03   1.641
## ppm_Zn       3.198e-03  2.356e-02   0.136
## ppm_Mn       2.119e-03  1.776e-03   1.193
## ppm_Fe      -1.670e-03  1.167e-03  -1.431
## ppm_Cu       8.377e-02  3.635e-02   2.304
## ppm_B       -1.110e-02  1.417e-01  -0.078
## pH          -3.519e-01  1.312e-01  -2.683
## PercentSand  1.776e+00  1.503e+00   1.182
## PercentSilt  1.785e+00  1.505e+00   1.187
## PercentClay  1.769e+00  1.502e+00   1.178
## fit warnings:
## Some predictor variables are on very different scales: consider rescaling
## optimizer (nloptwrap) convergence code: 0 (OK)
## boundary (singular) fit: see help('isSingular')
```

```
## We now bring in management variables, and retain the plot and site random effects.
MixedMod_Rich2 <- lmer(log(live_mass) ~ log(rich) + 
                         managed + burned + grazed + anthropogenic + 
                         pct_C + pct_N + ppm_P + ppm_K + ppm_Na + ppm_Mg + ppm_S + ppm_Na + ppm_Zn +  ppm_Mn +  ppm_Fe + ppm_Cu + ppm_B +   pH + PercentSand + PercentSilt + PercentClay +    (1|newplotid) +                             (1|site_code), comb, REML = F)
summary(MixedMod_Rich2 )
```

```
## Linear mixed model fit by maximum likelihood  ['lmerMod']
## Formula: 
## log(live_mass) ~ log(rich) + managed + burned + grazed + anthropogenic +  
##     pct_C + pct_N + ppm_P + ppm_K + ppm_Na + ppm_Mg + ppm_S +  
##     ppm_Na + ppm_Zn + ppm_Mn + ppm_Fe + ppm_Cu + ppm_B + pH +  
##     PercentSand + PercentSilt + PercentClay + (1 | newplotid) +  
##     (1 | site_code)
##    Data: comb
## 
##      AIC      BIC   logLik deviance df.resid 
##   1647.0   1759.9   -798.5   1597.0      650 
## 
## Scaled residuals: 
##      Min       1Q   Median       3Q      Max 
## -12.0999  -0.4682   0.0338   0.5783   2.8867 
## 
## Random effects:
##  Groups    Name        Variance Std.Dev.
##  newplotid (Intercept) 0.0000   0.000   
##  site_code (Intercept) 0.2323   0.482   
##  Residual              0.5670   0.753   
## Number of obs: 675, groups:  newplotid, 82; site_code, 28
## 
## Fixed effects:
##                 Estimate Std. Error t value
## (Intercept)   -2.021e+02  1.487e+02  -1.359
## log(rich)      3.813e-01  8.888e-02   4.290
## managed        4.002e-02  4.363e-01   0.092
## burned         3.517e-01  6.414e-01   0.548
## grazed        -9.319e-01  5.341e-01  -1.745
## anthropogenic  5.307e-01  4.124e-01   1.287
## pct_C         -3.663e-02  7.044e-02  -0.520
## pct_N          5.564e-01  1.093e+00   0.509
## ppm_P          3.648e-03  2.744e-03   1.330
## ppm_K          1.992e-04  4.272e-04   0.466
## ppm_Na        -1.607e-04  6.957e-04  -0.231
## ppm_Mg        -3.082e-04  3.052e-04  -1.010
## ppm_S          1.880e-02  7.666e-03   2.452
## ppm_Zn        -1.482e-02  2.301e-02  -0.644
## ppm_Mn         2.501e-03  1.736e-03   1.441
## ppm_Fe        -5.372e-04  1.170e-03  -0.459
## ppm_Cu         7.621e-02  3.546e-02   2.149
## ppm_B          3.652e-02  1.362e-01   0.268
## pH            -3.219e-01  1.269e-01  -2.536
## PercentSand    2.075e+00  1.488e+00   1.394
## PercentSilt    2.091e+00  1.490e+00   1.404
## PercentClay    2.067e+00  1.488e+00   1.389
## fit warnings:
## Some predictor variables are on very different scales: consider rescaling
## optimizer (nloptwrap) convergence code: 0 (OK)
## boundary (singular) fit: see help('isSingular')
```

```
## we also can bring in controls for country, habitat, and year, again retaining the plot and site random effects. 
MixedMod_Rich <- lmer(log(live_mass) ~ log(rich) + as.factor(country) + as.factor(habitat) + as.factor(year) + 
                        elevation + managed + burned + grazed + anthropogenic + 
                        TEMP_VAR_v2 + MIN_TEMP_v2 + MAX_TEMP_v2 + TEMP_WET_Q_v2 + TEMP_DRY_Q_v2 + TEMP_WARM_Q_v2 +                            TEMP_COLD_Q_v2 +   pct_C + pct_N + ppm_P + ppm_K + ppm_Na + ppm_Mg + ppm_S + ppm_Na + ppm_Zn +            ppm_Mn +  ppm_Fe + ppm_Cu + ppm_B +   pH + PercentSand + PercentSilt + PercentClay +    (1|newplotid) +                             (1|site_code), comb, REML = F)
summary(MixedMod_Rich )
```

```
## Linear mixed model fit by maximum likelihood  ['lmerMod']
## Formula: 
## log(live_mass) ~ log(rich) + as.factor(country) + as.factor(habitat) +  
##     as.factor(year) + elevation + managed + burned + grazed +  
##     anthropogenic + TEMP_VAR_v2 + MIN_TEMP_v2 + MAX_TEMP_v2 +  
##     TEMP_WET_Q_v2 + TEMP_DRY_Q_v2 + TEMP_WARM_Q_v2 + TEMP_COLD_Q_v2 +  
##     pct_C + pct_N + ppm_P + ppm_K + ppm_Na + ppm_Mg + ppm_S +  
##     ppm_Na + ppm_Zn + ppm_Mn + ppm_Fe + ppm_Cu + ppm_B + pH +  
##     PercentSand + PercentSilt + PercentClay + (1 | newplotid) +  
##     (1 | site_code)
##    Data: comb
## 
##      AIC      BIC   logLik deviance df.resid 
##   1567.9   1829.8   -726.0   1451.9      617 
## 
## Scaled residuals: 
##      Min       1Q   Median       3Q      Max 
## -12.1969  -0.4802   0.0328   0.5756   2.9930 
## 
## Random effects:
##  Groups    Name        Variance Std.Dev.
##  newplotid (Intercept) 0.0000   0.0000  
##  site_code (Intercept) 0.0000   0.0000  
##  Residual              0.5031   0.7093  
## Number of obs: 675, groups:  newplotid, 82; site_code, 28
## 
## Fixed effects:
##                                        Estimate Std. Error t value
## (Intercept)                          -4.933e+01  1.555e+02  -0.317
## log(rich)                             3.796e-01  9.340e-02   4.064
## as.factor(country)CA                  1.430e+01  6.852e+00   2.087
## as.factor(country)CH                  1.132e+01  4.442e+00   2.549
## as.factor(country)DE                  1.203e+01  7.707e+00   1.561
## as.factor(country)TZ                  6.894e-01  8.220e+00   0.084
## as.factor(country)UK                  9.200e+00  7.150e+00   1.287
## as.factor(country)US                  1.040e+01  8.676e+00   1.199
## as.factor(habitat)annual grassland    8.341e+00  8.237e+00   1.013
## as.factor(habitat)desert grassland   -1.198e+00  5.505e+00  -0.218
## as.factor(habitat)mesic grassland     7.824e+00  4.894e+00   1.599
## as.factor(habitat)montane grassland  -2.766e+00  2.327e+00  -1.189
## as.factor(habitat)old field           6.010e+00  6.807e+00   0.883
## as.factor(habitat)pasture            -1.753e-01  4.120e+00  -0.043
## as.factor(habitat)semiarid grassland  1.212e+01  1.422e+01   0.853
## as.factor(habitat)shortgrass prairie  6.020e+00  5.947e+00   1.012
## as.factor(habitat)shrub steppe       -3.000e+00  1.399e+00  -2.144
## as.factor(habitat)tallgrass prairie   6.322e+00  6.664e+00   0.949
## as.factor(year)2008                   5.096e-02  1.309e-01   0.389
## as.factor(year)2009                  -2.486e-02  1.266e-01  -0.196
## as.factor(year)2010                   2.507e-01  1.262e-01   1.987
## as.factor(year)2011                   8.250e-02  1.268e-01   0.651
## as.factor(year)2012                  -3.758e-02  1.282e-01  -0.293
## as.factor(year)2013                   2.008e-01  1.343e-01   1.495
## as.factor(year)2014                  -1.363e-02  1.415e-01  -0.096
## as.factor(year)2015                  -1.006e-01  1.420e-01  -0.708
## as.factor(year)2016                   4.982e-01  1.489e-01   3.345
## as.factor(year)2017                   4.397e-01  1.916e-01   2.295
## elevation                             1.043e-02  6.976e-03   1.495
## managed                              -5.021e+00  3.543e+00  -1.417
## burned                               -1.208e+00  1.100e+00  -1.097
## grazed                                5.342e+00  3.972e+00   1.345
## TEMP_VAR_v2                          -3.565e-01  1.647e-01  -2.164
## MIN_TEMP_v2                          -8.601e-01  9.245e-01  -0.930
## MAX_TEMP_v2                          -2.933e+00  1.274e+00  -2.303
## TEMP_WET_Q_v2                         1.662e-01  1.153e-01   1.442
## TEMP_DRY_Q_v2                         3.821e-01  2.726e-01   1.401
## TEMP_WARM_Q_v2                        1.778e+01  8.191e+00   2.171
## TEMP_COLD_Q_v2                       -1.370e+01  7.812e+00  -1.754
## pct_C                                -1.130e-01  1.162e-01  -0.972
## pct_N                                 3.079e+00  1.749e+00   1.760
## ppm_P                                 1.036e-02  6.256e-03   1.656
## ppm_K                                 8.061e-04  4.968e-04   1.623
## ppm_Na                                6.850e-04  7.857e-04   0.872
## ppm_Mg                               -1.550e-03  5.846e-04  -2.652
## ppm_S                                -7.360e-03  1.177e-02  -0.625
## ppm_Zn                               -1.166e-02  3.772e-02  -0.309
## ppm_Mn                                1.255e-04  2.527e-03   0.050
## ppm_Fe                               -2.945e-03  1.687e-03  -1.745
## ppm_Cu                                1.122e-01  4.728e-02   2.372
## ppm_B                                -1.537e-01  1.712e-01  -0.897
## pH                                   -1.482e-01  1.743e-01  -0.850
## PercentSand                           3.496e-01  1.579e+00   0.221
## PercentSilt                           3.404e-01  1.584e+00   0.215
## PercentClay                           3.403e-01  1.579e+00   0.216
## fit warnings:
## fixed-effect model matrix is rank deficient so dropping 2 columns / coefficients
## Some predictor variables are on very different scales: consider rescaling
## optimizer (nloptwrap) convergence code: 0 (OK)
## boundary (singular) fit: see help('isSingular')
```

Across these models, note that the coefficient on log richness does not really change, and is not very different from the linear model above that include additional covariates. This is because random effects are not really controlling for factors that both correlate with richness and with productivity.

Why ever use random effects? Well, when the assumption of no correlation between the explanatory variable and the random effect holds, random effects estimates are more efficient. But generally, bias is the first order concern with observational data.

# 6 Accounting for other sources of confounding variables

A hallmark of modern approaches to causal inference is to probe the robustness of results to potential violations in the assumptions used to infer causality from correlation.
Using four approaches, we use different assumptions than made in our Main Design (the two-way fixed effects estimator – in Figure 2A) and assess how the conclusions change (see SM). Causal inference in each approach requires causal assumptions, but each approach makes different assumptions. In other words, each approach can detect hidden biases in our design (i.e., threats to internal validity) under different conditions. Our causal interpretations in the paper are strengthened by the robustness of our inferences to changes in the causal assumptions. In other words, it’s not hard to knock one of the analyses as having limitations, but it’s harder to come up with a story that explains the pattern we observe across all of the analyses (including the complementary analyses about species richness as a heterogeneous treatment in Figs 4 and 5). The results are shown in Figure 3 of the main text and the analyses are described in detail in the SM.

TAKEAWAY: The best way to view all of the analyses described in Figure 3 is that each analysis uses different causal assumptions yet comes to the same conclusion. That pattern is the source of our paper’s strength of evidence: the results of each individual analysis could be explained by a different rival explanation, but it is hard to come up with a coherent set of rival explanations that could explain them all. And, if so, the analyses and rival explanation point to a clear direction for future studies to examine.

We provide some examples of the types of confounding variables addressed by these additional analysis designs. These examples are illustrative, not intended to be exhaustive:

**Dynamic Panel model addresses potential biases** from, for example,:
\* Soil fertility or plant-soil feedbacks
\* Dead standing material/litter influencing (especially annual) recruitment in the following growing season
\* Prior plot-specific disturbances or other shocks that are at the plot level
\* Dynamic effects of productivity on richness (across years)
\* Maternal effects
\* etc.

**Sensitivity analysis and Instrumental Variables (IV) Design address potential biases in two different ways** from, for example,

- Micro-climate at the plot level that changes through time
- Insect community composition, pollination communities, herbivory rates
- Measurement error
- Disturbances that are plot specific (e.g., gophers)

**IV and Mechanism blocking designs address potential biases in two different ways** from so-called “reverse causality”

- The effect of within-season productivity on richness (and related pathways such a plant height, shading)

The assumptions for each design are laid out in simple form in Figure 3 in the main text (and see below), and at length in the Supplemental Materials (also see Figure S4, S5, S6, S7, and S8 for visuals).

# 7 Glossary

Bias and Hidden Bias
:   Bias is a property of an estimator of a causal effect: it captures the difference between the estimator’s expected value and the true value of the causal effect being estimated (Wooldridge 2015). The phrase “hidden bias” (also called “unobserved heterogeneity”) is often used to describe the potential sources of bias in a study design (e.g., an omitted third variable that affects both biodiversity and productivity). Hidden bias is thus a rival explanation for detecting or failing to detect a correlation between a purported causal variable and its outcome using observable data (Ferraro and Hanauer 2014). The goal of causal analysis is to choose data and a design so that an actual causal effect would be visibly different from the most plausible hidden biases. Note: sampling variability (“noise”) is different from hidden bias; sampling variability is not a source of bias. Reviewed in (Larsen, Meng, and Kendall 2019) for ecologists.

Cluster standard errors
:   Standard errors that are robust to arbitrary correlation and heteroskedasticity within groups (“cluster”). A standard tool for inference with panel data. See (Wooldridge 2010) and (Cameron and Miller 2015).

Conditioning on Observables
:   Typically used as a synonym for “controlling” for, i.e., a model that conditions on set of observables typically means that it has included those observables linearly as control variables.

Confounding Variables (or “confounders”)
:   In this study, we use the term “confounding variable” to describe variables that are systematically correlated with the causal variable (e.g., biodiversity) and the outcome variable (e.g., productivity), and thus can mask or mimic a causal effect. Confounding variables are a potential source of bias in a **non-experimental** study design.

Cross-sectional Data
:   Data without repeated measures taken from a sample. Instead, one measure is taken from each unit of analysis (e.g., plot). For example, analyses of the effect of biodiversity on productivity in cross-sectional data have only one observation per plot. It is is hard to assume the we observe and correctly control for all confounding variables when analyzing cross-sectional data.

Direct Acyclic Graph
:   Generally, a directed graph (a network with connections in which the direction of flow matters) with no directed cycles (no repeating loops). When working with observational data, a tool used to understand potential confoudning pathways, see e.g., this tutorial.

Endogeniety
:   Broadly, situations in which the variable of interest is correlated with the error term. The goal of causal analysis using observational data is typically to reduce or remove endogeneity. See (Wooldridge 2015); reviewed in (Larsen, Meng, and Kendall 2019) and (Kendall 2015) for ecologists, also
    this video

Errors versus Residuals:
:   The error term is part of the *true* data generating process. In contrast, residuals are the difference between the regression line and the observed data points. Residuals cannot be used to assess potential bias in an estimation procedure.

Identification
:   Formally, a parameter is point identified if it can be uniquely determined from data (usually via a research design). Commonly used to denote whether an estimator of a parameter converges in probability to the value the researcher is seeking; e.g., “Under these three assumptions, \(\beta\) is identified” (meaning it reflects the the value of interest, like a causal effect). See (Larsen, Meng, and Kendall 2019) for a review for ecologists

Instrumental Variable
:   This term is defined in in Figure 1B as variable Z, a variable that affects the treatment variable (in our study, “species richness”) but has no direct effect on the outcome variable (in our study, “above-ground biomass”) except through its effect on the treatment. In a randomized experiment, the instrumental variable is the randomization procedure. To read more, see (Imbens 2014; Kendall 2015) and our SM.

Panel Data
:   Panel data are longitudinal data comprising repeated measures taken from a sample of cases (e.g., plots, sites, regions). Such data are also sometimes referred to as longitudinal multilevel data. See our SM section S3 for more detail on this panel data. For an excellent introduction to approaches using panel data, see (Angrist and Pischke 2008).

Rival Explanations
:   Alternative narratives that can explain estimated relationships but that do not violate identification.

Selection on Observables" Assumption
:   Informally, this assumption implies that confounding variables that could introduce bias into a design are known and observable to the researcher. The bias they introduce into an estimator can thus be eliminated (controlled, blocked) by conditioning strategies, such as regression, matching or stratification methods. To read more, see (Morgan and Winship 2014).

# 8 References

Angrist, Joshua D, and Jörn-Steffen Pischke. 2008. *Mostly Harmless Econometrics: An Empiricist’s Companion*. Princeton university press.

Bell, Andrew, Malcolm Fairbrother, and Kelvyn Jones. 2019. “Fixed and Random Effects Models: Making an Informed Choice.” *Quality & Quantity* 53 (2): 1051–74.

Cameron, A Colin, and Douglas L Miller. 2015. “A Practitioner’s Guide to Cluster- Robust Inference.” *Journal of Human Resouces* 50 (2): 317–72. https://doi.org/10.3368/jhr.50.2.317.

Ferraro, Paul J, and Merlin M Hanauer. 2014. “Advances in Measuring the Environmental and Social Impacts of Environmental Programs.” *Annual Review of Environment and Resources* 39: 495–517.

Ferraro, P J, J N Sanchirico, and M D Smith. 2018. “Causal Inference in Coupled Human and Natural Systems.” *Proceedings of the National Academy of Science*, 201805563. https://doi.org/10.1073/pnas.1805563115.

Fieberg, J, and M Ditmer. 2012. “Understanding the Causes and Consequences of Animal Movement: A Cautionary Note on Fitting and Interpreting Regression Models with Time-Dependent Covariates.” *Methods in Ecology and Evolution* 3 (6): 983–91.

Fukami, Tadashi, and Mifuyu Nakajima. 2011. “Community Assembly: Alternative Stable States or Alternative Transient States?: Alternative Transient States.” *Ecology Letters* 14 (10): 973–84. https://doi.org/10.1111/j.1461-0248.2011.01663.x.

Grace, James B., T. Michael Anderson, Eric W. Seabloom, Elizabeth T. Borer, Peter B. Adler, W. Stanley Harpole, Yann Hautier, et al. 2016. “Integrative Modelling Reveals Mechanisms Linking Productivity and Plant Species Richness.” *Nature* 529 (7586): 390–93. https://doi.org/10.1038/nature16524.

Grace, James B., and Kathryn M. Irvine. 2020. “Scientist’s guide to developing explanatory statistical models using causal analysis principles.” *Ecology* 101 (4): 1–14. https://doi.org/10.1002/ecy.2962.

Imbens, Guido. 2014. “Instrumental Variables: An Econometrician’s Perspective.” National Bureau of Economic Research.

Isbell, F., P. B. Reich, D. Tilman, S. E. Hobbie, S. Polasky, and S. Binder. 2013. “Nutrient Enrichment, Biodiversity Loss, and Consequent Declines in Ecosystem Productivity.” *Proceedings of the National Academy of Sciences* 110 (29): 11911–6. https://doi.org/10.1073/pnas.1310880110.

Kendall, Bruce E. 2015. *A Statistical Symphony: Instrumental Variables Reveal Causality and Control Measurement Error*.

Larsen, Ashley E, Kyle Meng, and Bruce E Kendall. 2019. “Causal Analysis in Control–Impact Ecological Studies with Observational Data.” *Methods in Ecology and Evolution* 10 (7): 924–34.

Loreau, Michel. 1998. “Biodiversity and Ecosystem Functioning: A Mechanistic Model.” *Proceedings of the National Academy of Sciences of the United States of America* 95 (10): 5632–6. https://doi.org/Doi 10.1073/Pnas.95.10.5632.

Morgan, Stephen L., and Christopher Winship. 2014. *Counterfactuals and Causal Inference: Methods and Principles for Social Research*. Second. Cambridge: Cambridge University Press. https://doi.org/10.1017/CBO9781107587991.

Pearl, Judea. 2009. *Causality*. Cambridge university press.

———. 2011. “Aspects of Graphical Models Connected with Causality.”

Schoolmaster Jr, Donald R, James B Grace, E William Schweiger, Brian R Mitchell, and Glenn R Guntenspergen. 2013. “A Causal Examination of the Effects of Confounding Factors on Multimetric Indices.” *Ecological Indicators* 29: 411–19.

Schoolmaster Jr, Donald R, Chad R Zirbel, and James Patrick Cronin. 2020. “A Graphical Causal Model for Resolving Species Identity Effects and Biodiversity–Ecosystem Function Correlations.” *Ecology* 101 (8): e03070.

Wooldridge, Jeffrey M. 2010. *Econometric Analysis of Cross Section and Panel Data*. MIT press.

———. 2015. *Introductory Econometrics: A Modern Approach*. Cengage learning.
