# Supplementary material for: Clarifying the effect of biodiversity on productivity in natural ecosystems with longitudinal data and methods for causal inference
Source: Nat Commun. 2023 May 5;14:2607. doi: 10.1038/s41467-023-37194-5 (PMC10163230; doi:10.1038/s41467-023-37194-5)
Supplement: Supplementary file 4 — Reporting Summary [file 41467_2023_37194_MOESM4_ESM.pdf]

nature portfolio

Corresponding author(s): Laura E. Dee, Paul J. Ferraro

Last updated by author(s): Nov 9, 2022

Reporting Summary

Nature Portfolio wishes to improve the reproducibility of the work that we publish. This form provides structure for consistency and transparency in reporting. For further information on Nature Portfolio policies, see our Editorial Policies and the Editorial Policy Checklist.

Statistics

For all statistical analyses, confirm that the following items are present in the figure legend, table legend, main text, or Methods section.

n/a	Confirmed	
[ ]	[x]	The exact sample size (n) for each experimental group/condition, given as a discrete number and unit of measurement
[ ]	[x]	A statement on whether measurements were taken from distinct samples or whether the same sample was measured repeatedly
[ ]	[x]	The statistical test(s) used AND whether they are one- or two-sided<br>*Only common tests should be described solely by name; describe more complex techniques in the Methods section.*
[ ]	[x]	A description of all covariates tested
[ ]	[x]	A description of any assumptions or corrections, such as tests of normality and adjustment for multiple comparisons
[ ]	[x]	A full description of the statistical parameters including central tendency (e.g. means) or other basic estimates (e.g. regression coefficient) AND variation (e.g. standard deviation) or associated estimates of uncertainty (e.g. confidence intervals)
[ ]	[x]	For null hypothesis testing, the test statistic (e.g. F, t, r) with confidence intervals, effect sizes, degrees of freedom and P value noted<br>*Give P values as exact values whenever suitable.*
[x]	[ ]	For Bayesian analysis, information on the choice of priors and Markov chain Monte Carlo settings
[ ]	[x]	For hierarchical and complex designs, identification of the appropriate level for tests and full reporting of outcomes
[ ]	[x]	Estimates of effect sizes (e.g. Cohen's d, Pearson's r), indicating how they were calculated

Our web collection on statistics for biologists contains articles on many of the points above.

Software and code

Policy information about availability of computer code

Data collection	No software was used to obtain data for this study.
Data analysis	The data and code for reproducing all analyses, figures, and tables in this study are available at https://github.com/LauraDee/NutNetCausalinf [the data and code were also provided to reviewers in a separate uploaded zip file]. All analyses were performed in R and STATA software. An RMarkdown tutorial on the methods can also be found at our Github project page [and was uploaded for the reviewers]

For manuscripts utilizing custom algorithms or software that are central to the research but not yet described in published literature, software must be made available to editors and reviewers. We strongly encourage code deposition in a community repository (e.g. GitHub). See the Nature Portfolio guidelines for submitting code & software for further information.

Data

Policy information about availability of data

All manuscripts must include a data availability statement. This statement should provide the following information, where applicable:

- Accession codes, unique identifiers, or web links for publicly available datasets
- A description of any restrictions on data availability
- For clinical datasets or third party data, please ensure that the statement adheres to our policy

The data and code for reproducing all analyses, figures, and tables in this study are available at https://github.com/LauraDee/NutNetCausalinf and were provided to the referees. Data were originally obtained via a request to the Nutrient Network, nutnet.org.

Field-specific reporting

Please select the one below that is the best fit for your research. If you are not sure, read the appropriate sections before making your selection.

☐ Life sciences ☐ Behavioural & social sciences ☒ Ecological, evolutionary & environmental sciences

For a reference copy of the document with all sections, see nature.com/documents/nr-reporting-summary-flat.pdf

Ecological, evolutionary & environmental sciences study design

All studies must disclose on these points even when the disclosure is negative.

Study description	Here, we address the question of how plant species diversity affects productivity by developing a design that leverages longitudinal data from grasslands around the world and new approaches to causal inference with observational data, borrowed from disciplines outside of ecology. Specifically, we use repeated observations between 2007-2017 from 151 unmanipulated plots in 43 grassland sites in 11 countries. Data from multiple years offer three advantages: 1) an opportunity to study natural changes in richness; 2) enhanced external validity; and 3) ways to control for a broad set of confounding variables, including unobserved ones. Our study differs from prior ecology publications by combining three features: 1) causal diagrams to inform the design and transparently communicate the assumptions required for inferring a causal relationship from a correlation; 2) regression models that leverage repeated observations on the same plots and sites to control for confounding variables, both observable and unobservable; and 3) rigorous assessments of the robustness of our inferences to violations of the assumptions required for inferring causal relationships from the data. The SI offers a primer on these ideas and compares them to approaches widely used in ecology.
Research sample	We analyze panel data from grasslands around the world in the Nutrient Network (nutnet.org) which includes mesic grasslands and prairies, savanna, desert grasslands, montane meadows, old fields, and alpine tundra. We use data from 43 sites with unmanipulated plots with at least 5 years of data in the period 2007-2017 (for site details, see Table S1). Unmanipulated plots are control plots in the nutrient addition experiments of the Network, meaning they receive no additional nutrients. Unprocessed data versions were 'full-cover-09-April-2018.csv', and 'comb-by-plot-clim-soil-diversity-09-Apr-2018.csv' from the Nutrient Network. All R scripts to process data and create derived data is available at the project GitHub page.
Sampling strategy	We use data from all unmanipulated plots in the Nutrient Network that have at least five years of data in the period 2007-2017.
Data collection	The data were collected by participants in the Nutrient Network, following the Network's data collection protocol (nutnet.org).
Timing and spatial scale	Data are from unmanipulated plots that are nested (clustered) within 43 sites and include at least five years of data in the period 2007-2017
Data exclusions	We exclude manipulated plots from the Nutrient Network because our study is of natural systems, not experimentally manipulated systems. We exclude unmanipulated plots with fewer than five years of data because our research design depends on longitudinal data to control for confounding variables
Reproducibility	Every piece of code was checked by at least two coauthors for reproducibility, including code used to process the data and run each analysis. We also performed the main analyses in both R and STATA, performed by separately by different authors, to ensure the results are reproducible. For full reproducibility, all code used in this research for R and STATA are available https://github.com/LauraDee/NutNetCausalinf .
Randomization	No
Blinding	No

Did the study involve field work? ☐ Yes ☒ No

Reporting for specific materials, systems and methods

We require information from authors about some types of materials, experimental systems and methods used in many studies. Here, indicate whether each material, system or method listed is relevant to your study. If you are not sure if a list item applies to your research, read the appropriate section before selecting a response.

Materials & experimental systems

n/a	Involved in the study
☒	☐ Antibodies
☒	☐ Eukaryotic cell lines
☒	☐ Palaeontology and archaeology
☒	☐ Animals and other organisms
☒	☐ Human research participants
☒	☐ Clinical data
☒	☐ Dual use research of concern

Methods

n/a	Involved in the study
☒	☐ ChIP-seq
☒	☐ Flow cytometry
☒	☐ MRI-based neuroimaging
